# Prostaglandin E_2_ promotes post-infarction cardiomyocyte replenishment by endogenous stem cells

**DOI:** 10.1002/emmm.201303687

**Published:** 2014-01-21

**Authors:** Ying-Chang Hsueh, Jasmine M F Wu, Chun-Keung Yu, Kenneth K Wu, Patrick C H Hsieh

**Affiliations:** 1 Institute of Basic Medical Sciences, National Cheng Kung University and HospitalTainan, Taiwan; 2 Institute of Clinical Medicine, National Cheng Kung University and HospitalTainan, Taiwan; 3 Department of Microbiology and Immunology, National Cheng Kung University and HospitalTainan, Taiwan; 4 Institute of Cellular and System Medicine, National Health Research InstitutesMiaoli, Taiwan; 5 Institute of Biomedical Sciences, Academia SinicaTaipei, Taiwan

**Keywords:** aging, cardiac regeneration, cyclooxygenase 2, genetic fate-mapping, inflammation

## Abstract

Although self-renewal ability of adult mammalian heart has been reported, few pharmacological treatments are known to promote cardiomyocyte regeneration after injury. In this study, we demonstrate that the critical period of stem/progenitor cell-mediated cardiomyocyte replenishment is initiated within 7 days and saturates on day 10 post-infarction. Moreover, blocking the inflammatory reaction with COX-2 inhibitors may also reduce the capability of endogenous stem/progenitor cells to repopulate lost cells. Injection of the COX-2 product PGE_2_ enhances cardiomyocyte replenishment in young mice and recovers cell renewal through attenuating TGF-β1 signaling in aged mice. Further analyses suggest that cardiac stem cells are PGE_2_-responsive and that PGE_2_ may regulate stem cell activity directly through the EP2 receptor or indirectly by modulating its micro-environment *in vivo*. Our findings provide evidence that PGE_2_ holds great potential for cardiac regeneration.

## Introduction

Growing studies have demonstrated that the adult mammalian heart preserves a self-renewal capacity and resides various stem/progenitor cell populations (Ellison Georgina *et al*, 2013; Hoch *et al*, 2011; Laugwitz *et al*, 2005; Oh *et al*, 2003; Pfister *et al*, 2005; Rota *et al*, 2007; Smart *et al*, 2011; Smith *et al*, 2007). Also, approximately 20% of cardiomyocytes are replenished by endogenous stem/progenitor cells in the peri-infarct border zone after myocardial infarction (MI) in mice (Hsieh *et al*, 2007; Loffredo Francesco *et al*, 2011; Malliaras *et al*, 2013; Senyo *et al*, 2013). Interestingly, a recent study by Senyo *et al* reported that ∼20% of pre-existing cardiomyocytes at the border zone undergo cell cycle although among them, only 3.2% of cells complete the cell division (Senyo *et al*, 2013). These results imply that the majority of replenished cardiomyocytes may be originated from the endogenous stem/progenitor cells. Nevertheless, a therapy promoting endogenous stem cells to repair injury after MI, including systemic delivery of drugs, is still lacking. It also remains unclear the most critical time period to activate the stem cell-driven cardiomyocyte replenishment. Answers to these questions will offer opportunities for developing new *de novo* treatments.

In this study, we used the cardiac specific tamoxifen-inducible Cre-*LoxP* MerCreMer/ZEG (M/Z) transgenic mice to delineate the underlying mechanism initiating stem/progenitor cell-modulated cardiac repair and to investigate the regenerative efficiency in young and aged mice. Furthermore, we aimed to identify a pharmacological intervention that improves the cardiac repair efficiency after MI.

## Results

### Endogenous stem/progenitor cell-mediated cardiomyocyte replenishment is initiated within 7 days post-MI

**Figure 1:**
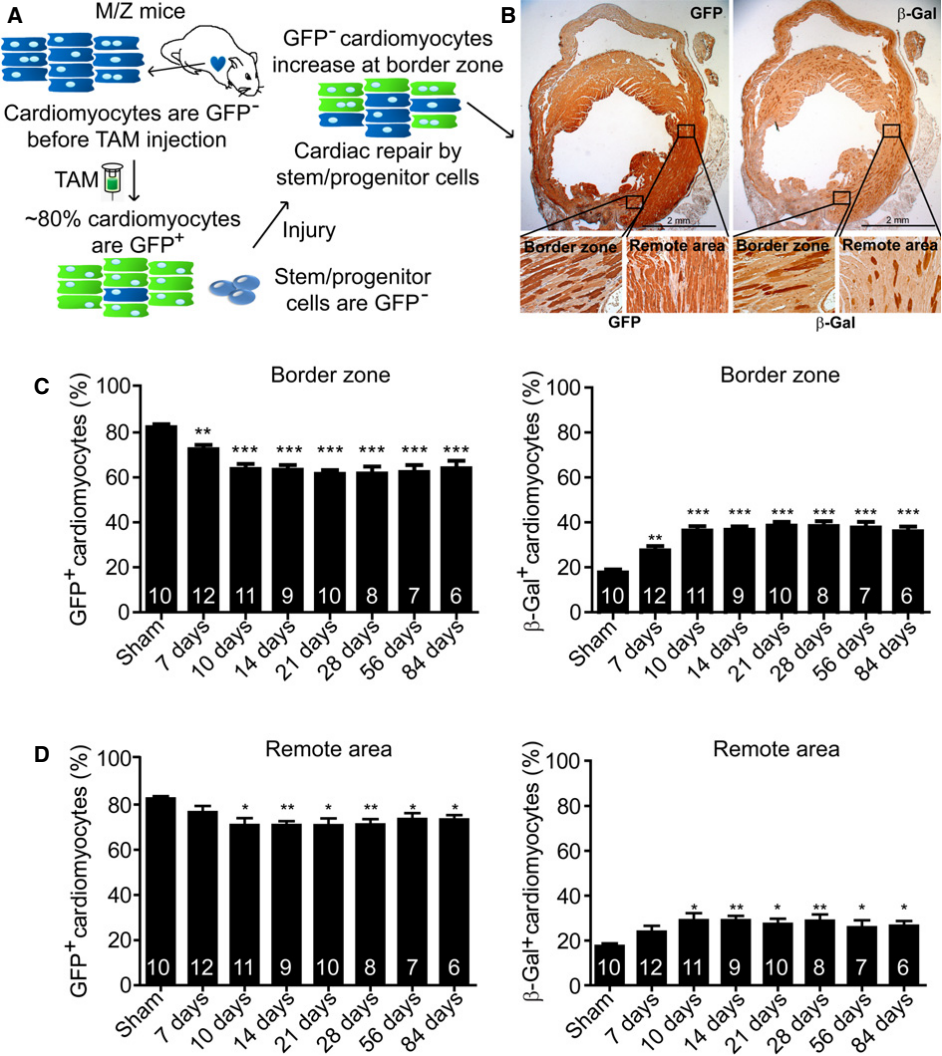
Replenishment of adult mouse cardiomyocytes by endogenous stem/progenitor cells occurs within 7 days and is saturated on day 10 following infarction. A Schematic diagram of adult cardiomyocyte fate-mapping using MerCreMer/ZEG (M/Z) mice. B Enlarged images of GFP^+^ and β-Gal^+^ cardiomyocytes at the infarct border zone and remote area. C, D GFP^+^ and β-Gal^+^ cardiomyocytes at the border zone (C) or the remote area (D) were quantified. *P < 0.05, **P < 0.01, *** *P *< 0.001 versus sham. Error bars: s.e.m. Sample size is indicated in the bar chart. TAM, Tamoxifen.

To determine the most critical time period for cardiomyocyte replenishment, we used the M/Z mice to trace endogenous stem/progenitor cell-driven cardiomyocyte replenishment upon injury (Fig 1A and B, Supplementary Fig S1) (Hsieh *et al*, 2007; Loffredo Francesco *et al*, 2011; Malliaras *et al*, 2013; Senyo *et al*, 2013). Compared with the sham group, there were ∼10% and ∼20% of GFP^-^ cardiomyocytes renewed at the border zone on day 7 and day 10 post-MI, respectively (Fig 1C). The cell replacement saturated on day 10 and leveled off until the third month after MI. β-Gal staining and cell quantification in the remote area showed consistent results (Fig 1C and D). These findings are in agreement with previous reports showing that transplanted or resident cardiac stem cells are capable of differentiating into cardiomyocytes within 7–14 days after MI (Oh *et al*, 2003; Rota *et al*, 2007; Smart *et al*, 2011).

### Early COX-2 activity is required for post-infarction cardiomyocyte replenishment

**Figure 2:**
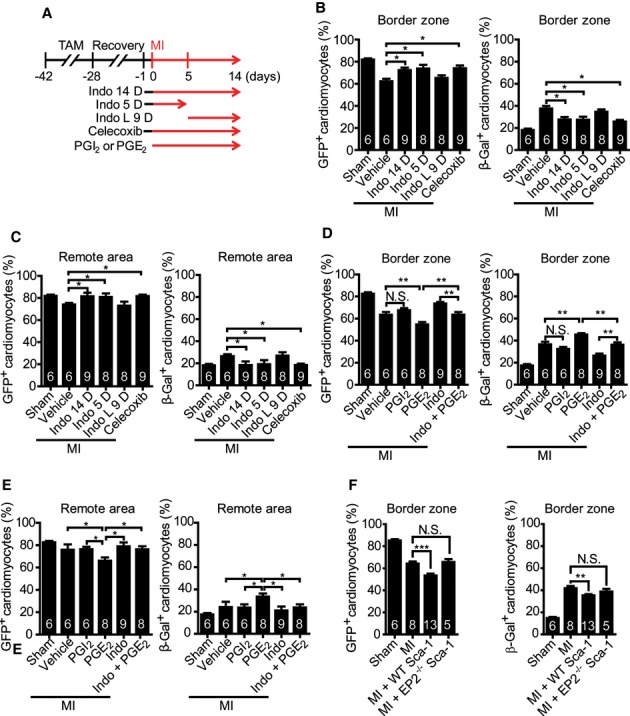
COX-2-dependent signaling pathway stimulates cardiomyocyte replenishment by endogenous stem/progenitor cells shortly after infarction. A Experimental paradigm for drug treatment of the MerCreMer/ZEG mice. Indomethacin (Indo) and Celecoxib were administered from 1 day before to day 14 (Indo 14 D and Celecoxib) or day 5 (Indo 5 D) after MI or from day 6 to day 14 post-MI (Indo last 9 days or Indo L 9 D). PGI_2_ and PGE_2_ were administered continuously for 14 days. The hearts from drug-treated mice isolated on day 14 post-infarction were stained for GFP or β-Gal. B–E GFP^+^ and β-Gal^+^ cardiomyocytes observed at the border zone (B and D) and in the remote area (C and E) were quantified and statistically analysed. F Following DAB staining, the percentages of GFP^+^ and β-Gal^+^ cardiomyocytes at the border zone of the young heart with or without cell injection after MI were quantified and statistically analyzed. Data information: *P < 0.05, **P < 0.01, ***P < 0.001; N.S., not significant. Data are presented as the mean ± s.e.m. Sample size is indicated in the bar chart. TAM, Tamoxifen; MI, myocardial infarction.

MI-induced cyclooxygenase (COX)-2 expression and prostaglandin E_2_ (PGE_2_) production in the heart provide a cardiac protective effect (Degousee *et al*, 2008; Wang *et al*, 2009; Wong *et al*, 1998; Xiao *et al*, 2004). To explore its connection with cardiomyocyte repopulation, mice were treated with Indomethacin, a pan-COX pathway inhibitor (Goessling *et al*, 2009) (Fig 2A). At the border zone, the cardiomyocyte restoration rate dropped by ∼10% upon Indomethacin administration (Fig 2B, Supplementary Fig S2). The same blocking effect was also observed in the remote area (Fig 2C). Furthermore, Indomethacin given during the first 5 days post-MI (Indo 5 D) sufficiently impaired cardiomyocyte replenishment at the border zone. However, it had no significant effect when given on day 6-14 post-MI (Indo L 9 D). Cardiomyocyte replenishment was also abolished upon treatment of Celecoxib, a selective COX-2 inhibitor (Lyons *et al*, 2011), for 14 days or within 5 days post-MI (Fig 2A and B, Supplementary Fig S3A and B).

### PGE_2_ promotes cardiomyocyte replenishment after infarction

Next, the mice were treated with the COX-2 downstream effectors prostaglandin E_2_ (PGE_2_), a pharmacological agent involved in stem cell-mediated tissue regeneration after injury (Goessling *et al*, 2009; Li *et al*, 2010) whose level increases in the heart after MI (Degousee *et al*, 2008). Our results indicated that treatment of PGE_2_, but not PGI_2_, significantly increased cardiomyocyte replenishment at the border zone by ∼9% (Fig 2A and D, Supplementary Fig S2) and rescued cardiomyocyte repopulation by ∼10% compared to that in Indomethacin or Celecoxib treatment alone (Fig 2B and D, Supplementary Fig S3B). A similar trend was observed in the remote area (Fig 2D and E). These findings imply that an early COX-2/PGE_2_ signaling is required for the induction of stem cell-driven cardiomyocyte replenishment.

### PGE_2_ regulates cardiac stem cell differentiation

To determine whether PGE_2_ acts on cardiac stem/progenitor cells to promote cardiomyocyte differentiation, expression level of several known cardiac stem/progenitor cell markers quantified for identification of the PGE_2_-responsive gene. We discovered that Sca-1 expression peaked on day 3 post-MI and this level was further increased at the same time point upon PGE_2_ treatment but was repressed by Indomethacin (Supplementary Fig S4). Sca-1 is a common marker co-expressed by several known cardiac stem/progenitor cell populations (Matsuura *et al*, 2009; Oh *et al*, 2003; Smart *et al*, 2011; Sturzu & Wu, 2011), for example c-Kit^+^ cell population (Bailey *et al*, 2012; Rosenblatt-Velin *et al*, 2012). The c-Kit^+^ cells originated from the heart or bone marrow are shown to possess cardiac repair capability (Ellison Georgina *et al*, 2013; Loffredo Francesco *et al*, 2011; Orlic *et al*, 2001; Rota *et al*, 2007). However, their ability to repair heart is attenuated upon loss of Sca-1 (Bailey *et al*, 2012; Rosenblatt-Velin *et al*, 2012). Furthermore, we observed that the expression pattern of the cardiac progenitor cell marker *Nkx2.5* (Wu *et al*, 2006) is similar to that of *Sca-1* (Supplementary Fig S5). Furthermore, PGE_2_ also elevated the expression of *Nkx2.5* in Sca-1^+^ cells (Supplementary Fig S6). We therefore sought to investigate the effect of PGE_2_ on stem cell-mediated cardiomyocyte replenishment by examining Sca-1^+^ cell activities.

Because tamoxifen injection in M/Z mice leads to conversion of β-Gal to GFP in cardiomyocytes, we thought to take this advantage to examine cardiomyogenic differentiation ability of the cardiac Sca-1^+^ cells. The tamoxifen injection was given to the M/Z mice after MI surgery, and therefore, only α-MHC^+^ cells would express GFP (Supplementary Fig S7A). This experiment allowed us to determine whether Sca-1^+^ cells possess the ability to differentiate into α-MHC^+^ cells. Following MI surgery and tamoxifen injection for 3 days, Sca-1^+^/GFP^+^ cells could be detected. The percentage of double positive cells was further increased upon PGE_2_ treatment (Supplementary Fig S7B and C). In addition, Sca-1^+^/α-MHC^+^ cells were not observed before tamoxifen labeling and they do not arise from cardiomyocyte de-differentiation or fusion (Hsieh *et al*, 2007; Senyo *et al*, 2013) (Supplementary Fig S8A and B). These results reveal the potential contribution of cardiac Sca-1^+^ stem/progenitor cells to cardiomyocyte replenishment after MI.

Following MI, M/Z system serves as a platform to assess the cardiomyocytes differentiated from endogenous stem/progenitor cells. To evaluate the cardiomyocyte differentiation ability of cardiac Sca-1^+^ cells and the importance of PGE_2_ pathway during this process, the cells were isolated from wild-type and EP2^–/–^ mice (Kennedy *et al*, 1999) for intramyocardial injection after MI surgery (Loffredo Francesco *et al*, 2011) (Supplementary Fig S11A). The EP2^−/−^ transgenic mouse was chosen due to the expression of this PGE_2_ receptor was significantly induced in hearts after MI and in cardiac Sca-1^+^ cells after PGE_2_ treatment (Supplementary Fig S9 and S10). Quantification of the GFP^+^ and β-Gal^+^ cardiomyocyte numbers revealed that injection of wild-type Sca-1^+^ cells reduced both GFP^+^ and β-Gal^+^ cardiomyocyte numbers and that approximately 10% of the peri-infarct cardiomyocytes were GFP^–^ and β-Gal^–^, suggesting cardiomyocyte differentiation of the injected cardiac Sca-1^+^ cells. In contrast, we did not observe such change in the M/Z mice receiving injection of EP2^–/–^ Sca-1^+^ cells (Fig 2F, Supplementary Fig S11). Together these results indicate that the PGE_2_/EP2 signaling may regulate the ability of cardiac Sca-1^+^ cells to differentiate into cardiomyocytes. Results from *in vitro* culture also provided evidence that the expression of *Nkx2.5* and *cTnT* was evidently improved in isolated cardiac small cells (cardiomyocyte-depleted cell fraction) and Sca-1^+^ cells by PGE_2_ (Supplementary Fig S12B and C). Surprisingly, mature sarcomeric structure and spontaneously beating cells were seen in the cardiomyocyte-depleted small cells after PGE_2_ treatment (Supplementary Fig S12A and B, Movie S1), suggesting PGE_2_ may improve cardiomyocyte differentiation.

### PGE_2_ modulates the post-infarction inflammatory response in the myocardium

PGE_2_ used to be considered as a pro-inflammatory molecule. However, it has been suggested that PGE_2_ may modulate the inflammatory microenvironment for tissue regeneration through regulating macrophage subtypes (Nemeth *et al*, 2009). Macrophages can be classified into M1 (CD45^+^CD11b^+^F4/80^+^Gr-1^+^) and M2 (CD45^+^CD11b^+^F4/80^+^CD206^+^) subtypes (Nishimura *et al*, 2009; Vandanmagsar *et al*, 2011). Interestingly, flow cytometry analysis revealed that PGE_2_ treatment elevated the number of M2 macrophages but reduced the number of M1 subtype after MI (Supplementary Fig S13A–C). Furthermore, quantitative RT-PCR indicated that PGE_2_ enhanced the expression of *interleukin-10* (*IL-10*) (Nemeth *et al*, 2009), which is modulated by M2 macrophages (Nishimura *et al*, 2009; Vandanmagsar *et al*, 2011) (Supplementary Fig S14). Therefore, we speculate that PGE_2_ acts directly on not only the progenitor/stem cells but also the inflammatory cells such as macrophages to regulate the inflammatory microenvironment after MI.

### PGE_2_ rescues the cardiomyocyte regeneration capacity of aged mice

**Figure 3:**
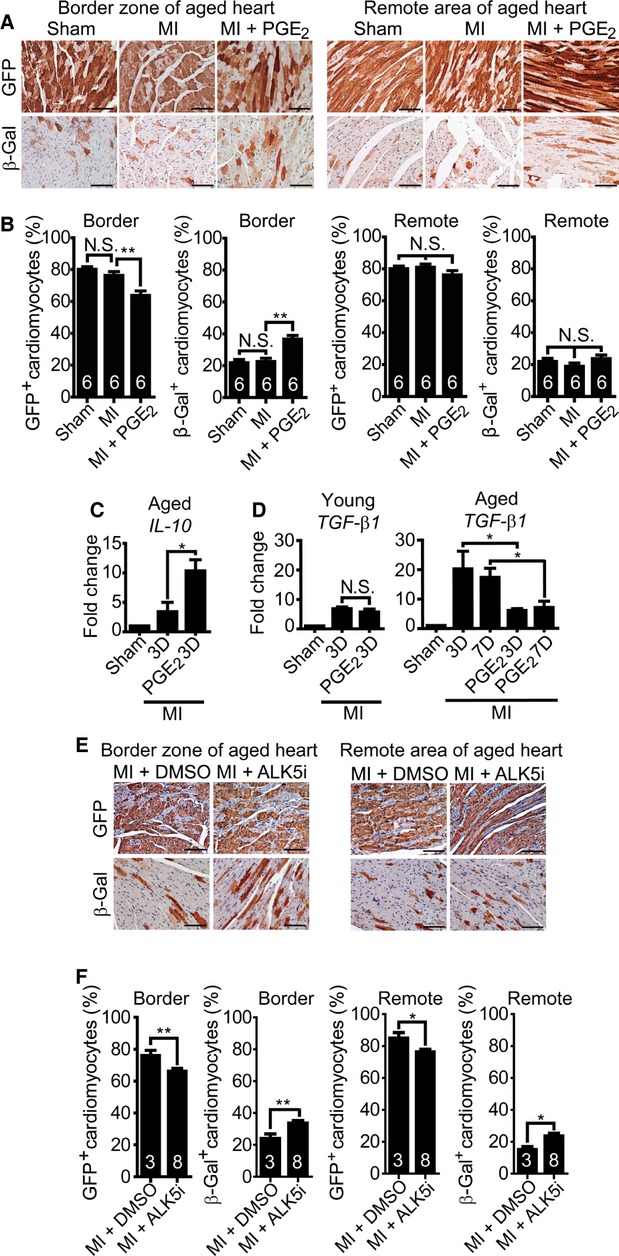
PGE2 treatment restores cardiomyocyte replenishment in aged mice. A, B Fourteen days post-MI, the hearts from aged mice (>18 months) were stained for GFP or β-Gal. Shown are representative images (A) and quantification (B) of GFP^+^ and β-Gal^+^ cardiomyocytes at the border zone and remote area. C Quantitative RT-PCR analysis of *IL-10* expression in response to PGE_2_ treatment on day 3 after infarction at the injured region of old mice. *n* ≥ 3. D Effect of PGE_2_ on *TGF-β1* expression in the infarcted region of MI hearts in young and aged mice, examined on day 3 and day 7 after injury. *n* ≥ 3. E, F Vehicle or ALK5i-treated hearts were harvested on day 14 post-MI for GFP and β-Gal staining. Shown are representative images (E) and quantification (F) of GFP^+^ and β-Gal^+^ cardiomyocytes at the border zone and remote area. Data information: *P < 0.05. **P < 0.01; N.S., not significant. All scale bars: 100 μm. Sample size is indicated in the bar chart.

Because the aged heart loses its regenerative ability (Senyo *et al*, 2013), we examined the degree of cardiomyocyte regeneration in old mice. In aged mice (>18 months), regardless of the same GFP labeling efficiency, MI itself did not induce evident cardiomyocyte replenishment at the border zone (Fig 3A and B). Surprisingly, PGE_2_ treatment successfully rescued the attenuated stem cell-mediated cardiomyocyte reconstitution at the border zone, but not in the remote area (Fig 3A and B). PGE_2_ also increased *IL-10* expression in aged hearts (Fig 3C). Further investigation revealed that the expression of the aging-associated marker gene *transforming growth factor β-1* (*TGF-β1*) (Carlson *et al*, 2008; Luo *et al*, 2010) declined in aged mice following PGE_2_ treatment (Fig 3D). Finally, administration of TGF-β Type I Receptor Kinase Inhibitor II (ALK5 Inhibitor II, ALK5i) (Ichida *et al*, 2009) restored cardiomyocyte replenishment in old mice (Fig 3E and F). Together these results suggest that high TGF-β1 activity may negatively regulate cardiomyocyte replenishment in aged hearts. Therefore, PGE_2_ not only augments cardiomyocyte replenishment in young mice but also rescues the self-regenerative function in aged mice.

## Discussion

Although various cardiac stem/progenitor cell populations capable of differentiating into cardiomyocytes have been identified, a fundamental question remains unanswered. What initiation signal stimulates these cells to generate functional cardiomyocytes *in situ* after injury? It has been demonstrated that the deletion of COX-2 (Wang *et al*, 2009) or microsomal PGE_2_ synthase-1 (mPGES-1) (Degousee *et al*, 2008) adversely affects cardiac function after injury, suggesting a cardioprotective role of the COX-2 pathway. These findings are in line with our results where the inhibition of the COX-2 pathway abrogates tissue repair mediated by endogenous stem/progenitor cells. Importantly, we provide evidence that early inflammatory response plays a key role in this process (Essers *et al*, 2009; Kyritsis *et al*, 2012; Li *et al*, 2010). Furthermore, we discover that PGE_2_ treatment not only augments the efficiency of cardiomyocyte repopulation but also induces redistribution of M1/M2 macrophage ratio, implying the importance of PGE_2_-modulated inflammatory microenvironment in this process (Nemeth *et al*, 2009).

The role of PGE_2_ in modulating stem cell function has been reported in the bone marrow, where it regulates hematopoietic stem cell (HSC) homeostasis (North *et al*, 2007) and improves their functions, including survival and proliferation (Hoggatt *et al*, 2009). A recent study reported by Hoggatt *et al* demonstrates that PGE_2_ facilitates retention of HSCs in the bone marrow and non-steroidal anti-inflammatory drug (NSAID) induces HSC egress (Hoggatt *et al*, 2013). Because Indomethacin and Celecoxib are NSAIDs, they may exert the same effect on HSCs. Based on these results, we suspect that the microenvironment in the cardiac infarct could be disturbed upon mobilization of un-differentiated HSCs, and consequently attenuates cardiomyocyte regeneration efficiency. However, administration of PGE_2_ restores this regenerative machinery by acting on the cardiac stem/progenitor cells and inflammatory cells. We provide evidence to demonstrate that PGE_2_ directly regulates cardiac Sca-1^+^ cells, implying a possible role of NSAID in mediating cardiac stem/progenitor cell mobilization. In addition, PGE_2_ also increases the number of M2 macrophages. Based on these findings and previous studies showing PGE_2_-dependent modulation of HSC activities, we speculate that how PGE_2_ regulates HSCs after MI is also an important factor for cardiomyocyte regeneration.

On the basis of Senyo's findings, several commentary articles have pointed out that the contribution of stem/progenitor cells to cardiac repair may be negligible (Mummery & Lee, 2013). Despite the use of ^15^N labeling system, one question that remains unsolved is the dilution of the GFP^+^ cardiomyocyte pool in the M/Z mice after MI (Senyo *et al*, 2013). Results in our study and others have demonstrated that the number of cardiomyocytes replenished by endogenous stem/progenitor cells at the infarct border zone is greater than the number of cells derived from the dividing pre-existing cardiomyocytes (Loffredo Francesco *et al*, 2011; Malliaras *et al*, 2013). Here, the results also reveal that the ability of stem/progenitor cells to give rise to cardiomyocytes could be modulated, suggesting a potential therapeutic application of the endogenous stem/progenitor cells for cardiac repair.

## Materials and Methods

### Mouse breeding

All experiments involving animals were conducted in accordance with the Guide for the Use and Care of Laboratory Animals, and all animal protocols have been approved by National Cheng Kung University. EP2^−/−^, B6.129-*Ptger2*^*tm1Brey*^/J mice were obtained from Jackson Laboratory. The double transgenic *MerCreMer/ZEG* (M/Z) mice were generated by crossbreeding MerCreMer and Z/EG mice (Jackson Laboratory), which have C57BL/6SV129 and C57BL/6J (N7) background strains, respectively. The MerCreMer mice contain a tamoxifen-inducible Cre recombinase fusion protein driven by the cardiomyocyte-specific *α-MHC* promoter. In Z/EG mice, GFP replaces constitutive β-Gal expression after the removal of a *LoxP*-flanked stop sequence by Cre.

### Surgery

M/Z mice were subjected to experimental myocardial infarction (MI) 1 month after the last tamoxifen injection. MI was generated by ligating the left anterior descending coronary artery at 2–3 mm distal to the left atrial appendage. For immunohistological studies, mice were sacrificed and the hearts were harvested at different time points after MI surgery.

### Drug treatment

To induce Cre recombination to achieve GFP labeling of cardiomyocytes, tamoxifen (Sigma) was dissolved in sunflower oil (Sigma) at a concentration of 5 mg/ml. The tamoxifen solution was injected intraperitoneally into M/Z mice daily at a dosage of 40 μg per 1 g body weight for 14 days. All experimental conditions were optimized prior to the PGE_2_, indomethacin and TGF-β Type I Receptor Kinase Inhibitor II (ALK5 Inhibitor II, 2-(3-(6-Methylpyridin-2-yl)-1H-pyrazol-4-yl)-1,5-naphthyridine, Merck) treatments. The mice treated with PGE_2_ or PGI_2_ (both from Sigma) were injected intraperitoneally with 3.33 ng of drug per 1 g of body weight dissolved in absolute ethanol twice daily. For the Indomethacin treatment, mice were fed with water containing Indomethacin (Sigma, 15 μg/ml) for different periods of time. The Indomethacin-containing water was changed every 3 days. The mice subjected to the Celecoxib (Sigma) treatment were injected intraperitoneally with 5 μg of drug per 1 g of body weight daily. For ALK5i treatment, aged mice were injected intraperitoneally once per day with 1 μg of drug per 1 g of body weight 1 day before surgery and continuously until day 10 post-MI. Celecoxib and ALK5i were dissolved in ethanol and DMSO, respectively.

### GFP^+^ or β-Gal^+^ cardiomyocyte counting

All of the cellular quantifications were performed double-blindly to minimize personal bias. To achieve this, photo taken from the scar tissue was avoided so that the personnel performing cell quantification did not know if the photos were taken from the border zone or the remote area. For the cardiomyocyte cell counts, three sections from each heart, and 2 infarction border zones and 1 remote area from each section were analyzed at a magnification of 200× by light microscopy. Cells with visible sarcomere structures were analyzed, and the average number of cells counted was 171.8 ± 5.8 per photo image. For the small cardiac cell counts, more than five sections from each heart were analyzed at a magnification of 400× using fluorescence microscopy. The average number of cells counted was 17.01 ± 0.99 per photo image, and more than one hundred and fifty cells were analyzed from each heart. As quantification result is the averaged values calculated from the pictures taken from six border zone sections per heart, personal variation has been minimized.

### Immunohistochemistry and immunofluorescence microscopy

The harvested hearts were fixed with 4% paraformaldehyde and embedded in paraffin. The sections were then immunostained with the following primary antibodies: mouse anti-GFP (1:500; MBL), rabbit anti-GFP (1:200; Abcam or GeneTex), rabbit anti-β-Gal (1:500; Invitrogen), mouse anti-cTnT (1:100; DSHB), and rat anti-Sca-1-PE (1:500; BD Bioscience). A DAB substrate kit (Vector Laboratories) was used for immunohistochemistry and appropriate secondary antibodies (Invitrogen or Abcam) were used for visualization under a fluorescence microscope. The plasma membrane was immunostained with wheat germ agglutinin (WGA, 5 μg/ml, Invitrogen) and 4,6-diamidino-2-phenylindole (DAPI, 1 μg/ml; Sigma) was used for nucleus staining.

### Extraction and preparation of total RNA for quantitative real-time PCR

The total RNA isolated from the ischemic region of MI hearts was reverse transcribed using the SuperScript III (Invitrogen) system according to the manufacturer's protocol. For quantitative PCR, the SYBR Green reagent (Maestrogen) was used according to the manufacturer's protocol. The analysis of relative gene expression was performed using the 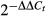 method. The sequence-specific primers designed for semi-quantitative PCR and real-time RT-PCR is listed in supplementary Table S1.

### Flow cytometry, cell isolation, culturing and immunocytochemistry staining

Cardiomyocyte-depleted cardiac small cells were prepared as previously described with some modifications (Oh *et al*, 2003; Pfister *et al*, 2005). The minced heart tissue was digested with 0.1% collagenase B (Roche Molecular Biochemicals), 2.4 U/ml dispase II (Roche Molecular Biochemicals) and 2.5 mM CaCl_2_ at 37°C for 30 min and then filtered through a 40-μm filter. For isolation of cardiac Sca-1^+^ cells, the cardiac small cells were incubated with the Phycoerythrin (PE)-conjugated Sca-1^+^ antibodies (BD Bioscience) at 4°C for 30 minutes. The PE-labeled Sca-1^+^ cells were then sorted by the magnetic particles against PE (BD Biosciences). Respective isotype controls (BD Biosciences or GeneTex) were used as negative controls. Flow cytometry was performed using the FACSCanto™ (BD). The FACSDiva™ (BD) and FlowJo software was used for data analysis. For cell culture, 3×10^5^ cells were plated per well in a 6-well dish coated with 200 μg/ml fibronectin (Millipore). The cells were cultured in Iscove's Modified Dulbecco's Medium (IMDM) (Invitrogen) supplemented with 10% FBS and penicillin/streptomycin at 37°C. The culture medium was changed 3 days after plating and the cells were treated with PGE_2_ (10 μM) for another 3 days. On day 10, immunocytochemistry (ICC) staining was performed. For ICC staining, the cells were fixed in 2% paraformaldehyde and blocked in 1% BSA. The cells were stained with the cTnT (1:100, DSHB) overnight at 4°C and membrane dye WGA (5 μg/ml, Invitrogen) at room temperature for 10 minutes.

### Flow cytometric analysis of macrophages

Cardiomyocyte-depleted cardiac small cells were prepared as aforementioned method and 2 × 10^6^ of cell were stained at 4°C in a total volume of 100 μl with the following antibodies: CD45-PE-Cy7 (1:100; BD), CD11b-PerCP-Cy5.5 (1:100; BD), F4/80-APC (1:20; AbD Serotec), Gr-1-FITC (1:100; eBioscience), CD206-PE (1:100; AbD Serotec). Analysis was performed using FACSCanto™ (BD) and SH800 (SONY). The FACSDiva™ (BD) and FlowJo software was used for data processing.

**The paper explained**

**Problem**

It is known that the mammalian heart is capable of renewing cardiomyocytes and several cardiac stem/progenitor cells have been identified. However, little is known about the signal that stimulates endogenous stem cells to undergo differentiation, thus hindering the development of pharmacological interventions for treating heart injury.

**Results**

With an adult cardiomyocyte fate-mapping approach, we show that endogenous stem/progenitor cell-driven cardiac repair is initiated within 7 days and saturated on day 10 post-injury. The key to this stem/progenitor cell-dependent response is the COX-2-depdent inflammatory response in which PGE_2_ plays a critical role. We identify that cardiac Sca-1^+^ cells are the population most responsive to PGE_2_ stimulus and that EP2 signaling axis plays an important role in modulating cardiac stem cell activities. Interestingly, the aged heart is unable to self-repair but PGE_2_ can restore this lost ability, possibly by lowering the expression of the aging marker gene, *TGF-β1*.

**Impact**

Here we identify the critical time period and the essential signal that activates the endogenous stem/progenitor cells for cardiac repair. Furthermore, we discover that PGE_2_ can modulate stem cell activities. Following administration of PGE_2_, the ability of stem/progenitor cells to replenish lost cardiomyocytes is improved in young mice and is restored in aged mice. Importantly, delivery of PGE_2_ can be achieved via systemic injection, thus avoiding an invasive surgical approach. These results not only open a new avenue in the molecular mechanism controlling the post-MI inflammatory microenvironments but also pave the way for spontaneous cardiac repair and regeneration in the future.

### Data analysis

The results were statistically analyzed using either one-way ANOVA or *t*-tests. A result was considered to be statistically significant if the *P* < 0.05.
