# Supplementary material for: Prostaglandin E2 promotes post-infarction cardiomyocyte replenishment by endogenous stem cells
Source: EMBO Mol Med. 2014 Jan 21;6(4):496–503. doi: 10.1002/emmm.201303687 (PMC3992076; doi:10.1002/emmm.201303687)
Supplement: Supplementary file 1 [file emmm0006-0496-sd1.pdf]

EMBO Molecular Medicine

Prostaglandin E2 promotes post-infarction cardiomyocyte replenishment by endogenous stem cells

Ying-Chang Hsueh, Jasmine M.F. Wu, Chun-Keung Yu, Kenneth K. Wu, and Patrick C. H. Hsieh

Corresponding author: Patrick Hsieh, National Cheng Kung University

Review timeline:

Submission date:	27 March 2013
Editorial Decision:	07 May 2013
Appeal:	09 May 103
Editorial Decision:	13 May 2013
Revision received:	30 July 2013
Editorial Decision:	26 August 2013
Resubmission:	18 November 2013
Editorial Decision:	12 December 2013
Accepted:	16 December 2013

Transaction Report:

(Note: With the exception of the correction of typographical or spelling errors that could be a source of ambiguity, letters and reports are not edited. The original formatting of letters and referee reports may not be reflected in this compilation.)

Editor: Céline Carret

1st Editorial Decision 07 May 2013

Thank you for the submission of your manuscript "Prostaglandin E2 promotes post-infarction cardiomyocyte replenishment by endogenous stem cells". We have now heard back from the three referees whom we asked to evaluate your manuscript.

As you will see, the referees acknowledge the potential interest of the study, however they also raise very serious issues about the preliminary nature of the data in that the results do not fully support the conclusions drawn. As such, I am afraid that these criticisms are significant enough as to preclude publication of the manuscript in EMBO Molecular Medicine.

I am sorry that I could not bring better news this time and hope that the referee comments are helpful in your continued work in this area.

***** Reviewer's comments *****

Referee #1:

Comments to authors

This paper addresses whether and how the GPCR ligand PGE affects cardiomyocyte turnover and renewal and if new cardiomyocytes are generated, what their origin might be. The approach that is taken is population-based genetic pulse-chase. This experimental approach was introduced almost 10 years ago by the Melton group (Dor Y, ... Melton D, Nature), to address the question of cellular

© EMBO

turnover of pancreatic beta cells. Their conclusions were that pancreatic beta cells that are generated in adult mice are not derived from a stem cell compartment. The Melton-model has now been convincingly rejected by multiple focused lineage tracing experiments. These developments in the pancreas field cast doubt on the level of definitiveness that these pulse-chase experiments, which are based on dilution of a permanent label placed in differentiated cells, can provide.
In the field of cardiomyocyte biology, this general approach was emulated by using the alpha-MCH-MerCreMer mice for genetic pulse-chase experiments. Over the past years, this has resulted in somewhat contradictory results. The original report (Hsieh P, ... Lee RT, Nat Med 2007) suggested that after myocardial infarction in mice, new cardiomyocytes are generated from undifferentiated stem- or progenitor cells. However, a follow-up report from the same group, using a slightly modified technique, but the same genetic method to place the permanent genetic label (Senyo, ..., Lee RT, Nature 2012), indicated that new cardiomyocytes generated after MI originate from pre-existing differentiated cardiomyocytes. These somewhat contradictory results are mentioned here to point out that the experimental approach that is taken in the present paper does not measure up to the high level of definitiveness that readers of EMBO Molecular Medicine expect. The key results that are shown in this paper would have to be re-done using more definitive approaches.

Referee #2:

In this manuscript the authors use mouse genetics to fate map cardiomyocytes generated de novo after myocardial infarction in adult hearts. They conclude that there is a critical time period for cardiomyocyte replenishment after injury, which disappears with age or with anti-inflammatory drugs or Cox-2 inhibitors. Correspondingly they claim that administration of the COX-2 product prostaglandin E2 (PGE2) enhances cardiomyocyte replenishment in young mice and attenuates TGF- 1 signaling in aged mice to improve recovery.
While the topic of the research is very interesting and timely, and would be appropriate for EMBO Molecular Medicine If the work was done properly, the authors' conclusions are not adequately supported, due to insufficient or inappropriate experiments. As such, their research findings are too preliminary and the conclusions unsubstantiated to warrant publication in EMBO Molecular Medicine.

Specific comments for the authors' consideration:

1. Unsupported conclusions/concerns:

a. (Pg 5, para 2) Contrary to the authors' suggestions, there is no data supplied 'implying' that an early COX2/PGE2 signalling is required for induction of cardiomyocyte replenishment. If Celecoxib had different outcomes when administered early vs late, this may support their conclusion.
b. (Pg 6 para 1) There is no evidence that PGE2 acts directly on progenitor/stem cells.
c. (Pg 5 para 3) Despite the authors' suggestions, the underlying mechanism of PGE2's salutary effect is not determined. Does it operate on macrophages, progenitors, HSCs, or other cells? The authors cite work from North et al (2007) and Hoggatt et al (2009) indicating that PGE2 effects HSC mobilisation. HSC mobilisation has been previously indicated to improve cardiac function after injury. The authors do not pursue the mechanistic basis of their observations.
d. The quantification of M1 and M2 macrophages conducted does not support any of the authors' conclusions.
i. CD11c is not an M1 macrophage marker.
ii. CD11b is a pan myeloid marker within leukocytes and not exclusive to macrophages.
iii. The approach used is not appropriate. To adequately address this question, a flow cytometry experiment is necessary. This can be easily achieved using CD45, CD11b, F4/80, Ly6c (Gr-1) and CD206 antibodies. This is a very simple experiment and is essential to conclusively determine if macrophage polarization is affected by PGE2.
e. (Pg 8 para 2) It cannot be concluded that PGE2 is enhancing cardiopoiesis of injured aged hearts by modulating TGF 1 activity.
f. All inhibitor studies are non-tissue specific. The effect of these reagents on systemic elements, such as the bone marrow/HSC niches, spleen etc cannot be delineated. This must be discussed in the discussion along with the important effect of PGE2 in HSC mobilisation, which is salutary for the

© EMBO

injured heart.

2. General queries/comments:

a. Fig 1b: How many days after injury were the images taken?
b. Fig 2a. Does Celecoxib have different outcomes when administered early vs late (similar to the indomethacin experiment)?
c. Does PGE2 administration rescue Celecoxib treated mice, similar to Indomethacin treated animals? No data of PGE2 + Celecoxib treatment.
d. To confirm PGE2 is having a effect by directly acting upon progenitors or macrophages, a conditional EP2 and/or EP4 receptor ablation (for example in macrophages) experiment is necessary. Without this experiment, it is not possible to conclude how PGE2 is having a salutary effect after injury.
e. Fig S2: Where in the infarcted heart has the image been taken? The image of the CD11b staining could not be of the cardiac lesion after MI 3 days after injury. At this location, at this time point, it is extremely difficult to even see individual CD11b+ cells due to the massive influx of myeloid cells.
f. Why there is an improvement of heart function 2 months after injury, but not 1 month, should be addressed.
g. What is the age of the 'young' mice?
h. Acknowledgments: The supporting institutes are from which countries?

Referee #3 (Comments on Novelty/Model System):

The authors use a tracing model that has to date published several times. Recently, this method has also been used in a Nature article combined with another method and come to the conclusion that it is very difficult to explain the loss of GFP-positive cells by progenitor differentiation as the additional used method suggest that the progenitor cells should in this case be unable to proliferate. This appears rather unlikely.

Referee #3 (Remarks):

The authors address an important question in the field of cardiac regeneration. Since several years groups work worldwide on the identification of strategies to regenerate the mammalian heart. Even though the mammalian heart appears unable to regenerate after an injury there are reports of several endogenous stem cells in the heart and the observation that adult mammalian cardiomyocytes retain the ability to proliferate.
The biggest controversy is possibly the fact that some reports suggest that the human heart renews itself every couple of years, despite the fact it fails to recover from injury. Here, the authors describe that PGE2 treatment enhances stem cell-mediated replenishment of adult cardiomyocytes after myocardial infarction. Moreover, PGE2 can reactivate this mechanism via regulation of TGFbeta signaling in aged mice.

1) First of all this manuscript does not reflect/discuss the controversial character of this study and the conflicting results to other studies. For example, there is to this reviewers knowledge no study available that unambiguously demonstrates that stem cells differentiate into cardiomyocytes in vivo. In addition, the recent data by Senyo suggest that the replenishment of cardiomyocytes is difficult to explain by stem cell differentiation. At least, the data suggest that existing stem/progenitor cells do not proliferate. As these cells are difficult to find it is hard to explain how they generate 10% new cardiomyocytes. This should at least be discussed as well as possible problems of the assay.

2) How many fields and cells have the authors counted? From the sections in the figure one can see that the authors selected sections with cardiomyocytes sectioned vertical as well as horizontal. Also staining intensity is very different?
3) Overall, all differences shown are significant but minor. Also the improvement of the ejection fraction is with around 5% much lower than many other published strategies. Thus, the importance and future impact of PGE2 remains unclear.

© EMBO

4) Ejection fraction after 1 month is not improved by PGE2 but after 2 months. What is the possible mechanism if the authors have shown in Fig. 1 that the window of progenitor-cell mediated cardiomyocyte replenishment is from post MI day 7 to 10? Does this not indicate that the PGE2-mediated effect is stem-cell independent? And how do the increased IL10 levels at day 3 post MI fit?

5) Is the ejection fraction for PGE2 treated animals at 2 month significantly higher than for MI or PGE2-treated animals at 1 month?

6) The authors have shown interesting and intriguing data in vivo. However, those data are often difficult to interpret and as mentioned above it is clear if data are only correlated or are depended on each other. Thus, it is mandatory, that the authors show in cell-based assays that PGE2 has a significant effect on progenitor cell differentiation.

7) According to the authors data the progenitor cells differentiate within 7 to 10 days to adult cardiomyocytes. Is the reviewer's interpretation correct? Or can progenitor cell-derived cardiomyocytes distinguished? Considering development, in vitro stem cell differentiation assays and neonatal cardiomyocyte differentiation assays in 3D this is extremely fast.

Appeal 09 May 103

Thank you very much for reviewing our manuscript entitled "Prostaglandin E2 promotes post-infarction cardiomyocyte replenishment by endogenous stem cells" (manuscript ID: EMM-2013-02812). After careful examination of the Reviewers' comments, we found that all questions and concerns raised could be answered by discussion or additional experiments. Therefore, we are writing to request for a chance of revision.

We have examined extensively the ability for stem/progenitor cells to differentiate into cardiomyocytes and the role of PGE2-EP2 signaling during this differentiation through in vivo experiments with Sca-1::GFP transgenic mice and PGE2 receptor-deficient EP2 knockout mice. We did not include these data in the submitted manuscript because we consider these findings beyond the scope of the current study. However, in order to answer the concerns raised by the Reviewers, we would like to incorporate these in vivo results into the original manuscript to make the paper more comprehensive.

Our animal studies demonstrated that Sca-1+ cells were responsive to injury because there was an increase of cardiac small cells in Sca-1::GFP mice after myocardial infarction (Figure 1, enclosed). Furthermore, we found that the cardiomyocyte differentiation ability of Sca-1+ cells derived from EP2 knockout mice was lost (Figure 2, enclosed). We also proved that PGE2 enhances the probability for stem/progenitor cells to differentiate into cardiomyocytes via in vitro culture system. The observation that stem/progenitor cells are able to replenish the lost cardiomyocytes in vivo is supported by a recent study (Malliaras et al., 2013) in EMBO Molecular Medicine. Moreover, using genetic fate-mapping, Smart et al. also demonstrated that Wt1+ progenitor cells could replace the lost cardiomyocytes within 14 days post-injury (Nature, 2011), which is consistent with our findings.

On the other hand, although our findings and those reported by Senyo's study appear to be contradicting (Nature, 2013), the contribution of progenitor cell-derived cardiomyocytes still cannot be overlooked. In the same issue, Palacios and Schneider have pointed out in a commentary article in EMBO Molecular Medicine that the sample number, 35 15N+ cardiomyocytes out of 4,000 cells examined, in Senyo's study is too small, suggesting potential controversy of their finding. Comparing to the study reported by Melton's group where the labeling efficiency was less than 60% (Nature, 2004), the fate-mapping system used in the field of cardiomyocyte biology can achieve more than 80% of labeling efficiency (Qian et al., Nature, 2012; Senyo et al., Nature, 2013; Malliaras et al., EMBO Mole. Med., 2013). We along with other groups have observed that myocardial infarction results in a 20% increase of un-labeled cardiomyocytes, indicating reproducibility and reliability of the cardiomyoc yte fate-mapping system. To our knowledge, this is

© EMBO

to date the most suitable system to examine the contribution of endogenous stem/progenitor cells to cardiomyocyte replenishment after injury. With different experimental approaches, we and Malliaras et al. both proved the significance of stem/progenitor cells in cardiomyocyte replacement post-injury. Nevertheless, the extent to which the stem/progenitor cells contribute to cardiomyocyte replenishment after injury and the time point at which it occurs remain controversial and require further investigation. How to augment the activity of endogenous cardiac regeneration has not been demonstrated until our study. Therefore, we strongly believe that our study can provide resolution to these unexplored topics and can further aid the movement of cardiac therapies.

By this we do not imply any lack of esteem for you or the journal. We simply want to express our full capability in addressing all the concerns raised by the Reviewers. We sincerely hope that a chance for revision can be granted to us. Thank you very much for your consideration. I look forward to hearing your decision.

References:

Dor Y, Brown J, Martinez OI, Melton DA (2004) Adult pancreatic -cells are formed by self-duplication rather than stem-cell differentiation. Nature 429: 41-46.
Malliaras K, Zhang Y, Seinfeld J, Galang G, Tseliou E, Cheng K, Sun B, Aminzadeh M, Marb·n E (2013) Cardiomyocyte proliferation and progenitor cell recruitment underlie therapeutic regeneration after myocardial infarction in the adult mouse heart. EMBO Molecular Medicine 5: 191-209.
Palacios JA, Schneider MD (2013) Heart to heart: grafting cardiosphere-derived cells augments cardiac self-repair by both myocytes and stem cells. EMBO Molecular Medicine 5: 177-179.
Qian L, Huang Y, Spencer CI, Foley A, Vedantham V, Liu L, Conway SJ, Fu J-d, Srivastava D (2012) In vivo reprogramming of murine cardiac fibroblasts into induced cardiomyocytes. Nature 485: 593-598.
Senyo SE, Steinhauser ML, Pizzimenti CL, Yang VK, Cai L, Wang M, Wu T-D, Guerquin-Kern J-L, Lechene CP, Lee RT (2013) Mammalian heart renewal by pre-existing cardiomyocytes. Nature 493: 433-436.
Smart N, Bollini S, Dube KN, Vieira JM, Zhou B, Davidson S, Yellon D, Riegler J, Price AN, Lythgoe MF et al (2011) De novo cardiomyocytes from within the activated adult heart after injury. Nature 474: 640-644.

2nd Editorial Decision 13 May 2013

Thank you for your letter asking us to reconsider our decision on your manuscript. I have now consulted with the other members of our editorial team, including our chief editor. In light of the new data you are ready to add and with the understanding that all comments should be experimentally addressed when required, we feel that we can offer to revise the manuscript.

Please note that that it is our journal's policy to allow only a single round of revision, and that acceptance or rejection of the manuscript will therefore depend on the completeness of your response and the satisfaction of the referees with it.

I look forward to seeing a revised form of your manuscript as soon as possible.

1st Revision - authors' response 30 July 2013

© EMBO

Referee #1:

This paper addresses whether and how the GPCR ligand PGE affects cardiomyocyte turnover and renewal and if new cardiomyocytes are generated, what their origin might be. The approach that is taken is population-based genetic pulse-chase. This experimental approach was introduced almost 10 years ago by the Melton group (Dor Y, ... Melton D, Nature), to address the question of cellular turnover of pancreatic beta cells. Their conclusions were that pancreatic beta cells that are generated in adult mice are not derived from a stem cell compartment. The Melton-model has now been convincingly rejected by multiple focused lineage tracing experiments. These developments in the pancreas field cast doubt on the level of definitiveness that these pulse-chase experiments, which are based on dilution of a permanent label placed in differentiated cells, can provide. In the field of cardiomyocyte biology, this general approach was emulated by using the alpha-MCH-MerCreMer mice for genetic pulse-chase experiments. Over the past years, this has resulted in somewhat contradictory results. The original report (Hsieh P, ... Lee RT, Nat Med 2007) suggested that after myocardial infarction in mice, new cardiomyocytes are generated from undifferentiated stem- or progenitor cells. However, a follow-up report from the same group, using a slightly modified technique, but the same genetic method to place the permanent genetic label (Senyo, ..., Lee RT, Nature 2012), indicated that new cardiomyocytes generated after MI originate from pre-existing differentiated cardiomyocytes. These somewhat contradictory results are mentioned here to point out that the experimental approach that is taken in the present paper does not measure up to the high level of definitiveness that readers of EMBO Molecular Medicine expect. The key results that are shown in this paper would have to be re-done using more definitive approaches.

We thank the Reviewer for raising the concern. Regarding the adult cell fate-mapping system, we would like to clarify that the reporter mouse used by Melton's group is **Z/AP**, which is different from the **Z/EG** transgenic mouse used in our study. Following Cre-mediated excision of the STOP sequence in the Z/AP mice, pan expression of AML1-ETO fusion protein and placental alkaline phosphosphatase (ALPP or PLAP) can lead to cancer development. In contrast, Z/EG mice do not have such problem. Our experience with Z/AP mice suggests the labeling efficiency of cardiomyocytes in these mice is relatively low (less than 30%), similar to that in the Melton's paper for β-cells. Such labeling efficiency is inadequate for adult cell fate-mapping.

In our study, the α-MHC:MerCreMer were crossbred with the Z/EG mice. Consistent with previous studies, 14-day tamoxifen or 4-OH-tamoxifen injection resulted in ~83% labeling efficiency of cardiomyocytes in double transgenic MerCreMer/ZEG mice. Furthermore, we and others have observed that β-Gal$^+$ cardiomyocytes increased by ~15% at the peri-infarct after myocardial infarction (MI), suggesting reproducibility of the results and reliability of the fate-mapping system (Loffredo et al, 2011; Malliaras et al, 2013; Qian et al, 2012; Senyo et al, 2013).

The study reported by Senyo's paper suggests that the pre-existing cardiomyocytes are the major population to replenish lost cells (Senyo et al, 2013). Despite the ^{15}N labeling system was used to evaluate the contribution of pre-existing cardiomyocytes in the injured heart, a dilution in GFP$^+$ cell pool after MI could not be explained. Therefore, the participation of endogenous stem/progenitor cells in cardiomyocyte regeneration cannot be excluded. To provide further evidence that the M/Z system is a suitable approach to

examine stem/progenitor cell-dependent cardiac repair, *in vitro* and *in vivo* experiments were performed to investigate the role of PGE_2 in cardiomyocyte regeneration.

To identify the stem cell population responsive to the PGE_2 treatment, we performed quantitative RT-PCR to analyze the expression of several stem/progenitor cell marker genes. Of all the genes analyzed, expression of *Sca-1* (Oh et al, 2003; Sturzu & Wu, 2011) was most significantly enhanced by PGE_2 (Supporting Information Fig 4). Furthermore, administration of PGE_2 increased expression of *Nkx2.5* (Wu et al, 2006) in the Sca-1$^+$ cells isolated from the heart at day 3 post-MI (Supporting Information Fig 6). Because tamoxifen injection in M/Z mice leads to the conversion of β-Gal to GFP in cardiomyocytes, we thought to take this advantage to examine the cardiomyogenic differentiation ability of cardiac Sca-1$^+$ cells. The tamoxifen injection was given to the M/Z mice after MI surgery, and therefore, only α-MHC$^+$ cells would express GFP. This experiment allowed us to determine whether Sca-1$^+$ cells possess the ability to differentiate into α-MHC$^+$ cells. Following MI surgery and tamoxifen injection for 3 days, Sca-1$^+$/GFP$^+$ cells could be detected (Supplementary Information Fig 7A). The percentage of double positive cells further increased upon PGE_2 treatment (Supplementary Information Fig 7B and C). In addition, Sca-1$^+$/α-MHC$^+$ cells were not observed before tamoxifen labeling and the population did not arise from cardiomyocyte de-differentiation (Hsieh et al, 2007; Senyo et al, 2013; Supplementary Information Fig 8). These results reveal the potential contribution of cardiac Sca-1$^+$ stem/progenitor cells to cardiomyocyte replenishment after MI.

Next, we investigated the effect of PGE_2 on cardiomyogenic differentiation of cardiac Sca-1$^+$ cells. Following isolation of Sca-1$^+$ cells and cardiomyocyte-depleted small cells from young mice, the cells were cultured on fibronectin-coated plates for 3 days, as it took at least 3 days for the cells to attach to the plate (Oh et al, 2003).Then, cells were treated with PGE_2 for another 3 days and their cardiomyocyte differentiation potential was examined at day 10 (Goessling et al, 2009). Compared to the vehicle alone group, we observed *cTnT* (Hsieh et al, 2007) expression and immature sarcomeric structure in the Sca-1$^+$ cells treated with PGE_2 (Supporting Information Fig 12B and C). Surprisingly, mature sarcomeric organization was seen in the cardiomyocyte-depleted small cells after PGE_2 treatment (Supporting Information Fig 12A and B). The small cell fraction contains several types of differentiated cells and stem cells, including immune cells, fibroblasts, endothelial cells as well as Sca-1$^+$ cells and mesenchymal stem cells (Beigi et al, 2013; Degousee et al, 2008; Oh et al, 2003; Wong et al, 1998). Therefore, we suspect that PGE_2 not only directly acts on the Sca-1$^+$ stem/progenitor cells but also other cardiac small cells, thereby exerting an indirect effect on modulating cardiomyocyte differentiation. Nevertheless, further investigation is necessary to determine the underlying mechanism of PGE_2-promoted cardiomyocyte differentiation in the small cells.

To further dissect the role of PGE_2 in regulating cardiomyocyte differentiation, we first analyzed the expression of the PGE_2 receptors *EP1-EP4* (Hoggatt et al, 2009) in hearts subjected to MI surgery with or without Indomethacin or PGE_2 treatment. We observed that *EP*2 expression was significantly higher than *EP1*, *EP3* and *EP4* expression after MI (Supporting Information Fig 9). Furthermore, *EP2* expression was also induced in cardiac Sca-1$^+$ cells after MI (Supporting Information Fig 10). These findings imply that EP2 plays a role in cardiomyocyte regeneration. To confirm this, an independent experiment using EP2 knockout (EP2$^{-/-}$; Kennedy et al, 1999) mice was performed (Supporting Information Fig 11A). We isolated Sca-1$^+$ cells from the EP2$^{-/-}$ mice and injected them into infarcted M/Z hearts (Loffredo et al, 2011). In contrast to the hearts receiving wild-type cardiac Sca-1$^+$ cells (EP2$^{+/+}$), injection of EP2$^{-/-}$ Sca-1$^+$ cells did not

change the proportion of GFP^+ or β-Gal^+ cardiomyocytes (Figure 2F, Supporting Information Fig 11B). These findings further support the notion that cardiac Sca-1^+ stem/progenitor cells may be a source of cardiomyocyte replenishment and that the COX-2/PGE_2/EP2 pathway plays a key role in modulating this process. The new *in vitro* and *in vivo* data have been incorporated in the revised manuscript.

Results in Senyo's study (Senyo et al, 2013) suggest that the main cell source to replenish cardiomyocytes after heart injury is the pre-existing cardiomyocytes. However, the contribution of stem/progenitor cells cannot be excluded in their study. It is also possible that the stem/progenitor cell-derived cardiomyocytes generated at an early time point may be labeled following long-term isotope tracing. A commentary article reported by Palacios J.A. & Schneider M.D. in EMBO Molecular Medicine has pointed out that the number of cells, 35 $^{15}N^+$ cardiomyocytes out of 4,000 cells examined, reported by Senyo et al. is too small, suggesting potential controversy of their finding. It may not be objective to evaluate the proliferation ability of different cell populations based solely on small sample size (Palacios & Schneider, 2013). Malliaras *et al*. used flow cytometry for global quantification of cardiomyocyte proliferation rate, as determined by BrdU incorporation, in MI heart with the M/Z system (Malliaras et al, 2013). They provided evidence that stem/progenitor cell-derived GFP^- cardiomyocytes have better proliferation ability than the residing GFP^+ cardiomyocytes after MI, which is in the contrary to the results reported by Senyo's study. Furthermore, the number of GFP^- cardiomyocytes entering cell cycle could be greatly amplified by cell transplantation therapy. Therefore, it remains inconclusive whether the pre-existing cardiomyocytes are the major contributor for cardiomyocyte replenishment and further examinations are necessary. On the other hand, both our and Malliaras *et al*.'s results support the notion that targeting the stem/progenitor cells for cardiac repair remains an important therapeutic approach (Malliaras et al, 2013).

Collectively, our results are not in conflict with Senyo's. Despite that the ^{15}N was used to label the pre-existing cardiomyocytes entering the cell cycle after MI, a 15% dilution in the GFP^+ cardiomyocyte pool could still be observed (Senyo et al, 2013). Furthermore, contribution of stem/progenitor cell-derived cardiomyocytes to the GFP^+ cell pool dilution was not excluded based on their experimental design. Senyo's study strengthens the necessity of stem/progenitor cell research for cardiac repair because only 0.002% (16/7063) of pre-existing cardiomyocytes complete cell division while the GFP^+ cells could be diluted by 15%. We have incorporated the new results and discussion into the revised manuscript as the following.

References:

Degousee N, Fazel S, Angoulvant D, Stefanski E, Pawelzik S-C, Korotkova M, Arab S, Liu P, Lindsay TF, Zhuo S et al (2008) Microsomal Prostaglandin E2 Synthase-1 Deletion Leads to Adverse Left Ventricular Remodeling After Myocardial Infarction. Circulation 117: 1701-1710

Goessling W, North TE, Loewer S, Lord AM, Lee S, Stoick-Cooper CL, Weidinger G, Puder M, Daley GQ, Moon RT et al (2009) Genetic Interaction of PGE2 and Wnt Signaling Regulates Developmental Specification of Stem Cells and Regeneration. Cell 136: 1136-1147

Hoggatt J, Singh P, Sampath J, Pelus LM (2009) Prostaglandin E2 enhances hematopoietic stem cell homing, survival, and proliferation. Blood 113: 5444-5455

Hsieh PCH, Segers VFM, Davis ME, MacGillivray C, Gannon J, Molkentin JD, Robbins J, Lee RT (2007) Evidence from a genetic fate-mapping study that stem cells refresh adult mammalian cardiomyocytes after injury. Nat Med 13: 970-974

Kennedy CRJ, Zhang Y, Brandon S, Guan Y, Coffee K, Funk CD, Magnuson MA, Oates

JA, Breyer MD, Breyer RM (1999) Salt-sensitive hypertension and reduced fertility in mice lacking the prostaglandin EP2 receptor. Nat Med 5: 217-220
Loffredo Francesco S, Steinhauser Matthew L, Gannon J, Lee Richard T (2011) Bone Marrow-Derived Cell Therapy Stimulates Endogenous Cardiomyocyte Progenitors and Promotes Cardiac Repair. Cell Stem Cell 8: 389-398
Malliaras K, Zhang Y, Seinfeld J, Galang G, Tseliou E, Cheng K, Sun B, Aminzadeh M, Marbán E (2013) Cardiomyocyte proliferation and progenitor cell recruitment underlie therapeutic regeneration after myocardial infarction in the adult mouse heart. EMBO Mol Med 5: 191-209
Oh H, Bradfute SB, Gallardo TD, Nakamura T, Gaussin V, Mishina Y, Pocius J, Michael LH, Behringer RR, Garry DJ et al (2003) Cardiac progenitor cells from adult myocardium: Homing, differentiation, and fusion after infarction. Proc Natl Acad Sci USA 100: 12313-12318
Palacios JA, Schneider MD (2013) Heart to heart: grafting cardiosphere-derived cells augments cardiac self-repair by both myocytes and stem cells. EMBO Mol Med 5: 177-179
Qian L, Huang Y, Spencer CI, Foley A, Vedantham V, Liu L, Conway SJ, Fu J-d, Srivastava D (2012) In vivo reprogramming of murine cardiac fibroblasts into induced cardiomyocytes. Nature 485: 593-598
Senyo SE, Steinhauser ML, Pizzimenti CL, Yang VK, Cai L, Wang M, Wu T-D, Guerquin-Kern J-L, Lechene CP, Lee RT (2013) Mammalian heart renewal by pre-existing cardiomyocytes. Nature 493: 433-436
Sturzu AC, Wu SM (2011) Developmental and Regenerative Biology of Multipotent Cardiovascular Progenitor Cells. Circ Res 108: 353-364
Wong SCY, Fukuchi M, Melnyk P, Rodger I, Giaid A (1998) Induction of Cyclooxygenase-2 and Activation of Nuclear Factor-κB in Myocardium of Patients With Congestive Heart Failure. Circulation 98: 100-103
Wu SM, Fujiwara Y, Cibulsky SM, Clapham DE, Lien C-l, Schultheiss TM, Orkin SH (2006) Developmental Origin of a Bipotential Myocardial and Smooth Muscle Cell Precursor in the Mammalian Heart. Cell 127: 1137-1150

Main Text:
Page 5–7
PGE_2 regulates cardiac Sca-1$^+$ cells

Among the identified markers, Sca-1 is commonly expressed in various cardiac stem/progenitor cell populations (Oh et al, 2003; Sturzu & Wu, 2011). We therefore sought to investigate the effect of PGE_2 on stem cell-mediated cardiomyocyte replenishment by examining Sca-1$^+$ cell activities. Quantitative RT-PCR revealed that only Sca-1 expression peaked on day 3 post-MI and this level was further increased at the same time point upon PGE_2 treatment but was repressed by indomethacin (Supporting Information Fig 4). Examination of cardiac transcription factor expression suggested that Nkx2.5 (Wu et al, 2006) had similar expression pattern to that of Sca-1 at the infarct zone and the remote area (Supporting Information Fig 5). Furthermore, PGE_2 also elevated the expression of Nkx2.5 in Sca-1$^+$ cells (Supporting Information Fig 6).

Because tamoxifen injection in M/Z mice leads to conversion of β-Gal to GFP in cardiomyocytes, we thought to take this advantage to examine cardiomyogenic differentiation ability of the cardiac Sca-1$^+$ cells. The tamoxifen injection was given to the M/Z mice after MI surgery, and therefore, only α-MHC$^+$ cells would express GFP (Supplementary Information Fig 7A). This experiment allowed us to determine whether Sca-1$^+$ cells possess the ability to differentiate into α-MHC$^+$ cells. Following MI surgery and tamoxifen injection for 3 days, Sca-1$^+$/GFP$^+$ cells could be detected. The percentage of double positive cells was further increased upon PGE_2 treatment (Supplementary Information Fig 7B and C). In addition, Sca-1$^+$/α-MHC$^+$ cells were not observed before tamoxifen labeling and they do not arise from cardiomyocyte

de-differentiation (Hsieh et al, 2007; Senyo et al., 2013) (Supplementary Information Fig 8). These results reveal the potential contribution of cardiac Sca-1+ stem/progenitor cells to cardiomyocyte replenishment after MI.

Following MI, M/Z system serves as a platform to assess the cardiomyocytes differentiated from endogenous stem/progenitor cells. To evaluate the cardiomyocyte differentiation ability of cardiac Sca-1^+ cells and the importance of PGE_2 pathway during this process, the cells were isolated from wild-type and $EP2^{-/-}$ mice (Kennedy et al, 1999) for intramyocardial injection after MI surgery (Loffredo et al, 2011; Supporting Information Fig 11A). The $EP2^{-/-}$ transgenic mouse was chosen due to the expression of this PGE_2 receptor was significantly induced in hearts after MI and in cardiac Sca-1^+ cells after PGE_2 treatment (Supporting Information Fig 9 and 10). Quantification of the GFP^+ and β-Gal^+ cardiomyocyte numbers revealed that injection of wild-type Sca-1^+ cells reduced both GFP^+ and β-Gal^+ cardiomyocyte numbers and that approximately 10% of the peri-infarct cardiomyocytes were GFP^- and β-Gal^-, suggesting cardiomyocyte differentiation of the injected cardiac Sca-1^+ cells. In contrast, we did not observe such change in the M/Z mice receiving injection of $EP2^{-/-}$ Sca-1^+ cells (Fig. 2F, Supporting Information Fig 11). Together these results indicate that the PGE_2/EP2 signaling may regulate the ability of cardiac Sca-1^+ cells to differentiate into cardiomyocytes. Results from in vitro culture also provided evidence that the expression of Nkx2.5 and cTnT was evidently improved in isolated cardiac small cells and Sca-1^+ cells by PGE_2 (Supporting Information Fig 12B and C). Surprisingly, mature sarcomeric structure, as determined by the expression of cTnT, were seen in the cardiomyocyte-depleted small cells after PGE_2 treatment (Supporting Information Fig 12A and B), suggesting the ability of PGE_2 to augment cardiomyocyte differentiation.

Discussion:
Page 10
On the basis of Senyo's findings, several commentary articles have pointed out that the contribution of stem/progenitor cells to cardiac repair may be negligible (Mummery & Lee, 2013). Despite the use of ^{15}N labeling system, one question that remains unsolved is the dilution of the GFP^+ cardiomyocyte pool in the M/Z mice after MI (Senyo et al, 2013). Results in our study and others have demonstrated that the number of cardiomyocytes replenished by endogenous stem/progenitor cells at the infarct border zone is greater than the number of cells derived from the dividing pre-existing cardiomyocytes. Here, the results also reveal that the ability of stem/progenitor cells to give rise to cardiomyocytes could be modulated, suggesting a potential therapeutic application of the endogenous stem/progenitor cells for cardiac repair.

Figures and Supporting Information Figures:

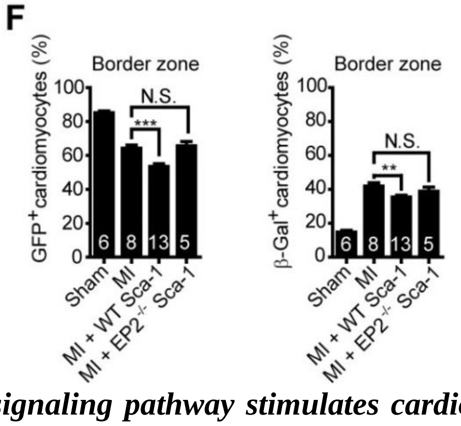

Figure 2. COX-2-dependent signaling pathway stimulates cardiomyocyte replenishment with endogenous stem/progenitor cells shortly after infarction.

F. Following DAB staining, the percentages of GFP^+ and β-Gal^+ cardiomyocytes at the border zone of the young heart with or without cell injection after MI were quantified and

*statistically analyzed. Sample size is indicated in the bar chart. **p< 0.01, ***p< 0.001; N.S., not significant. Data are presented as the mean ± s.e.m.*

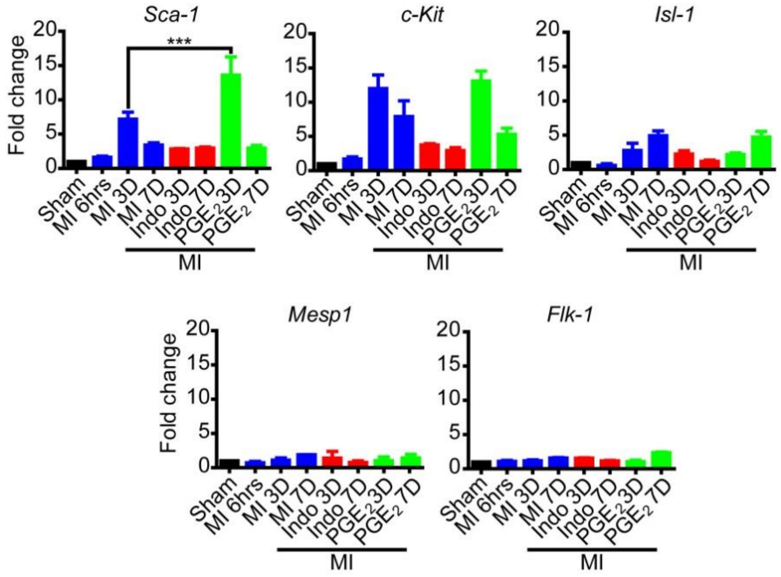

Supporting Information Fig 4. Gene expression of Sca-1 is the most responsive to PGE_2 treatment.

*Expression of the cardiac stem/progenitor marker genes in the infarcted region of injured hearts was analyzed by quantitative RT-PCR. The fold change is a relative quantification normalized to the sham control. ***p< 0.001. n ≥ 3. Data are presented as the mean ± s.e.m. Indo, Indomethacin; MI, myocardial infarction.*

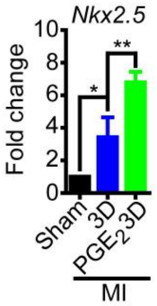

Supporting Information Fig 6. PGE_2 augments expression of Nkx2.5 in the Sca-1⁺ cells of injured heart.

*At day 3 post-surgery, the Sca-1⁺ cells from the heart treated with or without PGE_2 were isolated for quantitative RT-PCR analysis for Nkx2.5 expression. The fold change is a relative quantification normalized to the sham control. *p<0.05, **p<0.01. Data are presented as the mean ± s.e.m. MI, myocardial infarction.*

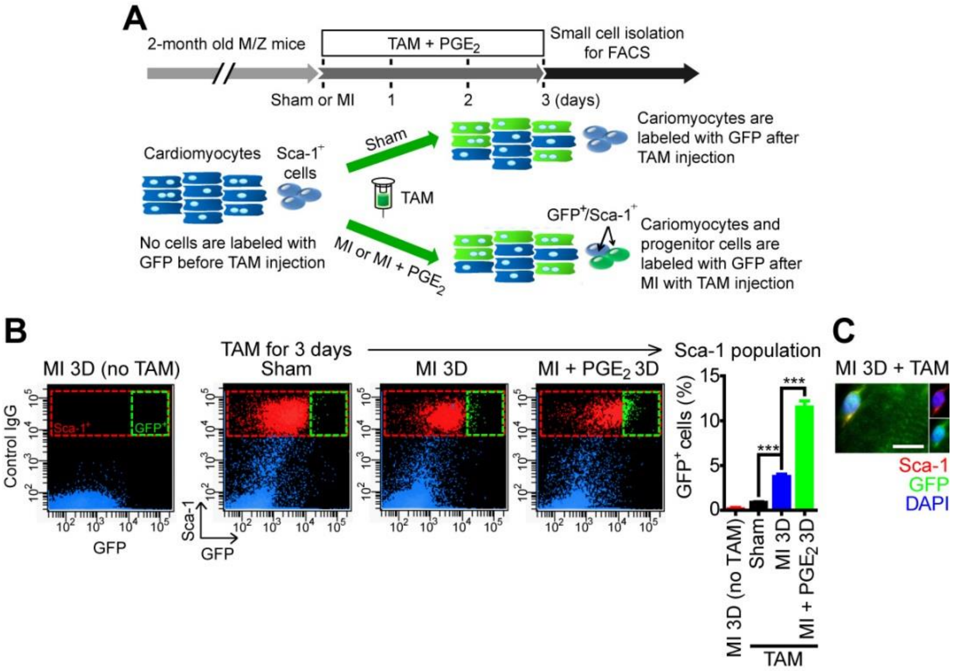

Supporting Information Fig 7. Examination of cardiomyogenic differentiation ability of cardiac Sca-1$^+$ cells after myocardial infarction.

A. *Schematic diagram depicting the experimental procedure. Following myocardial infarction (MI) surgery, the M/Z mice were injected with 80 µg/g tamoxifen (TAM) per day for 3 days with or without an additional PGE_2 treatment. The sham control was also treated with the same dosage of tamoxifen and PGE_2 simultaneously for 3 days. The cardiac small cells were isolated and subjected to flow cytometric analysis of Sca-1$^+$/GFP$^+$ cells at day 3 post-surgery.*
B. *The percentages of Sca-1$^+$/GFP$^+$ cells at day 3 post-MI were quantified by flow cytometry. Mice that did not receive tamoxifen injections after the MI surgery served as negative control (no TAM). The number of Sca-1$^+$/GFP$^+$ cells was also quantified. The data are presented as percentage calculated by dividing the number of double-positive cells by the total number of Sca-1$^+$ cells. ***$p<0.001$. Data are presented as the mean ± s.e.m. $n \geq 4$.*
C. *Prior to isolating the small cardiac cells for flow cytometry analysis, a small portion of the heart tissue was excised, fixed and immunostained. Scale bars, 10 µm.*

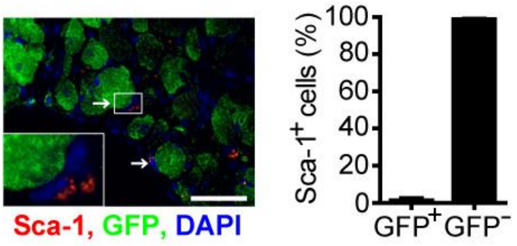

Supporting Information Fig 8. Sca-1$^+$/α-MHC$^+$ cells were not detected prior to tamoxifen labeling and these cells did not arise from cardiomyocyte de-differentiation.

Following 14 days of tamoxifen labeling, the animals were allowed to recover for 1 month prior to myocardial infarction (MI) surgery. At day 3 post-MI, the organ was collected for immunostaining analysis. Shown is a representative image of the Sca-1$^+$ cells and the original magnifications are as indicated. The Sca-1$^+$ cells with or without GFP$^+$ signal were quantified. Scale bars, 50 µm. n =3. Data are presented as the mean ± s.e.m.

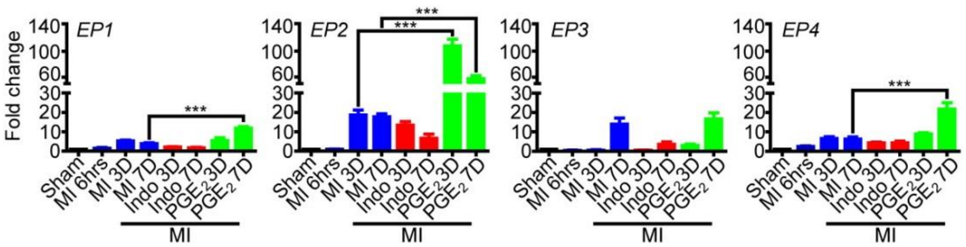

Supporting Information Fig 9. PGE_2 increases the expression of EP2 receptor after myocardial infarction.

*The expression of PGE_2 receptors, EP1, 2, 3 and 4, in response to different drug treatments at the infarct region of the injured heart was examined by quantitative RT-PCR. The fold change is a relative quantification normalized to the sham control. ***$p<0.001$. Data are presented as mean ± s.e.m. Indo, Indomethacin; MI, myocardial infarction.*

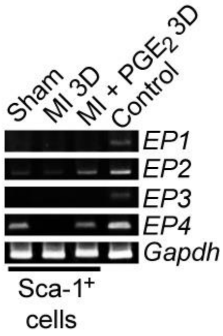

Supporting Information Fig 10. The expression of EP2 receptor increases in Sca-1$^+$ cells isolated from the infarcted heart after PGE_2 treatment.

Semi-quantitative PCR was performed to examine the expression of PGE_2 receptors, EP1, 2, 3 and 4, in cardiac Sca-1$^+$ cells isolated after myocardial infarction (MI). Un-sorted cardiomyocyte-depleted small cells from the sham group served as positive control.

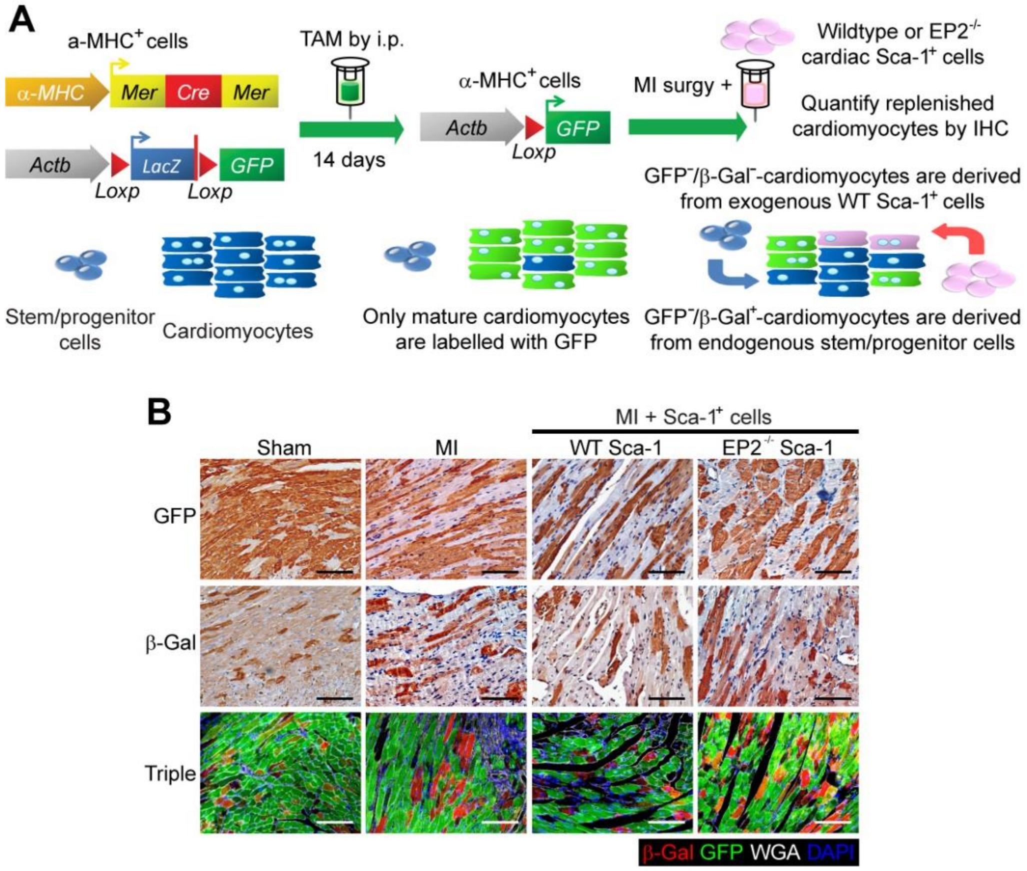

Supporting Information Fig 11. Quantification of the degree of cardiomyocyte replenishment after Sca-1^{+} cell injection in injured hearts.

A. *Schematic diagram depicting the experimental procedure. Following 14 days of tamoxifen injection, the M/Z mice were injected intramyocardially with wild-type or EP2 knockout (EP2$^{-/-}$) cardiac Sca-1^{+} cells after myocardial infarction (MI). The hearts were harvested at day 14 post-MI for examination.*
B. *At day 14 post-infarction, the hearts were harvested for DAB and immunofluorescence triple staining to examine the GFP^{+} or β-Gal^{+} cardiomyocytes. Shown are representative images from each group. Scale bars, 100 μm.*

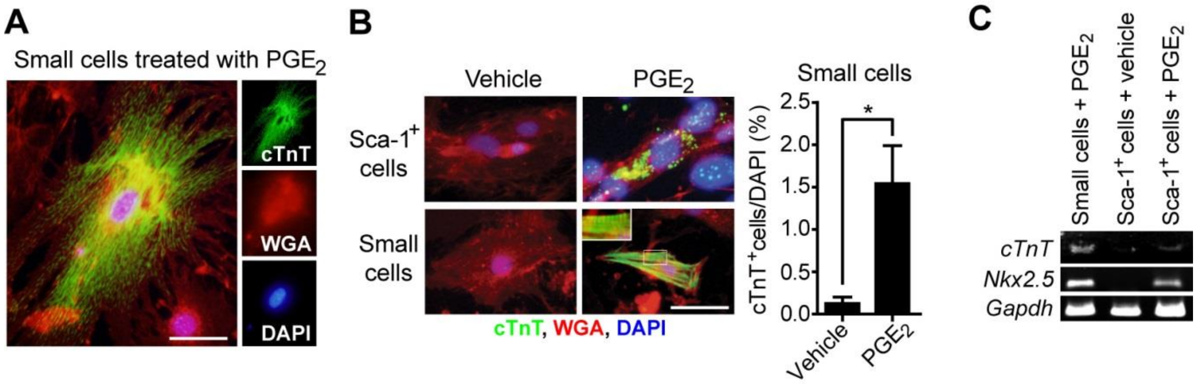

Supporting Information Fig 12. In vitro differentiation ability of isolated cardiac cells into cardiomyocytes.

A. *The cardiomyocyte-depleted small cells were allowed to attach for 3 days followed by PGE$_2$ treatment (10 μM) for another 3 days. Immunocytochemistry was performed to examine cardiomyocyte differentiation, as determined by the expression of the cardiomyocyte marker cardiac troponin T (cTnT) at day 10. The membrane and nucleus were stained with the membrane dye, WGA, and DAPI, respectively. Scale bar, 50 μm.*

B. *Under the same culture condition, the cardiac Sca-1$^+$ cells and small cells subjected to vehicle or PGE_2 treatment were stained with cTnT for sarcomeric structure analysis. The percentage of small cells with mature sarcomeric structures following vehicle or PGE_2 treatment was quantified. Scale bar, 50 μm. *$p < 0.05$. Data are presented as mean ± s.e.m.*

C. *Following the same culture procedure, the effect of PGE_2 on the expression of cardiac marker genes, Nkx2.5 and cTnT, in Sca-1$^+$ cells was analyzed by semi-quantitative PCR. Small cells treated with PGE_2 serve as positive control.*

Referee #2:

1. Unsupported conclusions/concerns:
a. (Pg 5, para 2) Contrary to the authors' suggestions, there is no data supplied 'implying' that an early COX2/PGE2 signalling is required for induction of cardiomyocyte replenishment. If Celecoxib had different outcomes when administered early vs late, this may support their conclusion.

We thank the Reviewer for pointing out this concern and providing helpful suggestion. To address this question, the mice were administrated with COX-2-specific inhibitor Celecoxib within 5 days or later at 9 days after surgical MI. Then, the mice were sacrificed 14 days after surgery for organ collection and analysis. In consistent with the Indomethacin treatment results, administration of Celecoxib within 5 days post-MI effectively blocked cardiomyocyte replenishment at the infarct border zone. Celecoxib did not, however, exert any inhibitory effect when it was give more than 5 days after heart injury. To address the Reviewer's **Question c (general comments)**, we conducted an experiment to evaluate the effect of PGE_2 on Celecoxib-dependent attenuation of cardiomyocyte repopulation. We discovered that PGE_2 could rescue the inhibitory effect of Celecoxib compared to Celecoxib treatment alone at an early time point, 5 days (Supporting Information Fig 3).

Collectively, we believe that our new data further strengthen the importance of early COX-2/PGE_2 signaling pathway for cardiomyocyte replenishment. The results have been incorporated into the revised manuscript.

Main text:
Page 5
Cardiomyocyte replenishment was also abolished upon treatment of Celecoxib, a selective COX-2 inhibitor (Lyons et al, 2011), for 14 days or within 5 days post-MI (Fig 2A and B and Supporting Information Fig 3).

Supporting Information Figure:

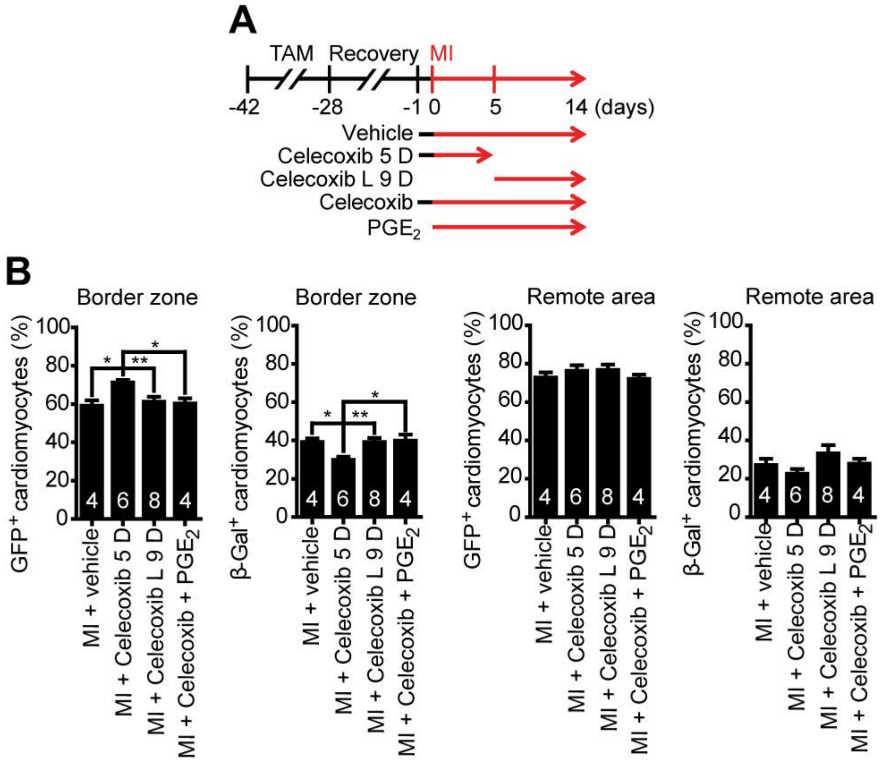

Supporting Information Fig 3. Early COX-2 signalling pathway is necessary for cardiomyocyte regeneration.

A. *Experimental paradigm of drug treatment on the MerCreMer/ZEG mice. Celecoxib was continuously administered 1 day before surgery until day 5 (Celecoxib 5 D) after MI or from day 5 to day 14 post-MI (Celecoxib L 9 D). Another group of animals was administrated with Celecoxib and PGE_2 simultaneously for 14 days. The hearts from drug-treated mice isolated at day 14 post-infarction were stained for GFP or β-Gal.*
B. *GFP^+ and β-Gal^+ cells at the border zone or remote area were quantified and statistically analyzed. *$p<0.05$, **$p<0.01$. Data are presented as the mean ± s.e.m. Sample size is indicated in the bar chart. MI, myocardial infarction.*

b. (Pg 6 para 1) There is no evidence that PGE2 acts directly on progenitor/stem cells.

We thank the Reviewer for raising this concern. To identify the stem cell population responding to the PGE_2 treatment, we performed quantitative RT-PCR to analyze the expression of several stem cell marker genes (Sturzu & Wu, 2011). Of all of the genes analyzed, expression of *Sca-1* (Oh et al , 2003) was the most significantly enhanced by PGE_2 (Supporting Information Fig 4). Following isolation of Sca-1^+ cells and cardiomyocyte-depleted small cells from young mice, the cells were cultured on fibronectin-coated plates for 3 days, as it took at least three days for the cells to attach (Oh et al, 2003). After attachment, cells were treated with PGE_2 for another 3 days and their cardiomyocyte differentiation potential was examined at day 10 (Goessling et al, 2009). Compared to the vehicle alone group, we observed the *cTnT* (Hsieh et al, 2007) expression and immature sarcomeric structure in the Sca-1^+ cells treated with PGE_2 (Supporting Information Fig 12B and C). Surprisingly, mature sarcomeric organization was seen in the cardiomyocyte-depleted small cells after PGE_2 treatment (Supporting Information Fig 12A and B). The small cell fraction contains several types of differentiated cells and stem cells, including immune cells, fibroblasts and endothelial cells as well as Sca-1^+ cells and mesenchymal stem cells (Beigi et al, 2013; Degousee et al, 2008; Oh et al, 2003; Wong et al, 1998). Therefore, we suspect that PGE_2 not only directly acts on the Sca-1^+ cells but also other cardiac small cells, thereby exerting an indirect effect on cardiomyocyte differentiation modulation. Nevertheless, further investigation is necessary to determine the underlying mechanism of PGE_2-promoted cardiomyocyte differentiation in the small cells.

References:

Degousee N, Fazel S, Angoulvant D, Stefanski E, Pawelzik S-C, Korotkova M, Arab S, Liu P, Lindsay TF, Zhuo S et al (2008) Microsomal Prostaglandin E2 Synthase-1 Deletion Leads to Adverse Left Ventricular Remodeling After Myocardial Infarction. Circulation 117: 1701-1710

Goessling W, North TE, Loewer S, Lord AM, Lee S, Stoick-Cooper CL, Weidinger G, Puder M, Daley GQ, Moon RT et al (2009) Genetic Interaction of PGE2 and Wnt Signaling Regulates Developmental Specification of Stem Cells and Regeneration. Cell 136: 1136-1147

Hsieh PCH, Segers VFM, Davis ME, MacGillivray C, Gannon J, Molkentin JD, Robbins J, Lee RT (2007) Evidence from a genetic fate-mapping study that stem cells refresh adult mammalian cardiomyocytes after injury. Nat Med 13: 970-974

Oh H, Bradfute SB, Gallardo TD, Nakamura T, Gaussin V, Mishina Y, Pocius J, Michael LH, Behringer RR, Garry DJ et al (2003) Cardiac progenitor cells from adult myocardium: Homing, differentiation, and fusion after infarction. Proc Natl Acad Sci USA 100: 12313-12318

Sturzu AC, Wu SM (2011) Developmental and Regenerative Biology of Multipotent Cardiovascular Progenitor Cells. Circ Res 108: 353-364
Wong SCY, Fukuchi M, Melnyk P, Rodger I, Giaid A (1998) Induction of Cyclooxygenase-2 and Activation of Nuclear Factor-{kappa}B in Myocardium of Patients With Congestive Heart Failure. Circulation 98: 100-103

Main text:
Page 5
Among the identified markers, Sca-1 is commonly expressed in various cardiac stem/progenitor cell populations (Oh et al, 2003; Sturzu & Wu, 2011). We therefore sought to investigate the effect of PGE_2 *on stem cell-mediated cardiomyocyte replenishment by examining* $Sca\text{-}1^+$ *cell activities. Quantitative RT-PCR revealed that only Sca-1 expression peaked on day 3 post-MI and this level was further increased at the same time point upon* PGE_2 *treatment but was repressed by indomethacin (Supporting Information Fig 4).*

Page 7
Results from in vitro culture also provided evidence that the expression of Nkx2.5 and cTnT was evidently improved in isolated cardiac small cells and $Sca\text{-}1^+$ *cells by* PGE_2 *(Supporting Information Fig 12B and C). Surprisingly, mature sarcomeric structure, as determined by the expression of cTnT, were seen in the cardiomyocyte-depleted small cells after* PGE_2 *treatment (Supporting Information Fig 12A and B), suggesting the ability of* PGE_2 *to augment cardiomyocyte differentiation.*

Supporting Information Figures:

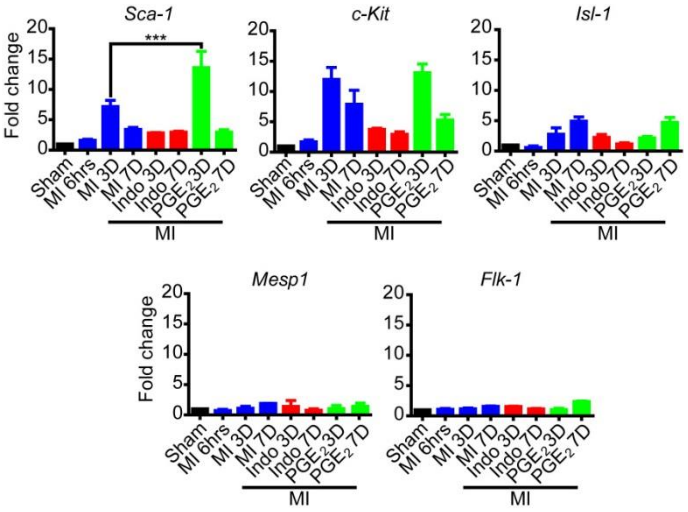

Supporting Information Fig 4. Gene expression of Sca-1 is the most responsive to PGE_2 treatment.
*Expression of the cardiac stem/progenitor marker genes in the infarcted region of injured hearts was analyzed by quantitative RT-PCR. The fold change is a relative quantification normalized to the sham control. ***$p < 0.001$. $n \geq 3$. Data are presented as the mean ± s.e.m. Indo, Indomethacin; MI, myocardial infarction.*

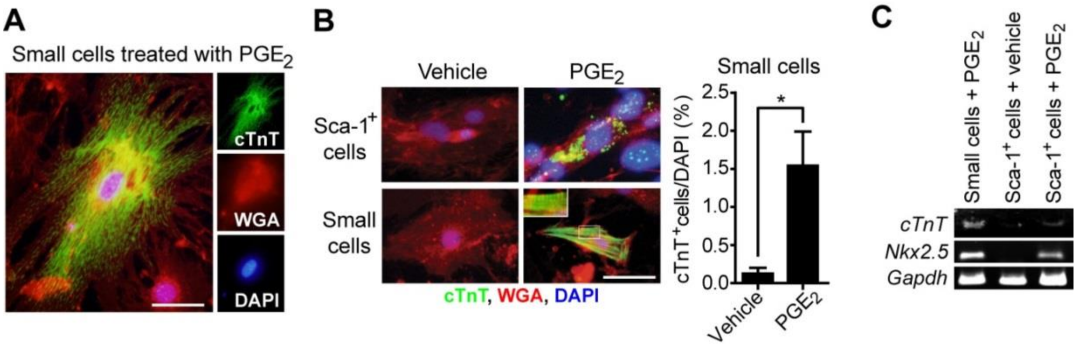

Supporting Information Fig 12. In vitro differentiation ability of isolated cardiac cells into cardiomyocytes.

A. *The cardiomyocyte-depleted small cells were allowed to attach for 3 days followed by PGE_2 treatment (10 μM) for another 3 days. Immunocytochemistry was performed to examine cardiomyocyte differentiation, as determined by the expression of the cardiomyocyte marker cardiac troponin T (cTnT) at day 10. The membrane and nucleus were stained with the membrane dye, WGA, and DAPI, respectively. Scale bar, 50 μm.*

B. *Under the same culture condition, the cardiac Sca-1$^+$ cells and small cells subjected to vehicle or PGE_2 treatment were stained with cTnT for sarcomeric structure analysis. The percentage of small cells with mature sarcomeric structures following vehicle or PGE_2 treatment was quantified. Scale bar, 50 μm. *$p < 0.05$. Data are presented as mean ± s.e.m.*

C. *Following the same culture procedure, the effect of PGE_2 on the expression of cardiac marker genes, Nkx2.5 and cTnT, in Sca-1$^+$ cells was analyzed by semi-quantitative PCR. Small cells treated with PGE_2 serve as positive control.*

c. (Pg 5 para 3) Despite the authors' suggestions, the underlying mechanism of PGE2's salutary effect is not determined. Does it operate on macrophages, progenitors, HSCs, or other cells? The authors cite work from North et al (2007) and Hoggatt et al (2009) indicating that PGE2 effects HSC mobilisation. HSC mobilisation has been previously indicated to improve cardiac function after injury. The authors do not pursue the mechanistic basis of their observations.

We thank the Reviewer for raising this important issue. Result from quantitative RT-PCR has revealed that the stem cell marker gene that was most responsive to PGE_2 treatment was *Sca-1* (Supporting Information Fig 4). To further dissect the role of PGE_2 in regulating cardiomyocyte differentiation, we first analyzed the expression of the four PGE_2 receptors, *EP1-EP4* (Hoggatt et al, 2009), in hearts subjected to MI surgery with or without Indomethacin or PGE_2 treatment. We observed that the expression of *EP*2 was significantly higher than that of *EP1*, *EP3* and *EP4* after MI, which implies that EP2 plays a major role in cardiomyocyte regeneration (Supporting Information Fig 9). Furthermore, administration of PGE_2 increased expression of *EP2* in the Sca-1$^+$ cells isolated from the heart at day 3 post-MI (Supporting Information Fig 10). To further confirm its role in cardiomyocyte regeneration, an independent experiment using EP2 knockout (EP2$^{-/-}$) mice (Kennedy et al, 1999) was performed. We isolated Sca-1$^+$ cells from EP2$^{-/-}$ mice and injected them directly into the infarcted M/Z hearts (Supporting Information Fig 11A). In contrast to the hearts receiving wild-type cardiac Sca-1$^+$ cells (EP2$^{+/+}$), injection of EP2$^{-/-}$ Sca-1$^+$ cells did not change the proportion of GFP$^+$ or β-Gal$^+$ cardiomyocytes (Figure 2F, Supporting Information Fig 11B). These findings further support the notion that cardiac Sca-1$^+$ stem/progenitor cell may be a source for cardiomyocyte replenishment and that the COX-2/PGE_2/EP2 pathway plays a key role in modulating this process.

In addition to the Sca-1$^+$ cells, we also observed that PGE_2 treatment alters the proportion of M2 macrophages. We took the Reviewer's suggestion in **Question d** and re-examined the effect of PGE_2 on M1 (F4/80$^+$/Gr-1$^+$) and M2 (F4/80$^+$/CD206$^+$) macrophages using flow cytometry and appropriate antibodies. At day 3 post-MI, PGE_2 treatment increased the number of M2 macrophages by approximately 2-fold (Supporting Information Fig 13). Therefore, PGE_2 may also act on the inflammatory cells and modulate the inflammatory micro-environment after heart injury.

In addition to the *in vivo* study, we also conducted an *in vitro* culture experiment to examine the effect of PGE_2 on cardiac differentiation of Sca-1$^+$ cells and the cardiomyocyte-depleted small cells, a population that may contain endothelial cells, fibroblasts, immune cells and Sca-1$^+$ cells (Beigi et al, 2013; Degousee et al, 2008; Oh et al, 2003; Wong et al, 1998). Following PGE_2 treatment, Sca-1$^+$ cells had higher expression level of *cTnT* and immature sarcomeric organization (Supporting Information Fig 12B and C). Mature sarcomere was seen in the cardiomyocyte-depleted small cells after PGE_2 treatment (Supporting Information Fig 12A and B). Collectively, these findings demonstrate that PGE_2 not only directly acts on the Sca-1$^+$ stem/progenitor cells, other cardiac small cells, likely the macrophages, may also be responsive to PGE_2 and thereby exerting an indirect effect on modulating cardiomyocyte differentiation. All the data have been incorporated in the revised manuscript.

References:

Degousee N, Fazel S, Angoulvant D, Stefanski E, Pawelzik S-C, Korotkova M, Arab S, Liu P, Lindsay TF, Zhuo S et al (2008) Microsomal Prostaglandin E2 Synthase-1 Deletion Leads to Adverse Left Ventricular Remodeling After Myocardial Infarction. Circulation 117: 1701-1710

Hoggatt J, Singh P, Sampath J, Pelus LM (2009) Prostaglandin E2 enhances hematopoietic stem cell homing, survival, and proliferation. Blood 113: 5444-5455

Kennedy CRJ, Zhang Y, Brandon S, Guan Y, Coffee K, Funk CD, Magnuson MA, Oates JA, Breyer MD, Breyer RM (1999) Salt-sensitive hypertension and reduced fertility in mice lacking the prostaglandin EP2 receptor. Nat Med 5: 217-220

Oh H, Bradfute SB, Gallardo TD, Nakamura T, Gaussin V, Mishina Y, Pocius J, Michael LH, Behringer RR, Garry DJ et al (2003) Cardiac progenitor cells from adult myocardium: Homing, differentiation, and fusion after infarction. Proc Natl Acad Sci USA 100: 12313-12318

Wong SCY, Fukuchi M, Melnyk P, Rodger I, Giaid A (1998) Induction of Cyclooxygenase-2 and Activation of Nuclear Factor-{kappa}B in Myocardium of Patients With Congestive Heart Failure. Circulation 98: 100-103

Main text:
Page 6-7

Because tamoxifen injection in M/Z mice leads to conversion of β-Gal to GFP in cardiomyocytes, we thought to take this advantage to examine cardiomyogenic differentiation ability of the cardiac Sca-1$^+$ cells. The tamoxifen injection was given to the M/Z mice after MI surgery, and therefore, only α-MHC$^+$ cells would express GFP (Supplementary Information Fig 7A). This experiment allowed us to determine whether Sca-1$^+$ cells possess the ability to differentiate into α-MHC$^+$ cells. Following MI surgery and tamoxifen injection for 3 days, Sca-1$^+$/GFP$^+$ cells could be detected. The percentage of double positive cells was further increased upon PGE_2 treatment (Supplementary Information Fig 7B and C). In addition, Sca-1$^+$/α-MHC$^+$ cells were not observed before tamoxifen labeling and they do not arise from cardiomyocyte de-differentiation (Hsieh et al, 2007; Senyo et al., 2013) (Supplementary Information Fig 8). These results reveal the potential contribution of cardiac Sca-1+ stem/progenitor cells to cardiomyocyte replenishment after MI.

Following MI, M/Z system serves as a platform to assess the cardiomyocytes differentiated from endogenous stem/progenitor cells. To evaluate the cardiomyocyte differentiation ability of cardiac Sca-1^{+} cells and the importance of PGE_2 pathway during this process, the cells were isolated from wild-type and EP2$^{-/-}$ mice (Kennedy et al, 1999) for intramyocardial injection after MI surgery (Loffredo et al, 2011; Supporting Information Fig 11A). The EP2$^{-/-}$ transgenic mouse was chosen due to expression of this PGE_2 receptor was significantly induced in hearts after MI and in cardiac Sca-1^{+} cells after PGE_2 treatment (Supporting Information Fig 9 and 10). Quantification of the GFP^{+} and β-Gal^{+} cardiomyocyte numbers revealed that injection of wild-type Sca-1^{+} cells reduced both GFP^{+} and β-Gal^{+} cardiomyocyte numbers and that approximately 10% of the peri-infarct cardiomyocytes were GFP^{-} and β-Gal^{-}, suggesting cardiomyocyte differentiation of the injected cardiac Sca-1^{+} cells. In contrast, we did not observe such change in the M/Z mice receiving injection of EP2$^{-/-}$ Sca-1^{+} cells (Fig. 2F, Supporting Information Fig 11). Together these results indicate that the PGE_2/EP2 signaling may regulate the ability of cardiac Sca-1^{+} cells to differentiate into cardiomyocytes.

Page 7

Macrophages can be classified into M1 (CDF4/80^{+}/Gr-1^{+}) and M2 (F4/80^{+}/CD206^{+}) subtypes (Nishimura et al, 2009; Vandanmagsar et al, 2011). Interestingly, flow cytometry analysis revealed that PGE_2 treatment elevated the number of M2 macrophages after MI (Supporting Information Fig 13).

Figures and Supporting Information Figures:

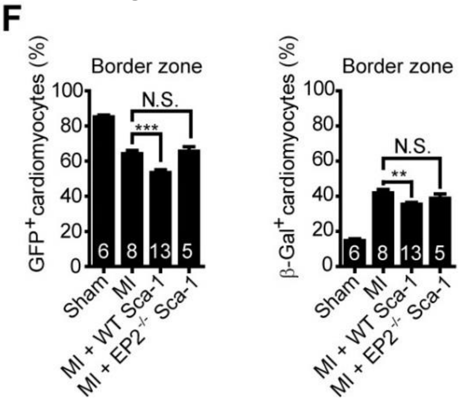

Figure 2. COX-2-dependent signaling pathway stimulates cardiomyocyte replenishment with endogenous stem/progenitor cells shortly after infarction.

F. Following DAB staining, the percentages of GFP^{+} and β-Gal^{+} cardiomyocytes at the border zone of the young heart with or without cell injection after MI were quantified and statistically analyzed. Sample size is indicated in the bar chart. $**p< 0.01$, $***p< 0.001$; N.S., not significant. Data are presented as the mean ± s.e.m.

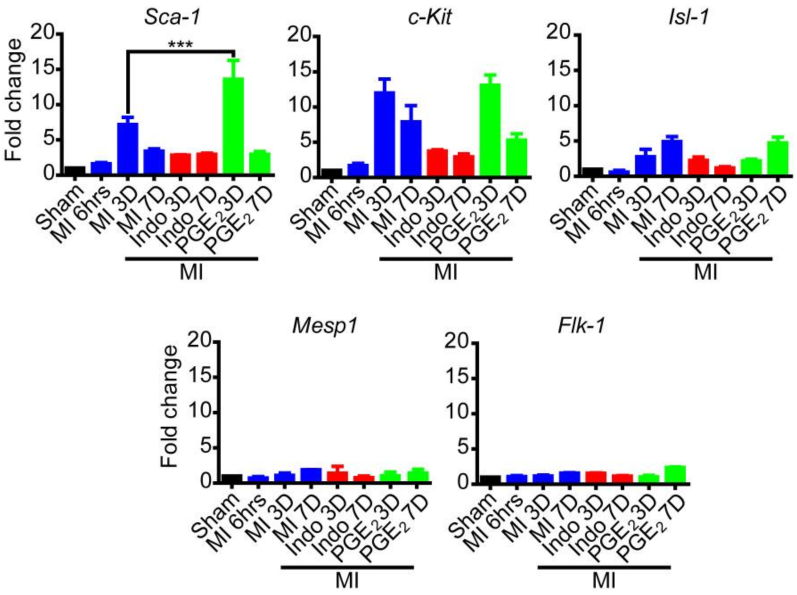

Supporting Information Fig 4. Gene expression of Sca-1 is the most responsive to PGE$_2$ treatment.

*Expression of the cardiac stem/progenitor marker genes in the infarcted region of injured hearts was analyzed by quantitative RT-PCR. The fold change is a relative quantification normalized to the sham control. ***p< 0.001. n ≥ 3. Data are presented as the mean ± s.e.m. Indo, Indomethacin; MI, myocardial infarction.*

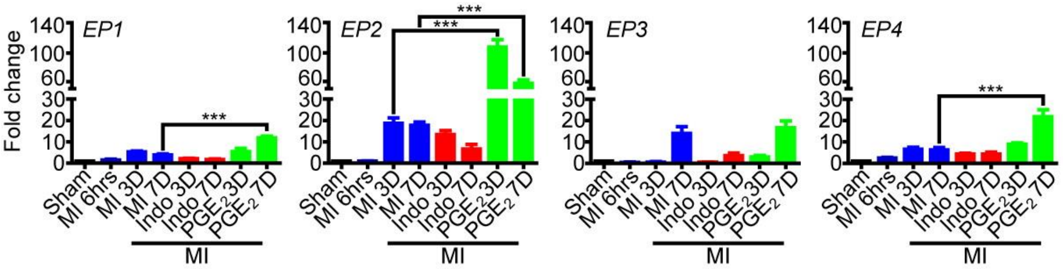

Supporting Information Fig 9. PGE$_2$ increases the expression of EP2 receptor after myocardial infarction.

*The expression of PGE$_2$ receptors, EP1, 2, 3 and 4, in response to different drug treatments at the infarct region of the injured heart was examined by quantitative RT-PCR. The fold change is a relative quantification normalized to the sham control. ***p<0.001. Data are presented as mean ± s.e.m. Indo, Indomethacin; MI, myocardial infarction.*

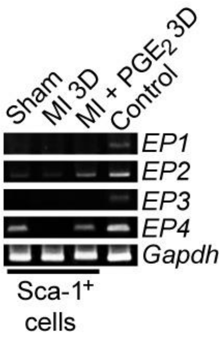

Supporting Information Fig 10. The expression of EP2 receptor increases in Sca-1$^+$ cells isolated from the infarcted heart after PGE$_2$ treatment.

Semi-quantitative PCR was performed to examine the expression of PGE_2 receptors, EP1, 2, 3 and 4, in cardiac Sca-1^+ cells isolated after myocardial infarction (MI). Un-sorted cardiomyocyte-depleted small cells from the sham group served as positive control.

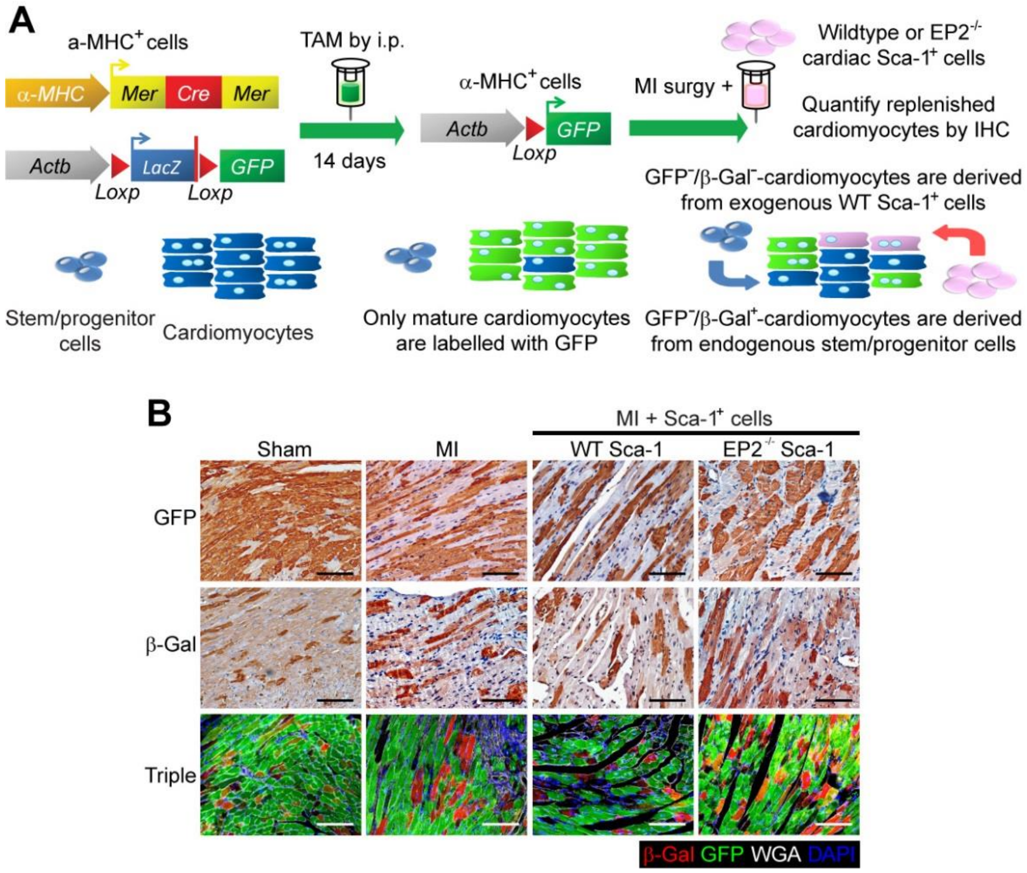

Supporting Information Fig 11. Quantification of the degree of cardiomyocyte replenishment after Sca-1^+ cell injection in injured hearts.

A. *Schematic diagram depicting the experimental procedure. Following 14 days of tamoxifen injection, the M/Z mice were injected intramyocardially with wild-type or EP2 knockout ($EP2^{-/-}$) cardiac Sca-1^+ cells after myocardial infarction (MI). The hearts were harvested at day 14 post-MI for examination.*

B. *At day 14 post-infarction, the hearts were harvested for DAB and immunofluorescence triple staining to examine the GFP^+ or β-Gal^+ cardiomyocytes. Shown are representative images from each group. Scale bars, 100 μm.*

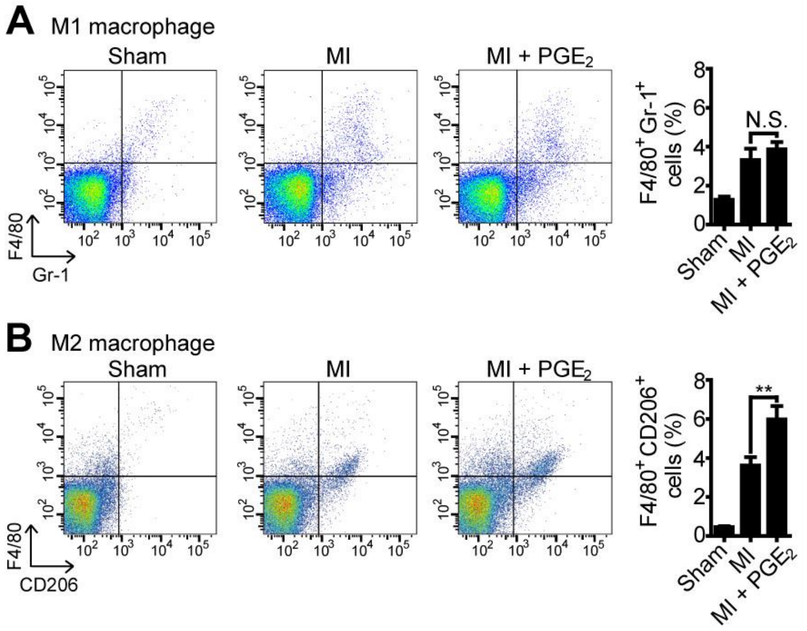

Supporting Information Fig 13. PGE$_2$ increases the number of M2 type macrophages in the myocardium after injury.

*A-B. At day 3 post-surgery, the infarcted heart was enzymatically digested for flow cytometric and statistical analyses of M1 macrophages (F4/80$^+$/Gr-1$^+$) and M2 macrophages (F4/80$^+$/CD206$^+$). Data are presented as mean ± s.e.m. **$p<0.005$. MI, myocardial infarction.*

d. The quantification of M1 and M2 macrophages conducted does not support any of the authors' conclusions.
i. CD11c is not an M1 macrophage marker.
ii. CD11b is a pan myeloid marker within leukocytes and not exclusive to macrophages.
iii. The approach used is not appropriate. To adequately address this question, a flow cytometry experiment is necessary. This can be easily achieved using CD45, CD11b, F4/80, Ly6c (Gr-1) and CD206 antibodies. This is a very simple experiment and is essential to conclusively determine if macrophage polarization is affected by PGE2.

We thank the Reviewer for raising this concern and providing helpful suggestions. Quantification of M1 ($F4/80^+/Gr\text{-}1^+$) and M2 ($F4/80^+/CD206^+$) macrophages has been performed using flow cytometry and appropriate markers as suggested. We observed that PGE_2 treatment greatly increased the proportion of M2 macrophages by 2-fold after MI. This finding suggests that PGE_2 has a role in modulating the inflammatory micro-environment after MI by acting on macrophages. The data have been incorporated into the revised manuscript as Supporting Information Fig 13.

Supporting Information Figure:

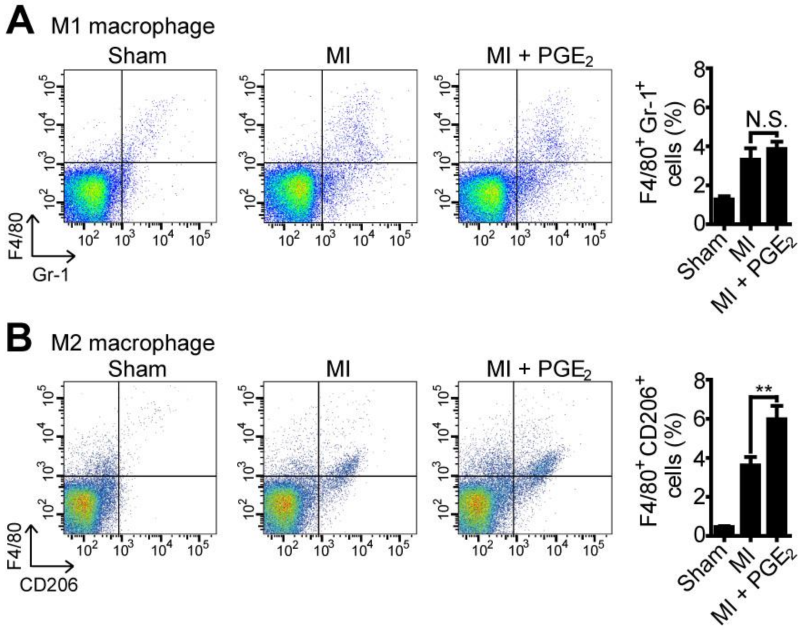

Supporting Information Fig 13. PGE_2 increases the number of M2 type macrophages in the myocardium after injury.

A-B. At day 3 post-surgery, the infarcted heart was enzymatically digested for flow cytometric and statistical analyses of M1 macrophages ($F4/80^+/Gr\text{-}1^+$) and M2 macrophages ($F4/80^+/CD206^+$). Data are presented as mean ± s.e.m. $^{**}p<0.005$. MI, myocardial infarction.

e. (Pg 8 para 2) It cannot be concluded that PGE2 is enhancing cardiopoiesis of injured aged hearts by modulating TGFβ1 activity.

We thank the Reviewer for this comment. We have removed the paragraph from the manuscript.

f. All inhibitor studies are non-tissue specific. The effect of these reagents on systemic elements, such as the bone marrow/HSC niches, spleen etc cannot be delineated. This must be discussed in the discussion along with the important effect of PGE2 in HSC mobilisation, which is salutary for the injured heart.

We thank the Reviewer for raising this issue and a new paragraph has been incorporated into the revised manuscript for discussion.

Discussion:
Page 10
The role of PGE_2 in modulating stem cell function has been reported in the bone marrow, where it regulates hematopoietic stem cell (HSC) homeostasis (North et al, 2007) and improves their functions, including survival and proliferation (Hoggatt et al, 2009). A recent study reported by Hoggatt et al. demonstrates that PGE_2 facilitates the retention of the HSCs in the bone marrow and non-steroidal anti-inflammatory drug (NSAID) induces HSC egress (Hoggatt et al, 2013). PGE_2 has also been reported to modulate HSC activities, including cell differentiation and survival (Hoggatt et al, 2009; Goessling et al, 2009). Because Indomethacin and Celecoxib are NSAIDs, they may exert the same effect on the HSCs. On the basis of our results, we suspect that the microenvironment in the infarct heart could be disturbed following mobilization of un-differentiated HSCs, and consequently attenuates cardiomyocyte regeneration efficiency. However, administration of PGE_2 restores this regenerative machinery by acting on the cardiac stem/progenitor cells and inflammatory cells. We provide evidence to demonstrate that PGE_2 directly regulates cardiac Sca-1$^+$ cells, implying a possible role of NSAID in mediating cardiac stem/progenitor cell mobilization. In addition, PGE_2 also increases the number of M2 macrophages. Based on these findings and previous studies showing PGE_2-dependent modulation of HSC activities, we speculate that how PGE_2 regulates HSCs after MI is also an important factor for cardiomyocyte regeneration.

2. General queries/comments:
a. Fig 1b: How many days after injury were the images taken?

The photos were taken at day 14 post-MI.

b. Fig 2a. Does Celecoxib have different outcomes when administered early vs late (similar to the indomethacin experiment)?
c. Does PGE2 administration rescue Celecoxib treated mice, similar to Indomethacin treated animals? No data of PGE2 Celecoxib treatment.

We thank the Reviewer for these important questions regarding the effects of Celecoxib treatment on the status of cardiomyocyte replenishment. Detailed explanation for the outcome of Celecoxib is given above in **Question a (Major concerns)**. To address these questions, the mice were administrated with Celecoxib at early and late time points after heart injury or simultaneously with PGE_2 for 14 days post-MI. Our results reveal that early administration of Celecoxib effectively abolishes cardiomyocyte replenishment by stem/progenitor cells (Supporting Information Fig 3). We did not observe such inhibitory

effect when the drug was given at a later time point post-injury. In addition, simultaneous administration of PGE_2 could restore cardiomyocyte repopulating efficiency (Supporting Information Fig 3). These data further support the role of COX-2/PGE_2 signaling pathway during cardiomyocyte replenishment.

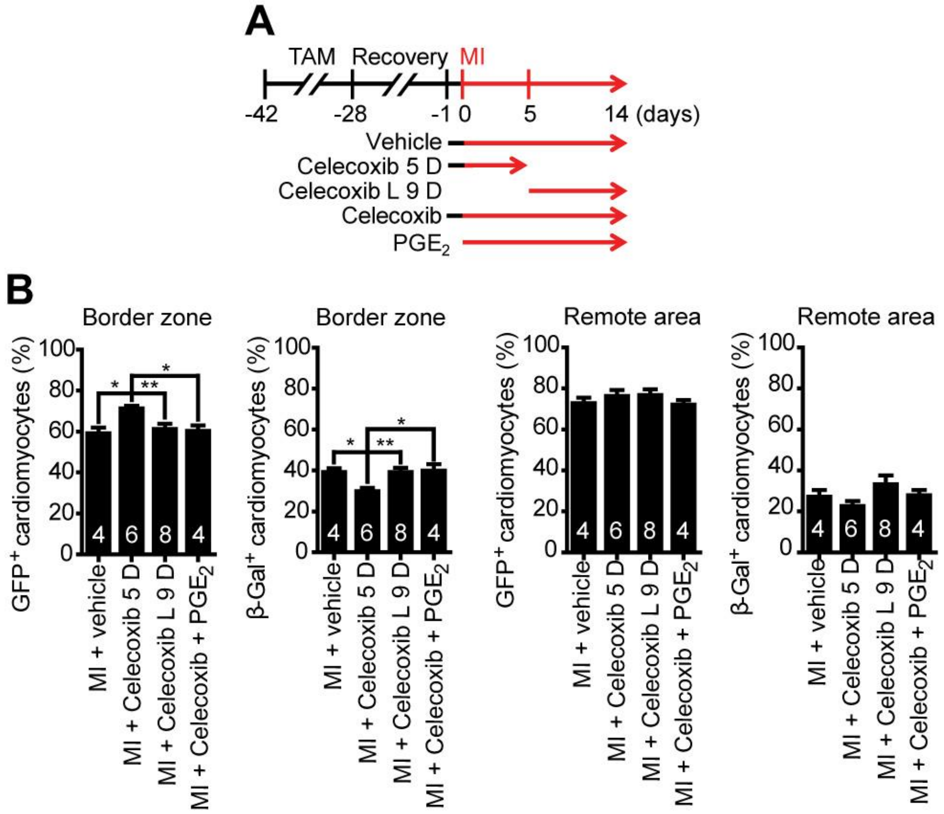

Supporting Information Fig 3. Early COX-2 signalling pathway is necessary for cardiomyocyte regeneration.

A. *Experimental paradigm of drug treatment on the MerCreMer/ZEG mice. Celecoxib was continuously administered 1 day before surgery until day 5 (Celecoxib 5 D) after MI or from day 5 to day 14 post-MI (Celecoxib L 9 D). Another group of animals was administrated with Celecoxib and PGE_2 simultaneously for 14 days. The hearts from drug-treated mice isolated at day 14 post-infarction were stained for GFP or β-Gal.*

B. *GFP^+ and β-Gal^+ cells at the border zone or remote area were quantified and statistically analyzed. *$p<0.05$, **$p<0.01$. Data are presented as the mean ± s.e.m. Sample size is indicated in the bar chart. MI, myocardial infarction.*

d. To confirm PGE2 is having a effect by directly acting upon progenitors or macrophages, a conditional EP2 and/or EP4 receptor ablation (for example in macrophages) experiment is necessary. Without this experiment, it is not possible to conclude how PGE2 is having a salutary effect after injury.

We are grateful to the Reviewer for raising this important question. The detailed explanation regarding mechanistic examination of PGE_2 is given above in **Question c (Major concerns)**. We discovered that the expression of stem cell marker gene *Sca-1* was significantly elevated by PGE_2 (Supporting Information Fig 4). In addition, PGE_2-promoted *EP2* expression was observed not only in the MI heart but also in the cardiac Sca-1^+ cells isolated after heart injury (Supporting Information Fig 10). Furthermore, injection of wild-type cardiac Sca-1^+ cells that did not carry any reporter resulted in reduction in percentage of GFP^+ and β-Gal^+ cardiomyocytes to non-labeled

cardiomyocytes, which may have been derived from the injected Sca-1$^+$ cells. However, this phenomenon was not observed in M/Z hearts receiving the EP2 knockout cardiac Sca-1$^+$ cells (Fig 2F, Supporting Information Fig 11). These findings further support the notion that cardiac Sca-1$^+$ cells may be a source for cardiomyocyte replenishment and that the COX-2/PGE_2/EP2 pathway plays a key role in modulating this process. We have incorporated these data in the revised manuscript.

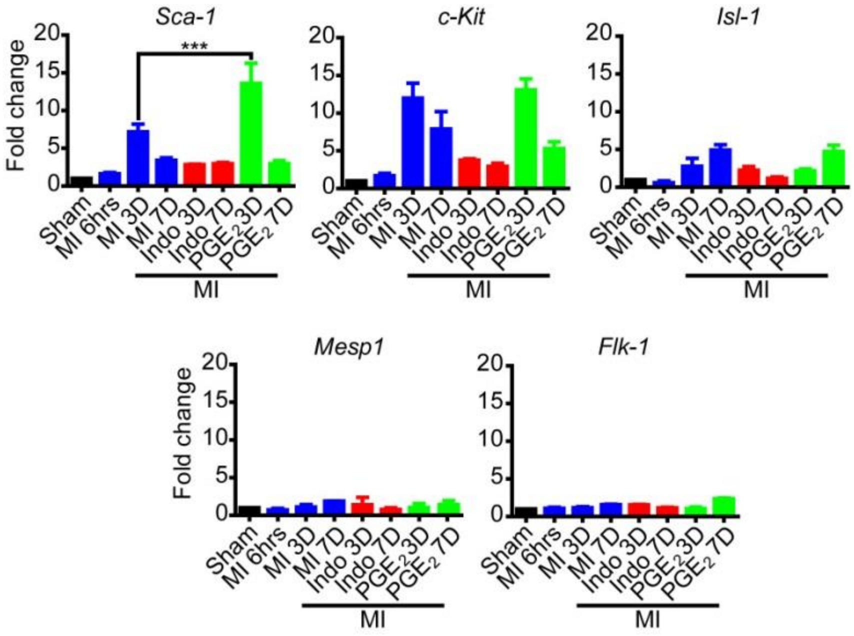

Supporting Information Fig 4. Gene expression of Sca-1 is the most responsive to PGE_2 treatment.
*Expression of the cardiac stem/progenitor marker genes in the infarcted region of injured hearts was analyzed by quantitative RT-PCR. The fold change is a relative quantification normalized to the sham control. ***p< 0.001. n ≥ 3. Data are presented as the mean ± s.e.m. Indo, Indomethacin; MI, myocardial infarction.*

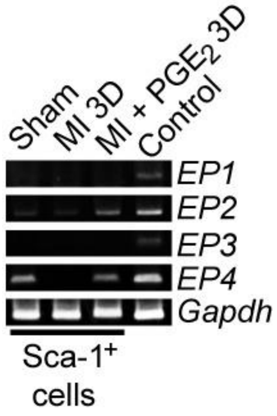

Supporting Information Fig 10. The expression of EP2 receptor increases in Sca-1$^+$ cells isolated from the infarcted heart after PGE_2 treatment.
Semi-quantitative PCR was performed to examine the expression of PGE_2 receptors, EP1, 2, 3 and 4, in cardiac Sca-1$^+$ cells isolated after myocardial infarction (MI). Un-sorted cardiomyocyte-depleted small cells from the sham group served as positive control.

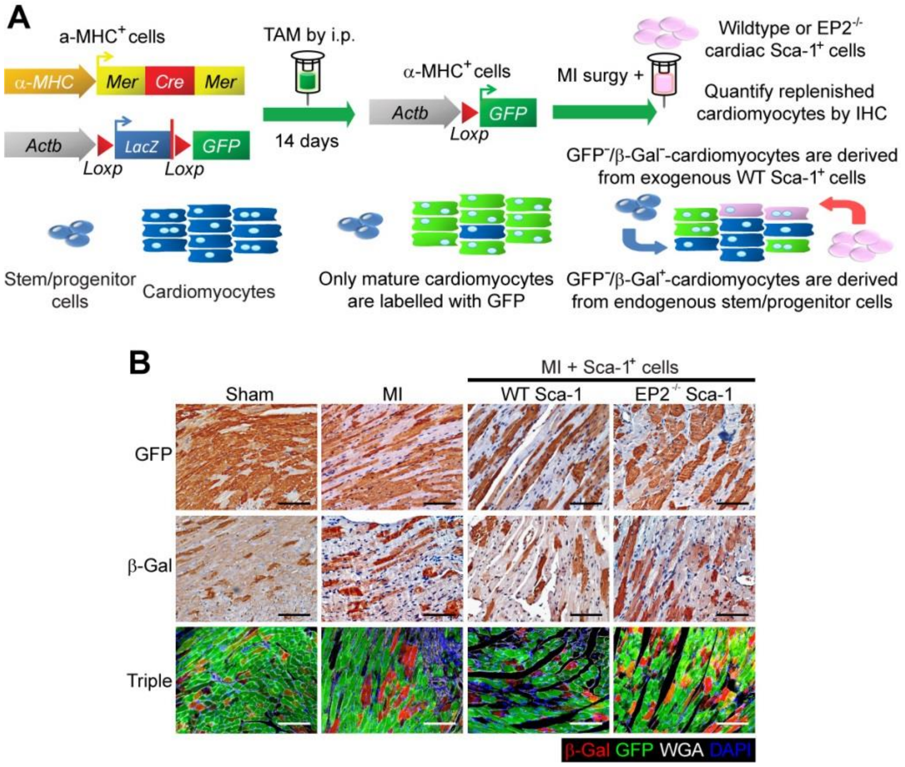

Supporting Information Fig 11. Quantification of the degree of cardiomyocyte replenishment after Sca-1$^+$ cell injection in injured hearts.

A. *Schematic diagram depicting the experimental procedure. Following 14 days of tamoxifen injection, the M/Z mice were injected intramyocardially with wild-type or EP2 knockout (EP2$^{-/-}$) cardiac Sca-1$^+$ cells after myocardial infarction (MI). The hearts were harvested at day 14 post-MI for examination.*
B. *At day 14 post-infarction, the hearts were harvested for DAB and immunofluorescence triple staining to examine the GFP$^+$ or β-Gal$^+$ cardiomyocytes. Shown are representative images from each group. Scale bars, 100 μm.*

e. Fig S2: Where in the infarcted heart has the image been taken? The image of the CD11b staining could not be of the cardiac lesion after MI 3 days after injury. At this location, at this time point, it is extremely difficult to even see individual CD11b cells due to the massive influx of myeloid cells.

We thank the Reviewer for noting this concern. The images were taken at the border zone, a region adjacent to the infarct tissue but contains surviving cardiomyocytes, rather than at the site of lesion. For more accurate quantification of macrophages, we have performed flow cytometry analysis using appropriate antibodies as suggested by the Reviewer in **Question d (major concerns**). The IHC staining results of macrophages are therefore removed in the revised manuscript.

f. Why there is an improvement of heart function 2 months after injury, but not 1 month, should be addressed.

We thank the Reviewer for pointing out this question. Although in the absence of PGE_2 treatment, we still observed worse cardiac function of MI heart at 2 months than at 1 month, likely due to an increase in the infarct lesion and elevated number of dead cardiomyocytes over time. Following PGE_2 treatment, repopulation of cardiomyocytes by

stem/progenitor cells is increased by ~9%. We therefore speculate these stem/progenitor cell-derived cardiomyocytes not only contribute to repopulation of lost cardiomyocytes but also ameliorate the degree of infarct lesion over time. The exact time when an improvement in cardiac function is expected following stem/progenitor cell-modulated cardiomyocyte repopulation remains unclear. Loffredo *et al*. demonstrated that cardiac function was improved by recovered ability for endogenous stem/progenitor cells to repopulate cardiomyocytes at 2 months post-MI following cell injection-based therapy, which is consistent with our findings. Furthermore, our results suggest that the stem/progenitor cells, but not the pre-existing cardiomyocytes, are the dominant population targeted by PGE_2. Therefore, we suspect that improvement in cardiac function would not be as profound as drug or cell therapies aimed at protecting cardiomyocytes from apoptosis.

Reference:

Loffredo Francesco S, Steinhauser Matthew L, Gannon J, Lee Richard T (2011) Bone Marrow-Derived Cell Therapy Stimulates Endogenous Cardiomyocyte Progenitors and Promotes Cardiac Repair. Cell Stem Cell 8: 389-398

g. What is the age of the 'young' mice?

The young mice are 3 to 5 months of age.

h. Acknowledgments: The supporting institutes are from which countries?

From Taiwan. We have clarified this in the Acknowledgment.

Referee #3:

1) First of all this manuscript does not reflect/discuss the controversial character of this study and the conflicting results to other studies. For example, there is to this reviewers knowledge no study available that unambiguously demonstrates that stem cells differentiate into cardiomyocytes in vivo. In addition, the recent data by Senyo suggest that the replenishment of cardiomyocytes is difficult to explain by stem cell differentiation. At least, the data suggest that existing stem/progenitor cells do not proliferate. As these cells are difficult to find it is hard to explain how they generate 10% new cardiomyocytes. This should at least be discussed as well as possible problems of the assay.

We thank the Reviewer for raising the concern. The study reported by Senyo et al suggests that the pre-existing cardiomyocytes are the major population to replenish lost cells (Senyo et al, 2013). Despite the ^{15}N labeling system was used to evaluate the contribution of pre-existing cardiomyocytes in the injured heart, a dilution in GFP$^+$ cell pool after myocardial infarction (MI) could not be explained. Therefore, the participation of endogenous stem/progenitor cells in cardiomyocyte regeneration cannot be excluded. To provide further evidence that the M/Z system is a suitable approach to examine stem/progenitor cell-dependent cardiac repair, *in vitro* and *in vivo* experiments were performed to investigate the role of PGE_2 in cardiomyocyte regeneration.

To identify the stem cell population responsive to the PGE_2 treatment, we performed quantitative RT-PCR to analyze the expression of several stem/progenitor cell marker genes. Of all the genes analyzed, the expression of *Sca-1* (Oh et al, 2003; Sturzu & Wu, 2011) was most significantly enhanced by PGE_2 (Supporting Information Fig 4). Furthermore, administration of PGE_2 increased expression of *Nkx2.5* (Wu et al, 2006) in the Sca-1$^+$ cells isolated from the heart at day 3 post-MI (Supporting Information Fig 6). Because tamoxifen injection in M/Z mice leads to the conversion of β-Gal to GFP in cardiomyocytes, we thought to take this advantage to examine the cardiomyogenic differentiation ability of cardiac Sca-1$^+$ cells. The tamoxifen injection was given to the M/Z mice after MI surgery, and therefore, only α-MHC$^+$ cells would express GFP. This experiment allowed us to determine whether Sca-1$^+$ cells possess the ability to differentiate into α-MHC$^+$ cells. Following MI surgery and tamoxifen injection for 3 days, Sca-1$^+$/GFP$^+$ cells could be detected (Supplementary Information Fig 7A). The percentage of double positive cells further increased upon PGE_2 treatment (Supplementary Information Fig 7B and C). In addition, Sca-1$^+$/α-MHC$^+$ cells were not observed before tamoxifen labeling and the population did not arise from cardiomyocyte de-differentiation (Hsieh et al, 2007; Senyo et al, 2013; Supplementary Information Fig 8). These results reveal the potential contribution of cardiac Sca-1$^+$ stem/progenitor cells to cardiomyocyte replenishment after MI.

Next, we investigated the effect of PGE_2 on cardiomyogenic differentiation of cardiac Sca-1$^+$ cells. Following isolation of Sca-1$^+$ cells and cardiomyocyte-depleted small cells from young mice, the cells were cultured on fibronectin-coated plates for 3 days, as it took at least 3 days for the cells to attach to the plate (Oh et al, 2003).Then, cells were treated with PGE_2 for another 3 days and their cardiomyocyte differentiation potential was examined at day 10 (Goessling et al, 2009). Compared to the vehicle alone group, we observed *cTnT* (Hsieh et al, 2007) expression and immature sarcomeric structure in the Sca-1$^+$ cells treated with PGE_2 (Supporting Information Fig 12B and C). Surprisingly, mature sarcomeric organization was seen in the cardiomyocyte-depleted small cells after PGE_2 treatment (Supporting Information Fig 12A and B). The small cell fraction

contains several types of differentiated cells and stem cells, including immune cells, fibroblasts, endothelial cells as well as Sca-1$^+$ cells and mesenchymal stem cells (Beigi et al, 2013; Degousee et al, 2008; Oh et al, 2003; Wong et al, 1998). Therefore, we suspect that PGE_2 not only directly acts on the Sca-1$^+$ stem/progenitor cells but also other cardiac small cells thereby exerting an indirect effect on modulating cardiomyocyte differentiation. Further investigation is necessary to determine the underlying mechanism of PGE_2-promoted cardiomyocyte differentiation in the small cells.

To further dissect the role of PGE_2 in regulating cardiomyocyte differentiation, we first analyzed the expression of the PGE_2 receptors *EP1-EP4* (Hoggatt et al, 2009) in hearts subjected to MI surgery with or without Indomethacin or PGE_2 treatment. We observed that *EP*2 expression was significantly higher than *EP1*, *EP3* and *EP4* expression after MI (Supporting Information Fig 9). Furthermore, *EP2* expression was also induced in cardiac Sca-1$^+$ cells after MI (Supporting Information Fig 10). These findings imply that EP2 plays a major role in cardiomyocyte regeneration. To confirm this, an independent experiment using EP2 knockout (EP2$^{-/-}$; Kennedy et al, 1999) mice was performed (Supporting Information Fig 11A). We isolated Sca-1$^+$ cells from the EP2$^{-/-}$ mice and injected them into infarcted M/Z hearts (Loffredo et al, 2011). In contrast to the hearts receiving wild-type cardiac Sca-1$^+$ cells (EP2$^{+/+}$), injection of EP2$^{-/-}$ Sca-1$^+$ cells did not change the proportion of GFP$^+$ or β-Gal$^+$ cardiomyocytes (Figure 2F, Supporting Information Fig 11B). These findings further support the notion that cardiac Sca-1$^+$ stem/progenitor cells may be a source of cardiomyocyte replenishment and that the COX-2/PGE_2/EP2 pathway plays a key role in modulating this process. The new *in vitro* and *in vivo* data have been incorporated in the revised manuscript.

Results in Senyo's study (Senyo et al, 2013) suggest that the main cell source to replenish cardiomyocytes after heart injury is the pre-existing cardiomyocytes. However, the contribution of stem/progenitor cells cannot be excluded in their study. It is also possible that the stem/progenitor cell-derived cardiomyocytes generated at an early time point may be labeled following long-term isotope tracing. A commentary article reported by Palacios J.A. & Schneider M.D. in EMBO Molecular Medicine has pointed out that the number of cells, 35 $^{15}N^+$ cardiomyocytes out of 4,000 cells examined, reported by Senyo et al. is too small, suggesting potential controversy of their finding. It may not be objective to evaluate the proliferation ability of different cell populations based solely on small sample size (Palacios & Schneider, 2013). Malliaras *et al*. used flow cytometry for global quantification of cardiomyocyte proliferation rate, as determined by BrdU incorporation, in MI heart with the M/Z system (Malliaras et al, 2013). They provided evidence that stem/progenitor cell-derived GFP$^-$ cardiomyocytes have better proliferation ability than the residing GFP$^+$ cardiomyocytes after MI, which is in the contrary to the results reported by Senyo et al. Furthermore, the number of GFP$^-$ cardiomyocytes entering cell cycle could be greatly amplified by cell transplantation therapy. Therefore, it remains inconclusive whether the pre-existing cardiomyocytes are the major contributor for cardiomyocyte replenishment and further examinations are necessary. On the other hand, both our and Malliaras *et al*.'s results support the notion that targeting the stem/progenitor cells for cardiac repair remains an important therapeutic approach (Malliaras et al, 2013).

Collectively, our results are not in conflict with Senyo's. Despite that the ^{15}N was used to label the pre-existing cardiomyocytes entering the cell cycle after MI, a 15% dilution in the GFP$^+$ cardiomyocyte pool could still be observed (Senyo et al, 2013). Furthermore, contribution of stem/progenitor cell-derived cardiomyocytes to the GFP$^+$ cell pool dilution was not excluded based on their experimental design. Senyo's study strengthens the necessity of stem/progenitor cell research for cardiac repair because only 0.002%

(16/7063) of pre-existing cardiomyocytes complete cell division while the GFP$^+$ cells could be diluted by 15%. We have incorporated these new results and discussion into the revised manuscript as the following.

References:

Degousee N, Fazel S, Angoulvant D, Stefanski E, Pawelzik S-C, Korotkova M, Arab S, Liu P, Lindsay TF, Zhuo S et al (2008) Microsomal Prostaglandin E2 Synthase-1 Deletion Leads to Adverse Left Ventricular Remodeling After Myocardial Infarction. Circulation 117: 1701-1710

Goessling W, North TE, Loewer S, Lord AM, Lee S, Stoick-Cooper CL, Weidinger G, Puder M, Daley GQ, Moon RT et al (2009) Genetic Interaction of PGE2 and Wnt Signaling Regulates Developmental Specification of Stem Cells and Regeneration. Cell 136: 1136-1147

Hoggatt J, Singh P, Sampath J, Pelus LM (2009) Prostaglandin E2 enhances hematopoietic stem cell homing, survival, and proliferation. Blood 113: 5444-5455

Hsieh PCH, Segers VFM, Davis ME, MacGillivray C, Gannon J, Molkentin JD, Robbins J, Lee RT (2007) Evidence from a genetic fate-mapping study that stem cells refresh adult mammalian cardiomyocytes after injury. Nat Med 13: 970-974

Kennedy CRJ, Zhang Y, Brandon S, Guan Y, Coffee K, Funk CD, Magnuson MA, Oates JA, Breyer MD, Breyer RM (1999) Salt-sensitive hypertension and reduced fertility in mice lacking the prostaglandin EP2 receptor. Nat Med 5: 217-220

Loffredo Francesco S, Steinhauser Matthew L, Gannon J, Lee Richard T (2011) Bone Marrow-Derived Cell Therapy Stimulates Endogenous Cardiomyocyte Progenitors and Promotes Cardiac Repair. Cell Stem Cell 8: 389-398

Malliaras K, Zhang Y, Seinfeld J, Galang G, Tseliou E, Cheng K, Sun B, Aminzadeh M, Marbán E (2013) Cardiomyocyte proliferation and progenitor cell recruitment underlie therapeutic regeneration after myocardial infarction in the adult mouse heart. EMBO Molecular Medicine 5: 191-209

Oh H, Bradfute SB, Gallardo TD, Nakamura T, Gaussin V, Mishina Y, Pocius J, Michael LH, Behringer RR, Garry DJ et al (2003) Cardiac progenitor cells from adult myocardium: Homing, differentiation, and fusion after infarction. Proc Natl Acad Sci USA 100: 12313-12318

Palacios JA, Schneider MD (2013) Heart to heart: grafting cardiosphere-derived cells augments cardiac self-repair by both myocytes and stem cells. EMBO Mol Med 5: 177-179

Qian L, Huang Y, Spencer CI, Foley A, Vedantham V, Liu L, Conway SJ, Fu J-d, Srivastava D (2012) In vivo reprogramming of murine cardiac fibroblasts into induced cardiomyocytes. Nature 485: 593-598

Senyo SE, Steinhauser ML, Pizzimenti CL, Yang VK, Cai L, Wang M, Wu T-D, Guerquin-Kern J-L, Lechene CP, Lee RT (2013) Mammalian heart renewal by pre-existing cardiomyocytes. Nature 493: 433-436

Sturzu AC, Wu SM (2011) Developmental and Regenerative Biology of Multipotent Cardiovascular Progenitor Cells. Circ Res 108: 353-364

Wong SCY, Fukuchi M, Melnyk P, Rodger I, Giaid A (1998) Induction of Cyclooxygenase-2 and Activation of Nuclear Factor-{kappa}B in Myocardium of Patients With Congestive Heart Failure. Circulation 98: 100-103

Wu SM, Fujiwara Y, Cibulsky SM, Clapham DE, Lien C-l, Schultheiss TM, Orkin SH (2006) Developmental Origin of a Bipotential Myocardial and Smooth Muscle Cell Precursor in the Mammalian Heart. Cell 127: 1137-1150

Main Text:
Page 5–7
PGE_2 regulates cardiac Sca-1$^+$ cells

Among the identified markers, Sca-1 is commonly expressed in various cardiac stem/progenitor cell populations (Oh et al, 2003; Sturzu & Wu, 2011). We therefore sought to investigate the effect of PGE_2 on stem cell-mediated cardiomyocyte replenishment by examining Sca-1$^+$ cell activities. Quantitative RT-PCR revealed that only Sca-1 expression peaked on day 3 post-MI and this level was further increased at the same time point upon PGE_2 treatment but was repressed by indomethacin (Supporting Information Fig 4). Examination of cardiac transcription factor expression suggested that Nkx2.5 (Wu et al, 2006) had similar expression pattern to that of Sca-1 at the infarct zone and the remote area (Supporting Information Fig 5). Furthermore, PGE_2 also elevated the expression of Nkx2.5 in Sca-1$^+$ cells (Supporting Information Fig 6).

Because tamoxifen injection in M/Z mice leads to conversion of β-Gal to GFP in cardiomyocytes, we thought to take this advantage to examine cardiomyogenic differentiation ability of the cardiac Sca-1$^+$ cells. The tamoxifen injection was given to the M/Z mice after MI surgery, and therefore, only α-MHC$^+$ cells would express GFP (Supplementary Information Fig 7A). This experiment allowed us to determine whether Sca-1$^+$ cells possess the ability to differentiate into α-MHC$^+$ cells. Following MI surgery and tamoxifen injection for 3 days, Sca-1$^+$/GFP$^+$ cells could be detected. The percentage of double positive cells was further increased upon PGE_2 treatment (Supplementary Information Fig 7B and C). In addition, Sca-1$^+$/α-MHC$^+$ cells were not observed before tamoxifen labeling and they do not arise from cardiomyocyte de-differentiation (Hsieh et al, 2007; Senyo et al., 2013) (Supplementary Information Fig 8). These results reveal the potential contribution of cardiac Sca-1+ stem/progenitor cells to cardiomyocyte replenishment after MI.

Following MI, M/Z system serves as a platform to assess the cardiomyocytes differentiated from endogenous stem/progenitor cells. To evaluate the cardiomyocyte differentiation ability of cardiac Sca-1$^+$ cells and the importance of PGE_2 pathway during this process, the cells were isolated from wild-type and EP2$^{-/-}$ mice (Kennedy et al, 1999) for intramyocardial injection after MI surgery (Loffredo et al, 2011; Supporting Information Fig 11A). The EP2$^{-/-}$ transgenic mouse was chosen due to the expression of this PGE_2 receptor was significantly induced in hearts after MI and in cardiac Sca-1$^+$ cells after PGE_2 treatment (Supporting Information Fig 9 and 10). Quantification of the GFP$^+$ and β-Gal$^+$ cardiomyocyte numbers revealed that injection of wild-type Sca-1$^+$ cells reduced both GFP$^+$ and β-Gal$^+$ cardiomyocyte numbers and that approximately 10% of the peri-infarct cardiomyocytes were GFP$^-$ and β-Gal$^-$, suggesting cardiomyocyte differentiation of the injected cardiac Sca-1$^+$ cells. In contrast, we did not observe such change in the M/Z mice receiving injection of EP2$^{-/-}$ Sca-1$^+$ cells (Fig. 2F, Supporting Information Fig 11). Together these results indicate that the PGE_2/EP2 signaling may regulate the ability of cardiac Sca-1$^+$ cells to differentiate into cardiomyocytes. Results from in vitro culture also provided evidence that the expression of Nkx2.5 and cTnT was evidently improved in isolated cardiac small cells and Sca-1$^+$ cells by PGE_2 (Supporting Information Fig 12B and C). Surprisingly, mature sarcomeric structure, as determined by the expression of cTnT, were seen in the cardiomyocyte-depleted small cells after PGE_2 treatment (Supporting Information Fig 12A and B), suggesting the ability of PGE_2 to augment cardiomyocyte differentiation.

Discussion:
Page 10

On the basis of Senyo's finding, several commentary articles have pointed out that the contribution of stem/progenitor cells to cardiac repair may be negligible (Mummery & Lee, 2013). Despite the use of ^{15}N labeling system, one question remains unsolved is the dilution of the GFP$^+$ cardiomyocyte pool in the M/Z mice after MI (Senyo et al, 2013). Results in our study and others have demonstrated that the number of cardiomyocytes replenished by endogenous

stem/progenitor cells at the infarct border zone is greater than the number of cells derived from the dividing pre-existing cardiomyocytes. Here, the results also reveal that the ability of stem/progenitor cells to give rise to cardiomyocytes could be modulated, suggesting a potential therapeutic application of the endogenous stem/progenitor cells for cardiac repair.

Figures and Supporting Information Figures:

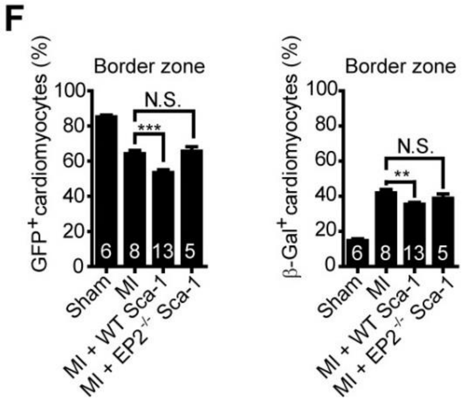

Figure 2. COX-2-dependent signaling pathway stimulates cardiomyocyte replenishment with endogenous stem/progenitor cells shortly after infarction.

*F. Following DAB staining, the percentages of GFP$^+$ and β-Gal$^+$ cardiomyocytes at the border zone of the young heart with or without cell injection after MI were quantified and statistically analyzed. Sample size is indicated in the bar chart. **p< 0.01, ***p< 0.001; N.S., not significant. Data are presented as the mean ± s.e.m.*

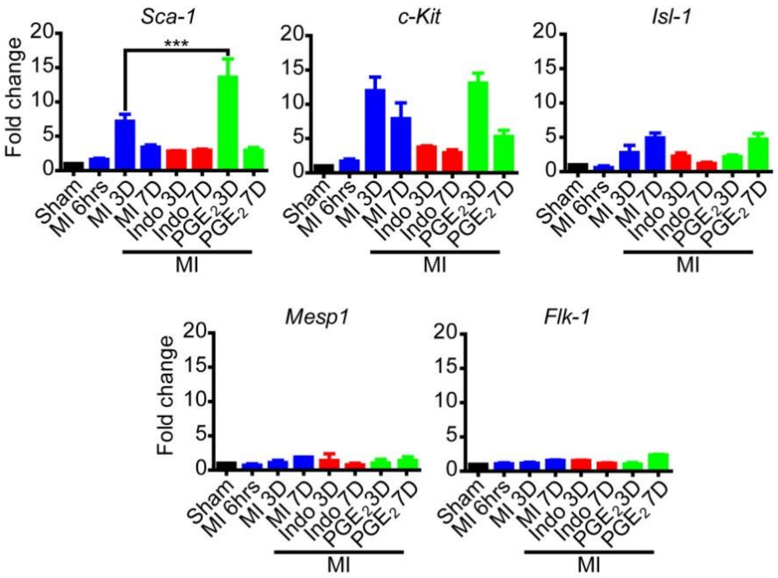

Supporting Information Fig 4. Gene expression of Sca-1 is the most responsive to PGE$_2$ treatment.

*Expression of the cardiac stem/progenitor marker genes in the infarcted region of injured hearts was analyzed by quantitative RT-PCR. The fold change is a relative quantification normalized to the sham control. ***p< 0.001. n ≥ 3. Data are presented as the mean ± s.e.m. Indo, Indomethacin; MI, myocardial infarction.*

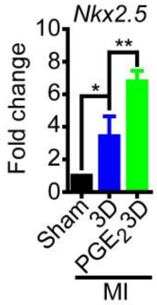

Supporting Information Fig 6. PGE$_2$ augments expression of Nkx2.5 in the Sca-1$^+$ cells of injured heart.

*At day 3 post-surgery, the Sca-1$^+$ cells from the heart treated with or without PGE$_2$ were isolated for quantitative RT-PCR analysis for Nkx2.5 expression. The fold change is a relative quantification normalized to the sham control. *p<0.05, **p<0.01. Data are presented as the mean ± s.e.m. MI, myocardial infarction.*

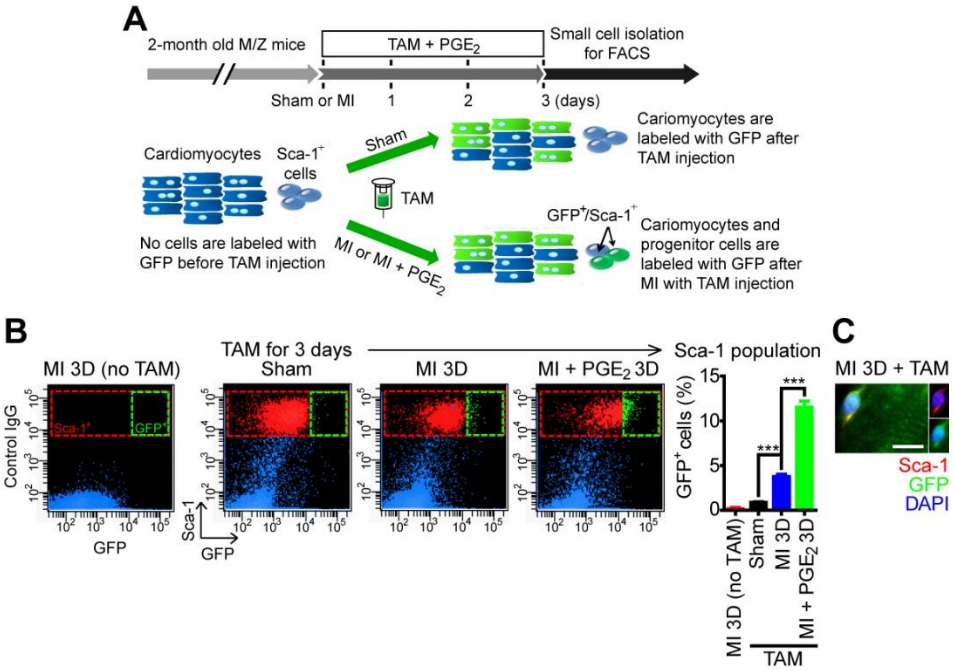

Supporting Information Fig 7. Examination of cardiomyogenic differentiation ability of cardiac Sca-1$^+$ cells after myocardial infarction.

A. *Schematic diagram depicting the experimental procedure. Following myocardial infarction (MI) surgery, the M/Z mice were injected with 80 µg/g tamoxifen (TAM) per day for 3 days with or without an additional PGE$_2$ treatment. The sham control was also treated with the same dosage of tamoxifen and PGE$_2$ simultaneously for 3 days. The cardiac small cells were isolated and subjected to flow cytometric analysis of Sca-1$^+$/GFP$^+$ cells at day 3 post-surgery.*
B. *The percentages of Sca-1$^+$/GFP$^+$ cells at day 3 post-MI were quantified by flow cytometry. Mice that did not receive tamoxifen injections after the MI surgery served as negative control (no TAM). The number of Sca-1$^+$/GFP$^+$ cells was also quantified. The data are presented as percentage calculated by dividing the number of double-positive cells by the total number of Sca-1$^+$ cells. ***p<0.001. Data are presented as the mean ± s.e.m. n≥ 4.*
C. *Prior to isolating the small cardiac cells for flow cytometry analysis, a small portion of the heart tissue was excised, fixed and immunostained. Scale bars, 10 µm.*

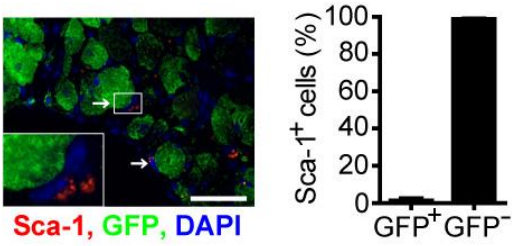

Supporting Information Fig 8. Sca-1+/α-MHC+ cells were not detected prior to tamoxifen labeling and these cells did not arise from cardiomyocyte de-differentiation.

Following 14 days of tamoxifen labeling, the animals were allowed to recover for 1 month prior to myocardial infarction (MI) surgery. At day 3 post-MI, the organ was collected for immunostaining analysis. Shown is a representative image of the Sca-1+ cells and the original magnifications are as indicated. The Sca-1+ cells with or without GFP+ signal were quantified. Scale bars, 50 µm. n =3. Data are presented as the mean ± s.e.m.

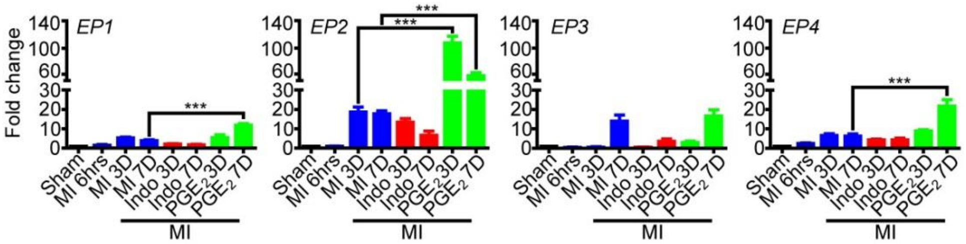

Supporting Information Fig 9. PGE$_2$ increases the expression of EP2 receptor after myocardial infarction.

*The expression of PGE$_2$ receptors, EP1, 2, 3 and 4, in response to different drug treatments at the infarct region of the injured heart was examined by quantitative RT-PCR. The fold change is a relative quantification normalized to the sham control. ***p<0.001. Data are presented as mean ± s.e.m. Indo, Indomethacin; MI, myocardial infarction.*

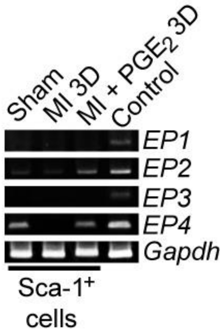

Supporting Information Fig 10. The expression of EP2 receptor increases in Sca-1+ cells isolated from the infarcted heart after PGE$_2$ treatment.

Semi-quantitative PCR was performed to examine the expression of PGE$_2$ receptors, EP1, 2, 3 and 4, in cardiac Sca-1+ cells isolated after myocardial infarction (MI). Un-sorted cardiomyocyte-depleted small cells from the sham group served as positive control.

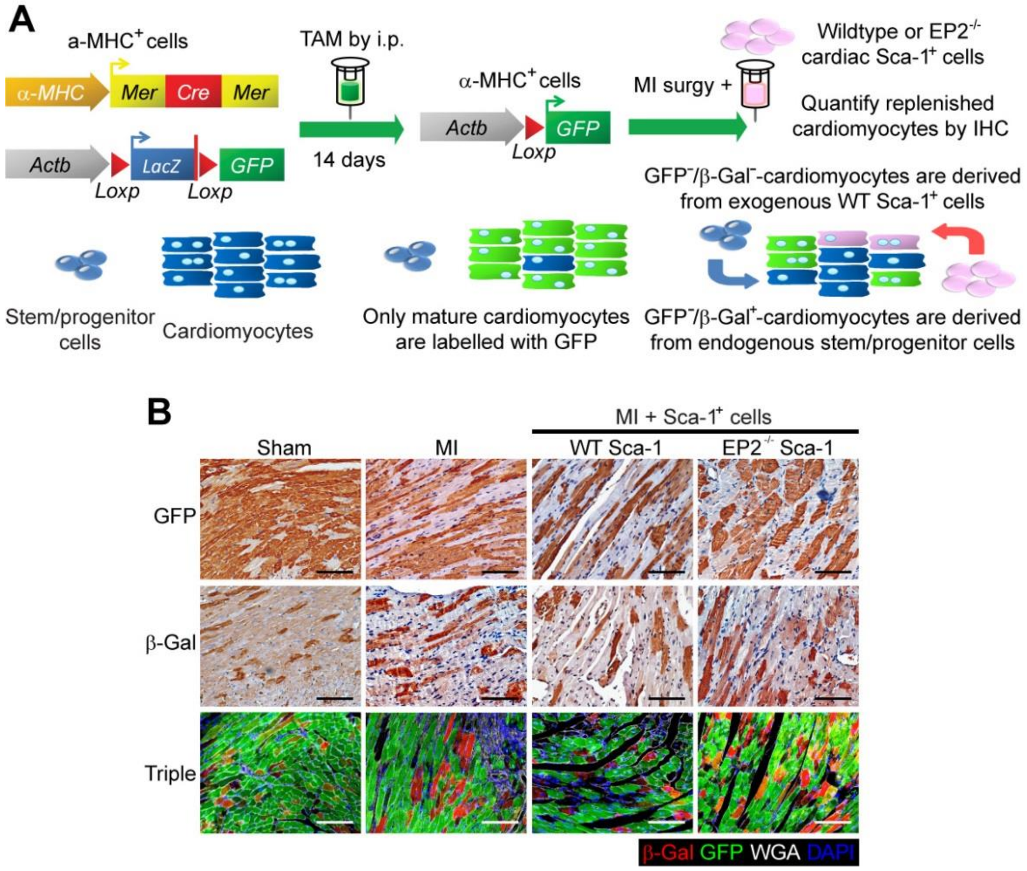

Supporting Information Fig 11. Quantification of the degree of cardiomyocyte replenishment after Sca-1$^+$ cell injection in injured hearts.

A. *Schematic diagram depicting the experimental procedure. Following 14 days of tamoxifen injection, the M/Z mice were injected intramyocardially with wild-type or EP2 knockout (EP2$^{-/-}$) cardiac Sca-1$^+$ cells after myocardial infarction (MI). The hearts were harvested at day 14 post-MI for examination.*
B. *At day 14 post-infarction, the hearts were harvested for DAB and immunofluorescence triple staining to examine the GFP$^+$ or β-Gal$^+$ cardiomyocytes. Shown are representative images from each group. Scale bars, 100 μm.*

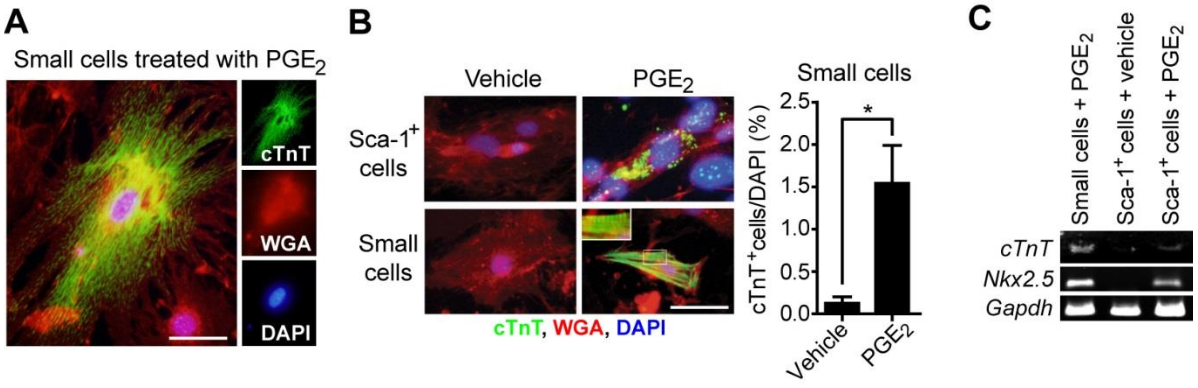

Supporting Information Fig 12. In vitro differentiation ability of isolated cardiac cells into cardiomyocytes.

A. *The cardiomyocyte-depleted small cells were allowed to attach for 3 days followed by PGE_2 treatment (10 μM) for another 3 days. Immunocytochemistry was performed to examine cardiomyocyte differentiation, as determined by the expression of the cardiomyocyte marker cardiac troponin T (cTnT) at day 10. The membrane and nucleus were stained with the membrane dye, WGA, and DAPI, respectively. Scale bar, 50 μm.*

B. *Under the same culture condition, the cardiac Sca-1$^+$ cells and small cells subjected to vehicle or PGE_2 treatment were stained with cTnT for sarcomeric structure analysis. The percentage of small cells with mature sarcomeric structures following vehicle or PGE_2 treatment was quantified. Scale bar, 50 μm. *p< 0.05. Data are presented as mean ± s.e.m.*
C. *Following the same culture procedure, the effect of PGE_2 on the expression of cardiac marker genes, Nkx2.5 and cTnT, in Sca-1$^+$ cells was analyzed by semi-quantitative PCR. Small cells treated with PGE_2 serve as positive control.*

2) How many fields and cells have the authors counted? From the sections in the figure one can see that the authors selected sections with cardiomyocytes sectioned vertical as well as horizontal. Also staining intensity is very different?

We thank the Reviewer for these questions. To quantify cardiomyocytes, 3 sections from each heart, including 2 sections from the infarction border zone and 1 from the remote area were analyzed at a magnification of 200x with light microscopy. Cells with visible sarcomere structures were analyzed and the average number of cells counted was 171.8 ± 5.8 per photo image. Because the cardiomyocytes align in three different directions in the heart, they are oriented differently when tissue sections are observed under low magnification. Regarding variation in staining intensity, the images were examined under higher magnification. The DAB signal intensity in different areas of the heart is indistinguishable (Supporting Information Fig 1). We therefore suspect that variation of the DAB intensity from region to region may be due to the difference in magnification.

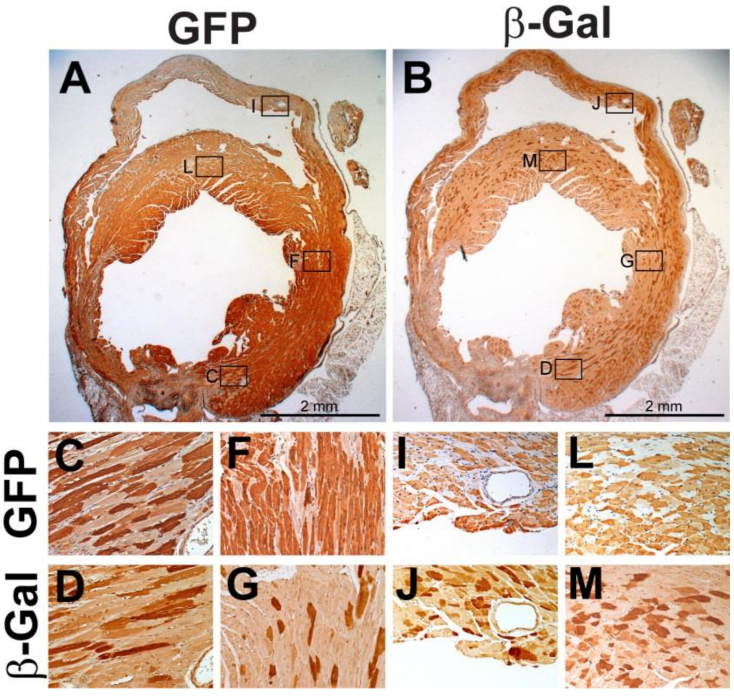

Supporting Information Fig 1. Distribution of GFP$^+$ or β-Gal$^+$ cardiomyocytes in the heart at day 14 after myocardial infarction.

A-B. The heart at day 14 post-infarction was subjected to GFP or β-Gal staining. The images of whole heart cross-sections were taken at low magnification.

C-H. The specified images are taken from the border zone, remote area of the scar site, the interventricular septum and the right ventricle under higher magnification.

3) Overall, all differences shown are significant but minor. Also the improvement of the ejection fraction is with around 5% much lower than many other published strategies. Thus, the importance and future impact of PGE2 remains unclear.

We thank the Reviewer for the comments. Our results suggest that the stem/progenitor cells, not the pre-existing cardiomyocytes, are the dominant population targeted by PGE_2. Therefore, we speculate that the improvement in cardiac function would not be as profound as drug or cell therapies aimed at protecting cardiomyocytes from apoptosis. Furthermore, two studies using different cell therapies to augment stem/progenitor cell-dependent cardiomyocyte repopulation have also demonstrated around 40% of improved cardiac function, which is consistent with our finding (Loffredo et al, 2010; Malliaras et al, 2013). Most importantly, PGE_2 treatment does not require transplantation of exogenous cells.

References:

Loffredo Francesco S, Steinhauser Matthew L, Gannon J, Lee Richard T (2011) Bone Marrow-Derived Cell Therapy Stimulates Endogenous Cardiomyocyte Progenitors and Promotes Cardiac Repair. Cell Stem Cell 8: 389-398

Malliaras K, Zhang Y, Seinfeld J, Galang G, Tseliou E, Cheng K, Sun B, Aminzadeh M, Marbán E (2013) Cardiomyocyte proliferation and progenitor cell recruitment underlie therapeutic regeneration after myocardial infarction in the adult mouse heart. EMBO Molecular Medicine 5: 191-209

4) Ejection fraction after 1 month is not improved by PGE2 but after 2 months. What is the possible mechanism if the authors have shown in Fig. 1 that the window of progenitor-cell mediated cardiomyocyte replenishment is from post MI day 7 to 10? Does this not indicate that the PGE2-mediated effect is stem-cell independent? And how do the increased IL10 levels at day 3 post MI fit?

We thank the Reviewer for raising these important questions. In the absence of PGE_2 treatment, we observed worse cardiac function of MI heart at 2 months than at 1 month, likely due to an increase in infarct lesion and the number of dead cardiomyocytes over time after the heart injury. Following PGE_2 treatment, repopulation of cardiomyocytes by stem/progenitor cells is increased by ~9%. We therefore speculate that these stem/progenitor cell-derived cardiomyocytes not only contribute to repopulation of lost cardiomyocytes but also ameliorate the degree of infarct lesion over time. However, the exact time when an improvement in cardiac function is expected following stem/progenitor cell-modulated cardiomyocyte repopulation remains unclear. Loffredo *et al*. demonstrated that cardiac function was improved by recovered ability for endogenous stem/progenitor cells to repopulate cardiomyocytes at 2 months post-MI following cell injection-based therapy, which is consistent with our findings (Loffredo et al, 2010). These findings suggest that stem/progenitor cell-depend cardiomyocyte replenishment is a slow but effective way to ameliorate heart function.

The role of IL-10-secreting M2 macrophages in tissue regeneration has been reported (Nemeth et al, 2009). Quantification of *Sca-1* expression reveals that its level peaked on day 3 post-MI (Supporting Information Fig 4). Furthermore, *in vitro* culture provides evidence that the microenvironment is important for cardiomyocyte differentiation of cardiac stem/progenitor cells (Supporting Information Fig 12). More importantly, our data support the role of early inflammatory response, which is activated within 7 days post-MI, in regulating cell regeneration and demonstrate that PGE_2 could modulate the inflammatory microenvironment to promote cardiomyocyte replenishment by increasing

M2 macrophages, hence the level of *IL-10*. Because cardiomyocyte replenishment is saturated at day 10 after injury, an increase in level of IL-10 at day 3 post-MI would fit.

References:

Loffredo Francesco S, Steinhauser Matthew L, Gannon J, Lee Richard T (2011) Bone Marrow-Derived Cell Therapy Stimulates Endogenous Cardiomyocyte Progenitors and Promotes Cardiac Repair. Cell Stem Cell 8: 389-398

Nemeth K, Leelahavanichkul A, Yuen PST, Mayer B, Parmelee A, Doi K, Robey PG, Leelahavanichkul K, Koller BH, Brown JM et al (2009) Bone marrow stromal cells attenuate sepsis via prostaglandin E2-dependent reprogramming of host macrophages to increase their interleukin-10 production. Nat Med 15: 42-49

5) Is the ejection fraction for PGE2 treated animals at 2 month significantly higher than for MI or PGE2-treated animals at 1 month?

We thank the Reviewer for noting this question. Statistical analysis shows that the cardiac function of PGE_2 treated animals at 2 months is not significantly higher than that for MI or PGE_2-treated animals at 1 month.

6) The authors have shown interesting and intriguing data in vivo. However, those data are often difficult to interpret and as mentioned above it is clear if data are only correlated or are depended on each other. Thus, it is mandatory, that the authors show in cell-based assays that PGE2 has a significant effect on progenitor cell differentiation.

We thank the Reviewer for raising this concern. To identify the stem cell population responsive to the PGE_2 treatment, we performed quantitative RT-PCR to analyze the expression of several stem cell marker genes. Of all of the genes analyzed, the expression of *Sca-1* was the most significantly enhanced by PGE_2 (Supporting Information Fig 4). Following the isolation of Sca-1$^+$ cells and cardiomyocyte-depleted small cells from young mice, the cells were cultured on fibronectin-coated plates for 3 days, as it took at least 3 days for the cells to attach (Oh et al, 2003). After attachment, cells were treated with PGE_2 for another 3 days and their cardiomyocyte differentiation potential examined at day 10. Interestingly, although we observed *cTnT* expression in the Sca-1$^+$ cells treated with PGE_2, the sarcomeric structure was immature (Supporting Information Fig 12B and C). Surprisingly, mature sarcomeric organization was seen in the cardiomyocyte-depleted small cells after PGE_2 treatment (Supporting Information Fig 12A and B). Taken together, PGE_2 may direct a purified cardiac stem cell population to differentiate into cardiomyocytes *in vitro.*

Reference:

Oh H, Bradfute SB, Gallardo TD, Nakamura T, Gaussin V, Mishina Y, Pocius J, Michael LH, Behringer RR, Garry DJ et al (2003) Cardiac progenitor cells from adult myocardium: Homing, differentiation, and fusion after infarction. Proc Natl Acad Sci USA 100: 12313-12318

Supporting Information Figures:

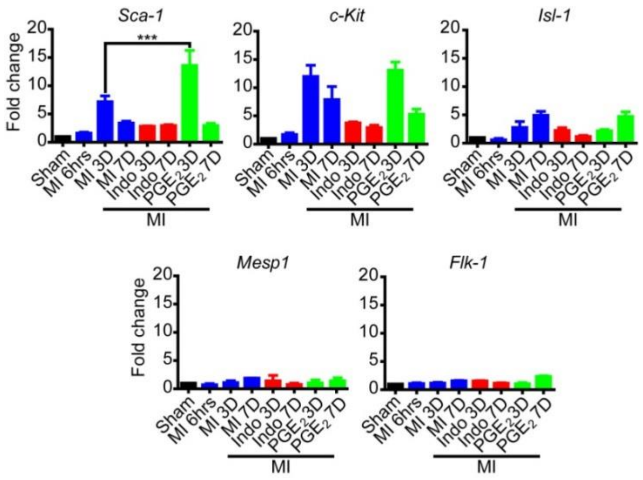

Supporting Information Fig 4. Expression of Sca-1 gene is most responsive to PGE_2 treatment.

*Expression of the cardiac stem/progenitor marker genes in the infarcted regions of injured hearts was analyzed by quantitative RT-PCR. The fold change is a relative quantification normalized to the sham control. ***$p< 0.001$. $n \geq 3$. Data are presented as the mean ± s.e.m. Indo, Indomethacin; MI, myocardial infarction.*

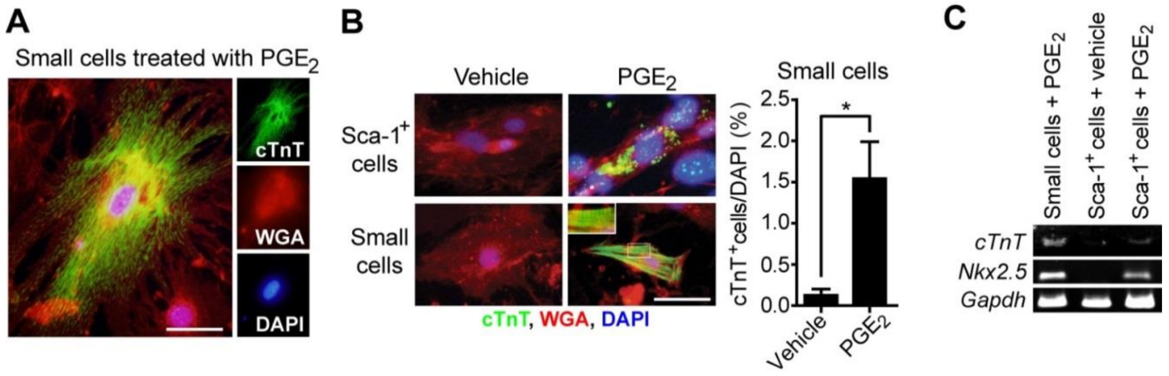

Supporting Information Fig 12. Cardiomyocyte differentiation ability of isolated cardiac cells in vitro.

A. *The cardiomyocyte-depleted small cells were allowed for attachment for 3 days, followed by PGE_2 treatment (10μM) for another 3 days. Immunocytochemistry was performed to examine cardiomyocyte differentiation, as determined by the expression of the cardiomyocyte marker cardiac troponin T (cTnT) on day 10. The membrane and nucleus were stained with membrane dye WGA and DAPI, respectively. Scale bar, 50 μm.*
B. *Under the same culture condition, the cardiac Sca-1^+ and small cells subjected to vehicle or PGE_2 treatment were stained for cTnT for sarcomeric structure analysis. Quantification results present the percentage of small cells co-stained with mature sarcomeric structure following vehicle or PGE_2 treatment. Scale bar, 50 μm. *$p< 0.05$. Data are presented as mean ± s.e.m.*
C. *Following the same culture procedure, the effect of PGE_2 on expression of cardiac marker genes Nkx2.5 and cTnT in Sca-1^+ cells was analyzed by semi-quantitative PCR. Small cells treated with PGE_2 serve as positive control.*

7) According to the authors data the progenitor cells differentiate within 7 to 10 days to adult cardiomyocytes. Is the reviewer's interpretation correct? Or can progenitor cell-derived cardiomyocytes distinguished? Considering development, in vitro stem cell differentiation assays and neonatal cardiomyocyte differentiation assays in 3D this is extremely fast.

We thank the Reviewer for raising this important issue. We are also surprised at the fact that stem/progenitor cell-dependent cardiomyocyte replenishment is initiated within 7 days and saturated at day 10 post-MI in mice (Figure 1). We provide evidence that the key signal regulating stem/progenitor cells is activated within 5 days after MI (Figure 2) and that the stem/progenitor cell marker gene *Sca-1* expression peaks at day 3 post-MI following PGE_2 treatment (Supporting Information Fig 4). We speculate that the cardiac stem/progenitor cells are activated by the inflammatory signal at day 3 post-MI and then undergo cardiomyogenic differentiation in the following days until day 10. This is also confirmed by *in vitro* cardiac small cell culture results (Supporting Information Fig 12). Accumulating studies have pointed out that cardiomyocyte differentiation of endogenous or exogenous stem cells, including c-Kit$^+$, Sca-1$^+$ and Wt-1$^+$ cells in adult mice takes place within 14 days after injury or cell transplantation (Fransioli et al, 2008; Mouquet et al, 2005; Oh et al, 2003; Smart et al, 2011). Using the same *in vivo* system, Loffredo et al. also observed that GFP$^+$ cardiomyocyte pool is diluted by 10% at day 14 post-MI (Loffredo et al, 2011). In addition, *in vitro* differentiation of ES cells to cardiomyocytes by hanging drop method also requires 7 days (Fuegemann et al, 2007; Guan et al, 2007; Sachinidis et al, 2003). In sum, we speculate that the inflammatory pathway stimulated within a short period of time after MI is the key to drive stem cell differentiation into cardiomyocytes. We believe that the presence of cardiac stem/progenitor-derived cardiomyocytes at 10 days post-MI is within reasonable time frame. Although we do not exclude the possibility that more cardiomyocytes are regenerated after 10 days, the number of newly formed cardiomyocytes may be so small that it is undetectable at the border zone using our system.

References:

Fransioli J, Bailey B, Gude NA, Cottage CT, Muraski JA, Emmanuel G, Wu W, Alvarez R, Rubio M, Ottolenghi S et al (2008) Evolution of the c-kit-Positive Cell Response to Pathological Challenge in the Myocardium. Stem Cells 26: 1315-1324

Guan K, Wagner S, Unsöld B, Maier LS, Kaiser D, Hemmerlein B, Nayernia K, Engel W, Hasenfuss G (2007) Generation of Functional Cardiomyocytes From Adult Mouse Spermatogonial Stem Cells. Cir Res 100: 1615-1625

Loffredo Francesco S, Steinhauser Matthew L, Gannon J, Lee Richard T (2011) Bone Marrow-Derived Cell Therapy Stimulates Endogenous Cardiomyocyte Progenitors and Promotes Cardiac Repair. Cell Stem Cell 8: 389-398

Mouquet F, Pfister O, Jain M, Oikonomopoulos A, Ngoy S, Summer R, Fine A, Liao R (2005) Restoration of Cardiac Progenitor Cells After Myocardial Infarction by Self-Proliferation and Selective Homing of Bone Marrow-Derived Stem Cells. Circ Res 97: 1090-1092

Oh H, Bradfute SB, Gallardo TD, Nakamura T, Gaussin V, Mishina Y, Pocius J, Michael LH, Behringer RR, Garry DJ et al (2003) Cardiac progenitor cells from adult myocardium: Homing, differentiation, and fusion after infarction. Proc Natl Acad Sci USA 100: 12313-12318

Sachinidis A, Fleischmann BK, Kolossov E, Wartenberg M, Sauer H, Hescheler J (2003) Cardiac specific differentiation of mouse embryonic stem cells. Cardiovasc Res 58: 278-291

Smart N, Bollini S, Dube KN, Vieira JM, Zhou B, Davidson S, Yellon D, Riegler J, Price AN, Lythgoe MF et al (2011) De novo cardiomyocytes from within the activated

adult heart after injury. Nature 474: 640-644

3rd Editorial Decision 26 August 2013

Thank you for the submission of your research manuscript to our editorial office. We have now received the enclosed reports on it. As you will see, referees #1, 2 and 3 remain negative about the study and only the novel referee (#4) supports publication in its current form.

Although the concerns raised by referee #3 have been mostly addressed, those from referees #1 and 2 have not been and these referees still raise serious issues regarding the conclusiveness of the data pinpointing important technical issues that preclude a solid interpretation of the experimental evidence provided. Following extensive discussion at the editorial level (together with my colleague, our Chief Editor and the head of publication), we decided to seek further advice (Referee #4). Unfortunately, the outcome of this was not positive due the nature of the criticisms, and the amount of work likely to be required to convincingly address them on a second revision.

As you certainly know, EMBO Molecular Medicine can only invite revision of papers that receive enthusiastic support from a majority of referees, I am afraid that we do not feel it would be productive to call for a revised version of your manuscript at this stage and therefore we cannot offer to publish it.

Given the potential interest of the findings, however, we would have no objection to consider a new manuscript on the same topic if at some time in the near future you obtained data that would considerably strengthen the message of the study and address the referees concerns in full. To be completely clear, however, I would like to stress that if you were to send a new manuscript this would be treated as a new submission rather than a revision and would be reviewed afresh, in particular with respect to the literature and the novelty of your findings at the time of resubmission. If you decide to follow this route, please make sure you nevertheless upload a letter of response to the referees' comments.

At this stage of analysis, though, I am sorry to have to disappoint you. I nevertheless hope, that the referees' comments will be helpful in your continued work in this area and I thank you for considering EMBO Molecular Medicine.

***** Reviewer's comments *****

Referee #1 (Comments on Novelty/Model System):

critical genetic mouse experiments are missing

Referee #1 (Remarks):

I have reviewed the entire lengthy rebuttal and find the response not convincing.

In particular, the response to the concerns raised by the other two reviewers about why functional improvement takes 2 months, whereas the PGE2-stimulated differentiation of sca1-stem cells happens on a scale of days, is not clear. I also do not find the PGE2-receptor knockout experiment convincing as it is a germline knockout. It would be more convincing if this experiment were done in combination with a cardiomyocyte-specific knockout.

In response to the criticisms that I have raised previously (which were similar to a criticism by reviewer #3): The new line of argumentation that is provided to justify the alpha-MHC-MerCreMer, Z/EG approach is not convincing. The labeling efficiency is irrelevant so as long as it is reproducibly constant. 30% labeling is sufficient for doing this experiment since it is about dilution/change of this percentage. In fact, a n y labeling efficiency is ok so as long as it is relatively constant and reproducible.

It may well be that the Z/EG mouse has advantages over the Z/AP mice, but cancer development was not reported in the original paper. In addition, there may be disadvantages to using the Z/EG approach as GFP has been shown to affect the fitness of cells, especially of cardiomyocytes.

© EMBO

A recurring discussion between the authors and the reviewers seems to be about the level of evidence that is required to justify the claim that PGE2 stimulates cardiomyogenesis from Sca1-progenitor cells. There are two genetic experiments to support this notion:
1.) use an inducible and cardiomyocyte-specific Sca1-Cre line for lineage tracing.
2.) Use the same inducible and cardiomyocyte-specific Sca1-Cre line for knocking out the PGE2-receptor in these cells.

In summary, this reviewer cannot revoke the earlier assessment that this manuscript should not be published by EMBO Mol Med because critical experiments to support the claims are missing.

Referee #2 (Comments on Novelty/Model System):

Although many new experiments are included in the revised ms, the flow cytometry data is still suboptimal suggesting the authors lack basic technical knowledge of flow cytometry.

Referee #2 (Remarks):

The authors conducted a number of new experiments that addressed most of the reviewers' concerns. One disappointment is that their flow cytometry data is not high quality, superficial and poorly conducted, limiting conclusions about their M1/M2 conclusions.
Specific comments on the authors' responses:
1a. Satisfactory.
1b. Satisfactory. However, supplementary figure 12b should also include a histogram summarizing the cardiomyogenic potential of Sca1+ cells vs 'small cells' following PGE2 treatment. The Sca1+ cell data for cTnT+ cells is absent.
1c. Satisfactory.
1d. As stated in previous comments, F4/80 and Gr-1 and/or CD206 must be examined by first gating upon CD45+CD11b+ cells. This has not been conducted (nothing stated in figure legend or Materials and Methods) and the authors have merely stated vaguely that 'appropriate antibodies' have been used. As they are shown, the experiments have not been conducted to a satisfactory standard and are therefore not reliable.
1e-2d.Satisfactory.
2e. Not satisfactory. The flow cytometry experiment has not been performed as suggested. Therefore, no clear conclusions can be drawn from this experiment.
2f-2h. Satisfactory.

Referee #3 (Comments on Novelty/Model System):

Overall, the authors have tried hard to answer the issues raised. However, in my opinion the data are still inconclusive and in part contradictory. Finally, the proposed treatment has if at all a minor effect on cardiac function.

Referee #3 (Remarks):

Comment 1:
It is still unclear to this reviewer how a stem cell population contributes 10% new cardiomyocytes. In the paper by Senyo et al. only 3.2% new cardiomyocytes were detected in a time frame of 8 weeks. Even if there is a partial contribution of stem cells it is hard to explain how stem cells generate 10 % new cardiomyocytes.

Assuming the main contribution is from Sca1+ cells it is still unclear how this is possible if the population of Sca1+ cells is only increased 3 days post-MI but already decreased at day 7 post MI? Even more surprising is that these cells should already be differentiated at 3 days post MI to be GFP-positive. This means the cells need to differentiate in 2 days to activate the promoter to produce GFP that it reaches a protein level that can be detected at day 3 post MI. It appears rather

© EMBO

likely that these observations are due to cell fusion which has not yet been excluded. Also the in vitro differentiation assays suggest that Sca1+ cannot properly differentiate into cardiomyocytes, even after 10 days. And regarding the small cell population only 1.5% show some kind of differentiation.

A differentiation in vivo has still not been demonstrated.

Finally the authors perform studies utilizing EP2. How do the authors explain that EP2 is highly elevated at day 7 post MI in the Sca1+ cells in the PGE2 group (60-fold) when the number of Sca1+ cells is dramatically reduced back to the number in the control group?

Comment 2: ok

Comment 3:
The goal of biomedical research is to identify novel strategies to treat disease. Several approaches have already demonstrated to have a better potential to treat heart disease than the present study and many of these studies utilize growth factors, drugs or other strategies that do not require the transplantation of exogenous cells (e.g. miR approach, Neuregulin). Finally, this author would expect that the generation of 10% new cardiomyocytes should have a more profound effect on cardiac function.

Comment 4:
The answer by the authors further suggests to this reviewer that PGE2 has a stem cell independent effect.

Comment 5:
If the ejection fraction in PGE2-treated animals at 2 month is not significantly higher than the MI group after 1 month than the treatment appears to have only a protective effect and not a regenerative effect? This suggests again that PGE2 might act stem cell independent?

On the other hand a protective effect is surprising as PGE2 treatment is stopped after 14 days (?). It might be that the stem cells continue to differentiate into a mature phenotype to compensate a continuous loss of cardiomyocytes. However, overall, the effect of this treatment on cardiac function appears negligible.

Comment 6:
The characterisation of cardiac differentiation needs to be expanded.

Comment 7:
See answers to previous comments.

Referee #4 (Remarks):

I have been asked to comment on the authors' response to previous reviewer concerns.

Referee 1

In my view, the reviewer places excessive emphasis on 'contradictory' results between Hsieh Nat Med 2007 and the same group's 2012 Nature study using the same aMHC-MerCre Mer fate-mapping reagents. Keep in mind, the increase in 15N+ myocytes in Lee's paper was far too small to account for the loss of GFP+ ones. The latter data simply do not support the much-cited conclusion that myocyte proliferation predominates in self-repair, and I would not consider stable isotope imaging to be 'more definitive' than fate-mapping until this discrepancy is reconcilted and reproducibility between labs is amply demonstrated.

Conversely, the present reviewer's objection to pulse-chase experiments as misleading can be addressed by a number of precautions, including use of a bistable switch. In the present manuscript, for instance, the observed increase in b-Gal+ myocytes excludes any possibility that loss of GFP

© EMBO

was just an artifact of transgene silencing. Work of Malliaras and Marban, recently reported in this journal, give further credibility to the fate-mapping approach.

I am satisfied that Hsueh et al have taken a prudent and balanced approach on this central question of experimental strategy.

Referee 2

The authors have performed the suggested direct comparison of Celecoxib at early vs late intervals after infarction, and the new results confirm their conclusion.

The authors have performed the suggested direct test that PGE2 acts directly on progenitor/stem cells. The new cell culture studies show that PGE2 induces cTnT in purified Sca-1+ cells, though the effect was more striking in a heterogeneous cardiomyocyte-depleted small cell population. The latter result is not fully explained (which cell types are the preferred target?), but this would require much additional work. More importantly, and much more easily addressed, the former result is limited to just cTnT.

To test directly the mechanism for PGE2's effect, the authors have shown that injected cardiac Sca-1 cells lacking EP2 (the most relevant PGE2 receptor) do not participate in cardiomyocyte repletion. This is a very important addition but there are two issues with the Supplemental Figure 11. In the cartoon, the GFP+ cells should be described as "differentiated" cardiomyocytes, not necessarily "mature" ones. They might well be mononucleated and proliferation-competent. Also, data in the montage must be quantitated and significance shown, as done routinely for other figures.

The authors have performed the suggested additional studies of M1 and M2 macrophages, and the improved results support their conclusion.

Overstatements and caveats have been corrected.

Referee 3

Over-reliance on Senyo is discussed above.

Details of the quantitation have been provided.

Additional comments

I would tone down the claims in the Abstract ('cardiac Sca-1+ cells are the major PGE2-responsive population'), based on Suppl Fig 4 showing induction of Sca-1 mRNA by PGE2, since this experiment doesn't distinguish an expansion of the population vs higher levels in the cells already expressing Sca-1 vs inducing ectopic expression in Sca-1-negative cells. Minimally, FACS would be needed in addition.

"no pharmacological treatment is known to promote cardiomyocyte regeneration after injury" - What about thymosin b4 (Smart, Nature 2011), nerve growth factor (Lam PLoS One 2012), nanofiber scaffolds with VEGF (Lin, Sci Transl Med. 2012)?

I would also restate the conclusion that "blocking the inflammatory reaction with COX-2 inhibitors reduces the capability of endogenous stem/progenitor cells to repopulate lost cells." The authors' evidence from cell culture and from grafting EP2-null cardiac Sca-1 cells indicate a direct effect of PGE2 on the progenitor cells, unrelated to inflammation. There may or may not be functional importance to the change in M2 macrophages, but these two experiments in the present report show the change in endogenous stem/progenitor cells does not need to involve the inflammatory partners.

© EMBO

Resubmission 18 November 2013

Referee #1:

1. I have reviewed the entire lengthy rebuttal and find the response not convincing. In particular, the response to the concerns raised by the other two reviewers about why functional improvement takes 2 months, whereas the PGE2-stimulated differentiation of sca1-stem cells happens on a scale of days, is not clear. I also do not find the PGE2-receptor knockout experiment convincing as it is a germline knockout. It would be more convincing if this experiment were done in combination with a cardiomyocyte-specific knockout.

We thank the reviewer for raising these points. Loffredo et al. demonstrated that cardiac function was improved by recovering the ability of endogenous stem/progenitor cells to repopulate cardiomyocytes at 2 months post-MI following cell therapy, which is consistent with our findings (Loffredo et al, 2010). In comparison with treatments aiming to protect cardiomyocytes against apoptosis, our findings suggest that stem/progenitor cell-dependent cardiomyocyte replenishment is a slow but effective way to ameliorate heart function. Our results have implicated that PGE_2 exerts its cardiac repair function by modulating both stem cell activity and inflammatory micro-environment. However, further examinations to optimize the dosage and delivery method of PGE_2 are necessary to dissect the underlying mechanism for such regulations. Therefore, we feel that the data of cardiac function examination may be too preliminary and have removed the data from the resubmitted manuscript. On the other hand, we observed that EP2 is the most responsive receptor following PGE_2 treatment, and may be involved in the regulation of stem cell activities. Therefore cardiomyocyte-specific EP2 knockout transgenic mouse may not be a suitable model to examine the role of EP2 on stem cells because its expression is only depleted in cardiomyocytes but not stem cells.

Reference:

Loffredo Francesco S, Steinhauser Matthew L, Gannon J, Lee Richard T (2011) Bone marrow-derived cell therapy stimulates endogenous cardiomyocyte progenitors and promotes cardiac repair. Cell Stem Cell 8: 389-398

2. In response to the criticisms that I have raised previously (which were similar to a criticism by reviewer #3): The new line of argumentation that is provided to justify the alpha-MHC-MerCreMer, Z/EG approach is not convincing. The labelling efficiency is irrelevant so as long as it is reproducibly constant. 30% labelling is sufficient for doing this experiment since it is about dilution/change of this percentage. In fact, a n y labelling efficiency is ok so as long as it is relatively constant and reproducible.

We thank the reviewer for these comments; however, we do not agree that any labelling efficiency is sufficient for a fate-mapping study. In particular, in our study it would be too difficult to distinguish between the regenerated β-Gal^+ cardiomyocytes and the pre-existing ones because the majority of cells would remain β-Gal^+ if the labelling efficiency is quite low. Therefore, despite the percentage of labelled cells is changed or diluted, it will be difficult to conclude whether an increase in β-Gal^+ cardiomyocytes attributes to pre-existing cell proliferation or to differentiation of stem cells. In consistent with our result, 80% of labelling efficiency was also achieved in Senyo's study. Such labelling efficiency is also important to define which cell population, pre-existing cardiomyocytes or stem cells, is dominant for cardiomyocyte regeneration. Reviewer #4 has also pointed out that the sample size of ^{15}N labelled cells examined in Senyo's study was too small and further analyses will be required before the conclusion could be drawn. A recent study reported by Ellison et al. has demonstrated that cardiac c-Kit^+ cells have the ability to give rise to cardiomyocytes (Ellison et al. 2013). Interestingly, the M/Z system was also used to evaluate cardiac regeneration ability. In addition, M/Z system was combined with BrdU labelling to show the majority of proliferating cardiomyocytes express β-Gal (Malliaras et al. 2013). In comparison to the immunohistochemistry-based cell quantification in Senyo's study, both studies have employed flow cytometry for more

sophisticated cell counting. These studies support our result that β-Gal^{+} cardiomyocyte percentage could be modulated.

References:

Ellison Georgina M, Vicinanza C, Smith Andrew J, Aquila I, Leone A, Waring Cheryl D, Henning Beverley J, Stirparo Giuliano G, Papait R, Scarfò M et al (2013) Adult c-kitpos cardiac stem cells are necessary and sufficient for functional cardiac regeneration and repair. Cell 154: 827-842

Malliaras K, Zhang Y, Seinfeld J, Galang G, Tseliou E, Cheng K, Sun B, Aminzadeh M, Marbán E (2013) Cardiomyocyte proliferation and progenitor cell recruitment underlie therapeutic regeneration after myocardial infarction in the adult mouse heart. EMBO Mol Med 5: 191-209

Senyo SE, Steinhauser ML, Pizzimenti CL, Yang VK, Cai L, Wang M, Wu T-D, Guerquin-Kern J-L, Lechene CP, Lee RT (2013) Mammalian heart renewal by pre-existing cardiomyocytes. Nature 493: 433-436

3. A recurring discussion between the authors and the reviewers seems to be about the level of evidence that is required to justify the claim that PGE2 stimulates cardiomyogenesis from Sca1-progenitor cells. There are two genetic experiments to support this notion:
1.) use an inducible and cardiomyocyte-specific Sca1-Cre line for lineage tracing.
2.) Use the same inducible and cardiomyocyte-specific Sca1-Cre line for knocking out the PGE2-receptor in these cells.

We thank the reviewer for the suggestions. It would be interesting to learn if a cardiomyocyte-specific Sca1-Cre line would allow investigators to directly examine whether PGE_2 treatment could facilitate cardiomyocyte differentiation of Sca-1^{+} cells. However, construction of a cardiomyocyte-specific Sca-1 Cre line with EP2 knockout would require co-existence of at least two transgenes, Tet-ON and EP2$^{fl/fl}$, to be added onto the M/Z double transgenic mice, which is complicated. On the basis of our data, the number of Sca-1 progenitor cells co-expressing cardiomyocyte marker is relatively low and this type of cells may exist transiently, presented as an intermittent cell type during Sca-1^{+} cell cardiac differentiation. Therefore, even the new lines of transgenic mice can be successfully generated, the results may not be reliable. Nevertheless, this can be regarded as a long-term goal and become possible should one day a new system for genetic fate-mapping of multiple lineages become available.

Referee #2:

Specific comments on the authors' responses:

1a. Satisfactory.

1b. Satisfactory. However, supplementary figure 12b should also include a histogram summarizing the cardiomyogenic potential of Sca1+ cells vs 'small cells' following PGE2 treatment. The Sca1+ cell data for cTnT+ cells is absent.

We thank the reviewer for these comments. Evident sarcomere structure can only be seen in cultured cardiac small cells, demonstrating their cardiomyocyte differentiation potential. Although we detected cTnT expression in Sca-1^{+} cells, we did not observe organized sarcomere structure in these cells. Thus, the cTnT^{+} cells derived from cultured Sca-1^{+} cells were not calculated. To strengthen our results that PGE_2 could improve cardiac stem cell differentiation into cardiomyocytes, the

© EMBO

cardiac small cells and Sca-1^+ cells were isolated and treated with PGE_2 *in vitro*. Following two weeks of culture, we observed beating cells only in the small cell group, the cardiomyocyte-depleted cell population, but not in Sca-1^+ cells (Supporting Information Movie 1). This is possibly because the culture conditions for Sca-1^+ cells were not optimized, or PGE_2-promoted cardiomyocyte differentiation of Sca-1^+ cells may require help from other niche cells. The ability of Sca-1^+ cells to generate beating cells has been reported (Matsuura et al., 2004; Oh et al., 2003). Nevertheless, this requires additional treatment with a drug or protein, but not PGE_2. Moreover, it usually requires a long culture time (more than 3 weeks), and the efficiency is low, approximately 0.1%. After cardiac stem cell isolation and drug treatment, it usually takes at least 1 month to obtain beating cells in previous studies (Matsuura et al., 2004; Oh et al., 2003). Our result demonstrates PGE_2 shortens the time needed to generate beating cells to two weeks, indicating that PGE_2 facilitates the cardiomyocyte differentiation ability of cardiac stem cells in the small cell fraction. Nevertheless, the underlying mechanism of PGE_2-promoted cardiomyocyte differentiation will require further investigation.

Reference:

Matsuura, K., Nagai, T., Nishigaki, N., Oyama, T., Nishi, J., Wada, H., Sano, M., Toko, H., Akazawa, H., Sato, T., et al. (2004). Adult cardiac Sca-1-positive cells differentiate into beating cardiomyocytes. J Biol Chem. 279, 11384-11391.

Oh, H., Bradfute, S.B., Gallardo, T.D., Nakamura, T., Gaussin, V., Mishina, Y., Pocius, J., Michael, L.H., Behringer, R.R., Garry, D.J., et al. (2003). Cardiac progenitor cells from adult myocardium: Homing, differentiation, and fusion after infarction. Proc Natl Acad Sci U S A. 100, 12313-12318.

Result: Page 7

Surprisingly, mature sarcomeric structure and spontaneously beating cells were seen in the cardiomyocyte-depleted small cells after PGE_2 treatment (Supporting Information Fig 12A and B, Supporting Information Movie 1), suggesting that PGE_2 may improve cardiomyocyte differentiation.

1c. Satisfactory.

1d. As stated in previous comments, F4/80 and Gr-1 and/or CD206 must be examined by first gating upon CD45+CD11b+ cells. This has not been conducted (nothing stated in figure legend or Materials and Methods) and the authors have merely stated vaguely that 'appropriate antibodies' have been used. As they are shown, the experiments have not been conducted to a satisfactory standard and are therefore not reliable.

We thank reviewer for this comment and the flow cytometry analysis of M1 and M2 macrophages was performed as suggested. In consistent with previous results we observed that PGE_2 not only lowered the percentage of M1 macrophages ($CD45^+CD11b^+F4/80^+Gr\text{-}1^+$) but also increased the number of M2 macrophages ($CD45^+CD11b^+F4/80^+CD206^+$) after myocardial injury. The data have been incorporated into the resubmitted manuscript in Supporting Information Fig 13.

Result: page 8

Macrophages can be classified into M1 ($CD45^+CD11b^+F4/80^+Gr\text{-}1^+$) and M2 ($CD45^+CD11b^+F4/80^+CD206^+$) subtypes (Nishimura et al, 2009; Vandanmagsar et al, 2011). Interestingly, flow cytometry analysis revealed that PGE_2 treatment elevated the number of M2 macrophages but reduced the number of M1 macrophages after MI (Supporting Information Fig 13A-C).

© EMBO

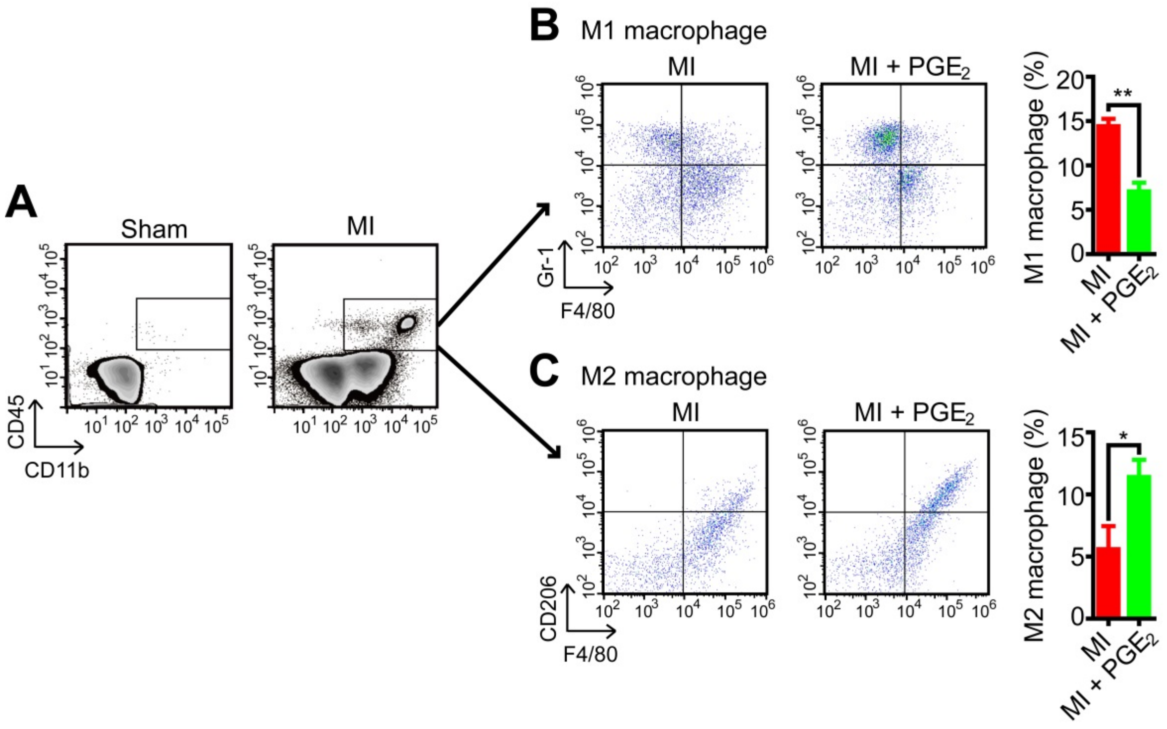

Supporting Information Fig 13. PGE_2 increases the number of M2 type macrophages in the myocardium after injury.

A-C. (A) At day 3 post-surgery, the infarcted heart was enzymatically digested for flow cytometric analysis. The isolated cells were initially gated for expression of both CD45 and CD11b. The double positive cells were further gated into (B) M1 ($F4/80^+Gr\text{-}1^+$) or (C) M2 ($F4/80^+CD206^+$) macrophage. Data are presented as mean ± s.e.m. *$p<0.05$; **$p<0.01$. MI, myocardial infarction.

1e-2d.Satisfactory.

2e. Not satisfactory. The flow cytometry experiment has not been performed as suggested. Therefore, no clear conclusions can be drawn from this experiment.

We thank the reviewer for this comment and the experiment has been repeated as suggested. The data have been incorporated into the revised manuscript in Supporting Information Fig 13.

2f-2h. Satisfactory.

Referee #3:

It is still unclear to this reviewer how a stem cell population contributes 10% new cardiomyocytes. In the paper by Senyo et al. only 3.2% new cardiomyocytes were detected in a time frame of 8 weeks. Even if there is a partial contribution of stem cells it is hard to explain how stem cells generate 10 % new cardiomyocytes.

© EMBO

Assuming the main contribution is from Sca1+ cells it is still unclear how this is possible if the population of Sca1+ cells is only increased 3 days post-MI but already decreased at day 7 post MI? Even more surprising is that these cells should already be differentiated at 3 days post MI to be GFP-positive. This means the cells need to differentiate in 2 days to activate the promoter to produce GFP that it reaches a protein level that can be detected at day 3 post MI. It appears rather likely that these observations are due to cell fusion which has not yet been excluded. Also the in vitro differentiation assays suggest that Sca1+ cannot properly differentiate into cardiomyocytes, even after 10 days. And regarding the small cell population only 1.5% show some kind of differentiation.

We thank the reviewer for raising these questions. Senyo's group observed 3.2 % of new cardiomyocytes were arisen from the proliferated pre-existing GFP^+ cardiomyocytes after MI. Furthermore, they also detected the GFP^+ cardiomyocyte pool was diluted by 15% after MI. Therefore, there is not confliction between the new cardiomyocyte population regenerated by endogenous stem/progenitor cells and the pre-existing cardiomyocytes that have undergone proliferation, which accounts for 10% and 3.2%, respectively. These are two different cell populations possessing varied regenerative ability in response to the heart injury.

Regarding the change in Sca-1^+ cell population, we demonstrated that Sca-1^+ cells were stimulated by MI and underwent differentiation into cardiomyocytes in 7 days post-MI. It is therefore reasonable to observe a reduction in Sca-1 expression at day 7 following injury because these cells have differentiated and lose Sca-1 expression. For flow cytometric analysis of cardiac differentiation ability of the Sca-1^+ cells, the method we have employed would lead to depletion of cardiomyocytes and only the cardiac small cells were isolated. Therefore, the Sca-1^+/GFP^+ cells detected are unlikely to arise from a cell fusion event. Furthermore, the diagram depicting the experiment, as shown in Supporting Information Fig 8, has suggested that the Sca-1^+ cells do not fuse with GFP^+ cells. In consistent with our finding, Senyo's study (Senyo et al, 2013), shown in Figure S4, also provides the same result. Co-expression of stem/progenitor cell and mature cardiomyocyte markers following differentiation has also been observed in the embryonic stem cell culture system. The $GATA4^+$ or Flk^+ cardiac progenitors start to express mature cardiomyocyte markers, such as a-MHC or cTnI, at day 2 following hanging drop (Chen et al, 2010), suggesting existence of the cells co-expressing both stem/progenitor cell and mature cell marker is independent of cell fusion.

References:

Chan SS-K, Li H-J, Hsueh Y-C, Lee DS, Chen J-H, Hwang S-M, Chen C-Y, Shih E, Hsieh PCH (2010) Fibroblast Growth Factor-10 Promotes Cardiomyocyte Differentiation from Embryonic and Induced Pluripotent Stem Cells. PLoS ONE 5: e14414

Senyo SE, Steinhauser ML, Pizzimenti CL, Yang VK, Cai L, Wang M, Wu T-D, Guerquin-Kern J-L, Lechene CP, Lee RT (2013) Mammalian heart renewal by pre-existing cardiomyocytes. Nature 493: 433-436

Result: page 6

In addition, Sca-1^+/α-MHC^+ cells were not observed before tamoxifen labelling and they do not arise from cardiomyocyte de-differentiation or fusion (Hsieh et al, 2007; Senyo et al., 2013) (Supporting Information Fig 8A and B).

© EMBO

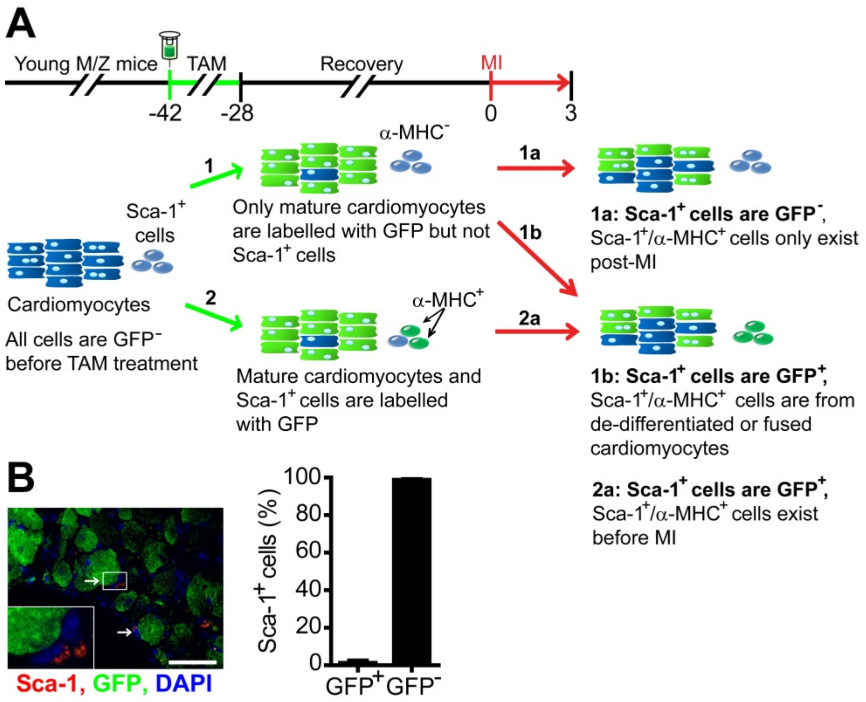

Supporting Information Fig 8. Sca-1^{+}/α-MHC^{+} cells would not be detected before tamoxifen labelling and they do not arise from cardiomyocyte de-differentiation or fusion. .

A. Schematic diagram depicting the experiment that aims to examine if the Sca-1^{+}/α-MHC^{+} cells exist before surgery or they arise from de-differentiated or fused cardiomyocytes after injury. Following 14 days of tamoxifen labelling, the animals were allowed to recover for 1 month prior to myocardial infarction (MI) surgery. On day 3 post-MI, the organ was collected for immunostaining analysis.

B. Representative image of the Sca-1^{+} cells and the original magnifications are as indicated. The Sca-1^{+} cells with or without GFP^{+} signal were quantified. Scale bar, 50 μm. n = 3. Data are presented as the mean ± s.e.m.

A differentiation in vivo has still not been demonstrated.

We agree with the reviewer on this point and the most appropriate system to examine *in vivo* differentiation of stem cells will be Sca-1 cell fate-mapping. Although the experiment is on-going, it will take more than 2 years to complete. Alternatively, we employed flow cytometry to examine cardiomyocyte differentiation ability of the Sca-1^{+} cells by analysing their ability to express mature cardiomyocyte marker (Supporting Information Fig 7). Furthermore, we have also performed Sca-1 cell injection and provided evidence that the cells could give rise to cardiomyocytes independent of cell fusion (Supporting Information Fig 8). Results from both experiments have showed that Sca-1^{+} cells possess cardiomyocyte differentiation ability *in vivo*.

3. Finally the authors perform studies utilizing EP2. How do the authors explain that EP2 is highly elevated at day 7 post MI in the Sca1+ cells in the PGE2 group (60-fold) when the number of Sca1+ cells is dramatically reduced back to the number in the control group?

We thank the reviewer for pointing this out. The figure which the reviewer referred to should be the Supporting Information Figure 9. The 60-fold increase in EP2 gene level was observed in the injured heart tissue located at the border zone and not in the Sca-1^{+} cells.

© EMBO

Comment 2: ok

Comment 3:
The goal of biomedical research is to identify novel strategies to treat disease. Several approaches have already demonstrated to have a better potential to treat heart disease than the present study and many of these studies utilize growth factors, drugs or other strategies that do not require the transplantation of exogenous cells (e.g. miR approach, Neuregulin). Finally, this author would expect that the generation of 10% new cardiomyocytes should have a more profound effect on cardiac function.

We thank the reviewer for raising this question. In the current study we provide evidence demonstrating that systemic administration of PGE_2 could promote endogenous stem cell-dependent heart repair without invasive surgical procedure, such as intramyocardial injection. Although the effect of PGE_2 on improving cardiac outcome was not as effective as other therapies targeting the pre-existing cardiomyocytes, this is the first study showing that the ability of endogenous stem/progenitor cells to repair cardiac injury could be modulated by a pharmacological intervention. A better understanding of the underlying mechanism of PGE_2-governed cardiomyocyte replenishment will aid development of a therapy aiming at treating heart injury more safely and effectively.

Comment 4:
The answer by the authors further suggests to this reviewer that PGE2 has a stem cell independent effect.

Our data suggest PGE_2 is a multi-functional molecule with diverse effects in the injured heart. PGE_2 has been shown to regulate hematopoietic stem cell activity for homeostasis. In different tissue types, i.e. the heart, we also observe PGE_2 could act on stem cells and promote their differentiation ability following injury. In addition, numerous studies have demonstrated PGE_2 can modulate the inflammatory micro-environment, which is consistent with our finding that PGE_2 could govern macrophage polarization. Therefore, regulation of both stem cells and inflammatory micro-environment by PGE_2 to achieve cardiac repair may be two independent machineries that do not conflict with each other.

Comment 5:
If the ejection fraction in PGE2-treated animals at 2 month is not significantly higher than the MI group after 1 month than the treatment appears to have only a protective effect and not a regenerative effect? This suggests again that PGE2 might act stem cell independent?
On the other hand a protective effect is surprising as PGE2 treatment is stopped after 14 days (?). It might be that the stem cells continue to differentiate into a mature phenotype to compensate a continuous loss of cardiomyocytes. However, overall, the effect of this treatment on cardiac function appears negligible.

We agree with the reviewer that the effect of PGE_2 on cardiac outcome is not as profound as other reported therapies aiming to protect cardiomyocytes from apoptosis. However, numerous studies, including our data, have provided evidence that PGE_2 have diverse functions including modulation of stem cell activity and inflammatory micro-environment (Goessling et al, 2009; Nemeth et al, 2009; North et al, 2007).

From the Indomethacin treatment result we discovered that early inflammatory response, within 5 days post-MI, was necessary for stem cell-dependent cardiomyocyte replenishment. Furthermore, we showed that cardiomyocyte regeneration saturated on day 14 post-injury, strengthening the importance of early inflammatory response. On the basis of these findings, we thus designed the experiments for mice with MI to be treated with PGE_2 for 14 days. We believe that understanding the underlying mechanism of how PGE_2 regulates stem cell activity and macrophage polarization may help identify a molecule with better cardiac therapeutic efficacy. Nevertheless, we agree with

© EMBO

the reviewer that the data of cardiac function assessment may be too preliminary and have thus removed it from the resubmitted manuscript.

References:

Goessling W, North TE, Loewer S, Lord AM, Lee S, Stoick-Cooper CL, Weidinger G, Puder M, Daley GQ, Moon RT et al (2009) Genetic interaction of PGE_2 and Wnt signalling regulates developmental specification of stem cells and regeneration. Cell 136: 1136-1147

Nemeth K, Leelahavanichkul A, Yuen PST, Mayer B, Parmelee A, Doi K, Robey PG, Leelahavanichkul K, Koller BH, Brown JM et al (2009) Bone marrow stromal cells attenuate sepsis via prostaglandin E2-dependent reprogramming of host macrophages to increase their interleukin-10 production. Nat Med 15: 42-49

North TE, Goessling W, Walkley CR, Lengerke C, Kopani KR, Lord AM, Weber GJ, Bowman TV, Jang I-H, Grosser T et al (2007) Prostaglandin E2 regulates vertebrate haematopoietic stem cell homeostasis. Nature 447: 1007-1011

Comment 6:
The characterisation of cardiac differentiation needs to be expanded.

We thank the reviewer for this comment. In addition to immunostaining of mature cardiomyocyte markers, as shown in Supporting Information Fig 12, we also obtained beating cardiomyocytes from the cardiomyocyte-depleted cell fraction after PGE_2 treatment. Following 2 weeks of culture, the beating cells could only be found in the small cell group, the cardiomyocyte-depleted cell population, but not in the Sca-1^+ cell alone group, upon PGE_2 treatment (Supporting Information Movie 1). This is possibly because the culture conditions for Sca-1^+ cells are not optimized, or PGE_2-promoted cardiomyocyte differentiation of Sca-1^+ cells may require help from other cells. This could explain why we only obtained beating cells from the small cell fraction. The ability of Sca-1^+ cells to generate beating cells has been reported (Matsuura et al., 2004; Oh et al., 2003). Nevertheless, this requires additional treatment with drug or protein, but not PGE_2. Moreover, it usually takes a long culture time, more than 3 weeks, and the efficiency is low, approximately 0.1%. To avoid confusion, we neither treated the cells with another protein or drug nor cultured the cells for more than 3 weeks. The culture protocol requires further optimization to obtain beating cells from the Sca-1^+ cells.

References:

Matsuura, K., Nagai, T., Nishigaki, N., Oyama, T., Nishi, J., Wada, H., Sano, M., Toko, H., Akazawa, H., Sato, T., et al. (2004). Adult cardiac Sca-1-positive cells differentiate into beating cardiomyocytes. J Biol Chem. 279, 11384-11391.

Oh, H., Bradfute, S.B., Gallardo, T.D., Nakamura, T., Gaussin, V., Mishina, Y., Pocius, J., Michael, L.H., Behringer, R.R., Garry, D.J., et al. (2003). Cardiac progenitor cells from adult myocardium: Homing, differentiation, and fusion after infarction. Proc Natl Acad Sci U S A. 100, 12313-12318.

Comment 7:
See answers to previous comments.

<u>Referee #4</u>:

I have been asked to comment on the authors' response to previous reviewer concerns.

Referee 1

© EMBO

In my view, the reviewer places excessive emphasis on 'contradictory' results between Hsieh Nat Med 2007 and the same group's 2012 Nature study using the same aMHC-MerCre Mer fate-mapping reagents. Keep in mind, the increase in 15N+ myocytes in Lee's paper was far too small to account for the loss of GFP+ ones. The latter data simply do not support the much-cited conclusion that myocyte proliferation predominates in self-repair, and I would not consider stable isotope imaging to be 'more definitive' than fate-mapping until this discrepancy is reconcilted and reproducibility between labs is amply demonstrated.

Conversely, the present reviewer's objection to pulse-chase experiments as misleading can be addressed by a number of precautions, including use of a bistable switch. In the present manuscript, for instance, the observed increase in b-Gal+ myocytes excludes any possibility that loss of GFP was just an artifact of transgene silencing. Work of Malliaras and Marban, recently reported in this journal, give further credibility to the fate-mapping approach.

I am satisfied that Hsueh et al have taken a prudent and balanced approach on this central question of experimental strategy.

Referee 2

The authors have performed the suggested direct comparison of Celecoxib at early vs late intervals after infarction, and the new results confirm their conclusion.

The authors have performed the suggested direct test that PGE2 acts directly on progenitor/stem cells. The new cell culture studies show that PGE2 induces cTnT in purified Sca-1+ cells, though the effect was more striking in a heterogeneous cardiomyocyte-depleted small cell population. The latter result is not fully explained (which cell types are the preferred target?), but this would require much additional work. More importantly, and much more easily addressed, the former result is limited to just cTnT.

We thank the reviewer for these comments. To strengthen our results that PGE_2 improves cardiac stem cell differentiation into cardiomyocytes, the cardiac small cells and Sca-1^+ cells were isolated for PGE_2 treatment *in vitro*. Following 2 weeks of culture, the beating cells could only be observed in the small cell group, the cardiomyocyte-depleted cell fraction, but not in the Sca-1^+ cell alone group, upon PGE_2 treatment (Supporting Information Movie 1). This is possibly because the culture conditions for Sca-1^+ cells are not optimized, or PGE_2-promoted cardiomyocyte differentiation of Sca-1^+ cells requires help from other cells. This could explain why we only obtained beating cells from the small cell fraction. The ability of Sca-1^+ cells to generate beating cells has been reported (Matsuura et al., 2004; Oh et al., 2003). Nevertheless, this requires additional treatment with drug or protein, but not PGE_2. Moreover, it usually requires a long culture time, more than 3 weeks, and the efficiency is low, approximately 0.1%. After cardiac stem cell isolation and drug treatment, it usually takes at least 1 month to obtain beating cells (Matsuura et al., 2004; Oh et al., 2003). Our *in vitro* culture result demonstrates PGE_2 shortens the time needed for generation of beating cells to 2 weeks, indicating that PGE_2 facilitates the cardiomyocyte differentiation ability of cardiac stem cells in the small cell fraction. The underlying mechanism of PGE_2-promoted cardiomyocyte differentiation will require further investigation.

References:

Matsuura, K., Nagai, T., Nishigaki, N., Oyama, T., Nishi, J., Wada, H., Sano, M., Toko, H., Akazawa, H., Sato, T., et al. (2004). Adult cardiac Sca-1-positive cells differentiate into beating cardiomyocytes. J Biol Chem. 279, 11384-11391.

Oh, H., Bradfute, S.B., Gallardo, T.D., Nakamura, T., Gaussin, V., Mishina, Y., Pocius, J., Michael, L.H., Behringer, R.R., Garry, D.J., et al. (2003). Cardiac progenitor cells from adult myocardium: Homing, differentiation, and fusion after infarction. Proc Natl Acad Sci U S A. 100, 12313-12318.

© EMBO

To test directly the mechanism for PGE2's effect, the authors have shown that injected cardiac Sca-1 cells lacking EP2 (the most relevant PGE2 receptor) do not participate in cardiomyocyte repletion. This is a very important addition but there are two issues with the Supplemental Figure 11. In the cartoon, the GFP+ cells should be described as "differentiated" cardiomyocytes, not necessarily "mature" ones. They might well be mononucleated and proliferation-competent. Also, data in the montage must be quantitated and significance shown, as done routinely for other figures.

We thank the reviewer for this suggestion. The figure has been corrected and incorporated in the resubmitted manuscript. Cell quantification has been shown in Figure 2F.

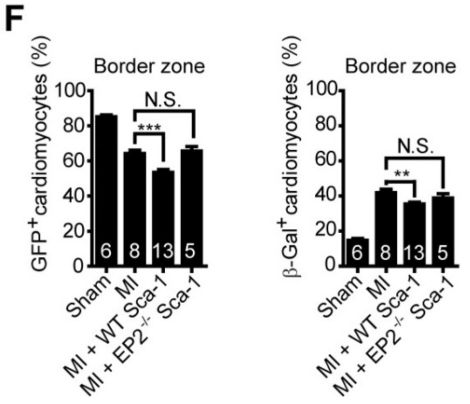

Figure 2. COX-2-dependent signalling pathway stimulates cardiomyocyte replenishment with endogenous stem/progenitor cells shortly after infarction.

F. Following DAB staining, the percentages of GFP^+ and β-Gal^+ cardiomyocytes at the border zone of the young heart with or without cell injection after MI were quantified and statistically analysed. Sample size is indicated in the bar chart. **p< 0.01, ***p< 0.001; N.S., not significant. Data are presented as the mean ± s.e.m.

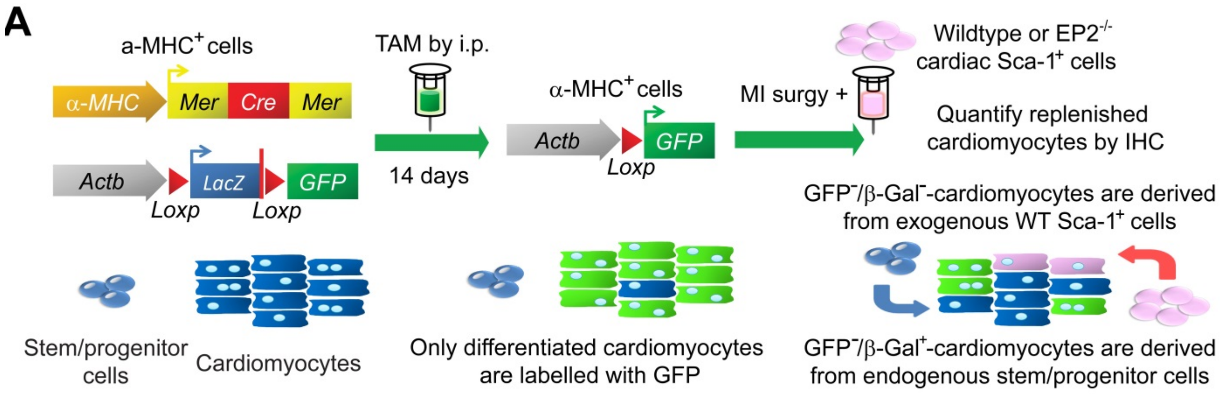

Supporting Information Fig 11. Quantification of the degree of cardiomyocyte replenishment after Sca-1^+ cell injection in injured hearts.

A. Schematic diagram depicting the experimental procedure. Following 14 days of tamoxifen injection, the M/Z mice were injected intramyocardially with wild-type or EP2 knockout ($EP2^{-/-}$) cardiac Sca-1^+ cells after myocardial infarction (MI). The hearts were harvested at day 14 post-MI for examination.

The authors have performed the suggested additional studies of M1 and M2 macrophages, and the improved results support their conclusion.

© EMBO

Flow cytometry analysis of M1 and M2 macrophages was performed as suggested by the reviewer #2. In consistent with previous result we observed that PGE_2 lowered percentage of M1 macrophages($CD45^+CD11b^+F4/80^+Gr\text{-}1^+$) but increased number of M2 macrophages ($CD45^+CD11b^+F4/80^+CD206^+$) after myocardium injury. The data have been incorporated into the revised manuscript in Supporting Information Fig 13.

Result: page 8

Macrophages can be classified into M1 ($CD45^+CD11b^+F4/80^+Gr\text{-}1^+$) and M2 ($CD45^+CD11b^+F4/80^+CD206^+$) subtypes (Nishimura et al, 2009; Vandanmagsar et al, 2011). Interestingly, flow cytometry revealed that PGE_2 treatment elevated the number of M2 macrophages but reduced the number of M1 macrophages after MI (Supporting Information Fig 13A-C).

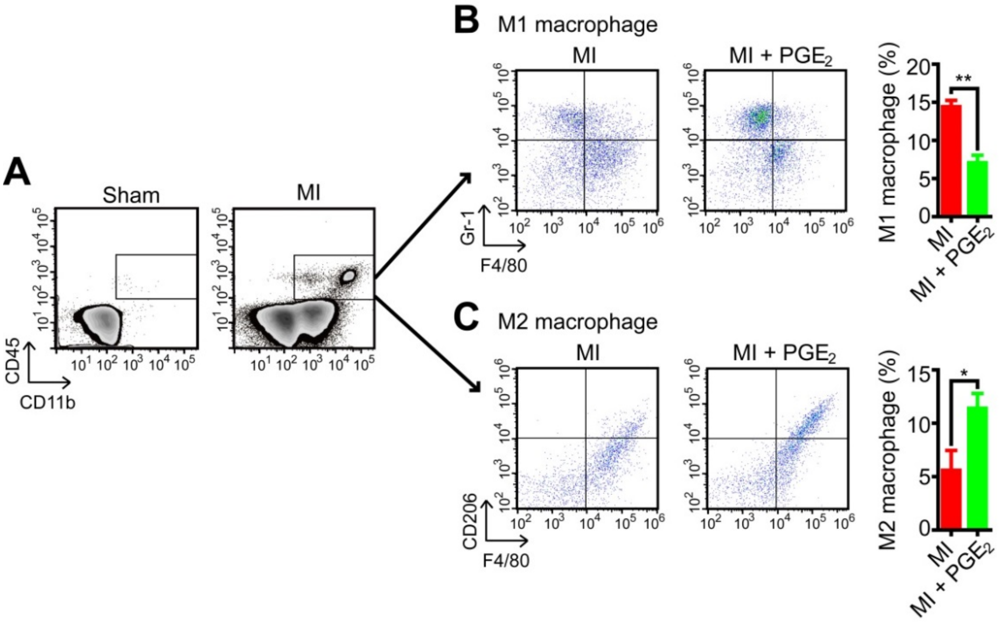

Supporting Information Fig 13. PGE_2 increases the number of M2 type macrophages in the myocardium after injury.

A-C. (A) At day 3 post-surgery, the infarcted heart was enzymatically digested for flow cytometric analysis. The isolated cells were initially gated for expression of both CD45 and CD11b. The double positive cells were further gated into (B) M1 ($F4/80^+Gr\text{-}1^+$) or (C) M2 ($F4/80^+CD206^+$) macrophage. Data are presented as mean ± s.e.m. $^*p<0.05$; $^{**}p<0.01$. MI, myocardial infarction.

Overstatements and caveats have been corrected.

Referee 3

Over-reliance on Senyo is discussed above.

Details of the quantitation have been provided.

Additional comments

I would tone down the claims in the Abstract ('cardiac Sca-1+ cells are the major PGE2-responsive population'), based on Suppl Fig 4 showing induction of Sca-1 mRNA by PGE2, since this experiment doesn't distinguish an expansion of the population vs higher levels in the cells already

© EMBO

expressing Sca-1 vs inducing ectopic expression in Sca-1-negative cells. Minimally, FACS would be needed in addition.

We thank the reviewer for this suggestion and the sentence has been revised.

Abstract: Page 2

Further analyses suggest that cardiac stem cells are PGE_2-responsive and that PGE_2 may regulate stem cell activity directly through the EP2 receptor or indirectly by modulating its micro-environment in vivo.

"no pharmacological treatment is known to promote cardiomyocyte regeneration after injury" - What about thymosin b4 (Smart, Nature 2011), nerve growth factor (Lam PLoS One 2012), nanofiber scaffolds with VEGF (Lin, Sci Transl Med. 2012)?

We thank the reviewer for the comment and the sentence has been revised.

Abstract: Page 2

"few pharmacological treatments are known to promote cardiomyocyte regeneration after injury"

I would also restate the conclusion that "blocking the inflammatory reaction with COX-2 inhibitors reduces the capability of endogenous stem/progenitor cells to repopulate lost cells." The authors' evidence from cell culture and from grafting EP2-null cardiac Sca-1 cells indicate a direct effect of PGE2 on the progenitor cells, unrelated to inflammation. There may or may not be functional importance to the change in M2 macrophages, but these two experiments in the present report show the change in endogenous stem/progenitor cells does not need to involve the inflammatory partners.

We thank the reviewer for this comment. We have toned down the sentence in the conclusion and revised description of Sca-1^+ cells.

Abstract: Page 2
"blocking the inflammatory reaction with COX-2 inhibitors may also reduce the capability of endogenous stem/progenitor cells to repopulate lost cells."

Result: Page 5-6

PGE_2 regulates cardiac stem cell differentiation

To determine whether PGE_2 acts on cardiac stem/progenitor cells to promote cardiomyocyte differentiation, expression level of several known cardiac stem/progenitor cell markers quantified for identification of the PGE_2-responsive gene. We discovered that Sca-1 expression peaked on day 3 post-MI and this level was further increased at the same time point upon PGE_2 treatment but was repressed by Indomethacin (Supporting Information Fig 4). Sca-1 is a common marker co-expressed by several known cardiac stem/progenitor cell populations (Matsuura et al, 2009; Oh et al, 2003; Smart et al, 2011; Sturzu & Wu, 2011), for example c-Kit^+ cell population (Bailey et al, 2012; Rosenblatt-Velin et al, 2012). The c-Kit^+ cells originated from the heart or bone marrow are shown to possess cardiac repair capability (Ellison et al, 2013; Loffredo et al, 2011; Orlic et al, 2001; Rota et al, 2007). However, their ability to repair heart is attenuated upon loss of Sca-1 (Bailey et al, 2012; Rosenblatt-Velin et al, 2012). Furthermore, we observed that the expression pattern of the cardiac progenitor cell marker Nkx2.5 (Wu et al, 2006) is similar to that of Sca-1 (Supporting Information Fig 5). Furthermore, PGE_2 also elevated the expression of Nkx2.5 in Sca-1^+ cells (Supporting Information Fig 6). We therefore sought to investigate the effect of PGE_2 on stem cell-mediated cardiomyocyte replenishment by examining Sca-1^+ cell activities.

© EMBO

References: Page 21-24

Bailey B, Fransioli J, Gude NA, Alvarez R, Zhan X, Gustafsson ÅB, Sussman MA (2012) Sca-1 knockout impairs myocardial and cardiac progenitor cell function / novelty and significance. Circ Res 111: 750-760

Ellison Georgina M, Vicinanza C, Smith Andrew J, Aquila I, Leone A, Waring Cheryl D, Henning Beverley J, Stirparo Giuliano G, Papait R, Scarfò M et al (2013) Adult c-kitpos cardiac stem cells are necessary and sufficient for functional cardiac regeneration and repair. Cell 154: 827-842

Rosenblatt-Velin N, Ogay S, Felley A, Stanford WL, Pedrazzini T (2012) Cardiac dysfunction and impaired compensatory response to pressure overload in mice deficient in stem cell antigen-1. FASEB J 26: 229-239

4th Editorial Decision 12 December 2013

Thank you for the submission of your revised manuscript to EMBO Molecular Medicine. We have asked two editorial advisers to look at your manuscript and I am happy to say that they both support publication. We will be able to accept your article in principle, pending editorial final amendments.

I look forward to reading a new revised version of your manuscript as soon as possible.

© EMBO
