# Supplementary material for: Prostaglandin E2 promotes post-infarction cardiomyocyte replenishment by endogenous stem cells
Source: EMBO Mol Med. 2014 Jan 21;6(4):496–503. doi: 10.1002/emmm.201303687 (PMC3992076; doi:10.1002/emmm.201303687)
Supplement: Supplementary file 2 [file emmm0006-0496-sd2.pdf]

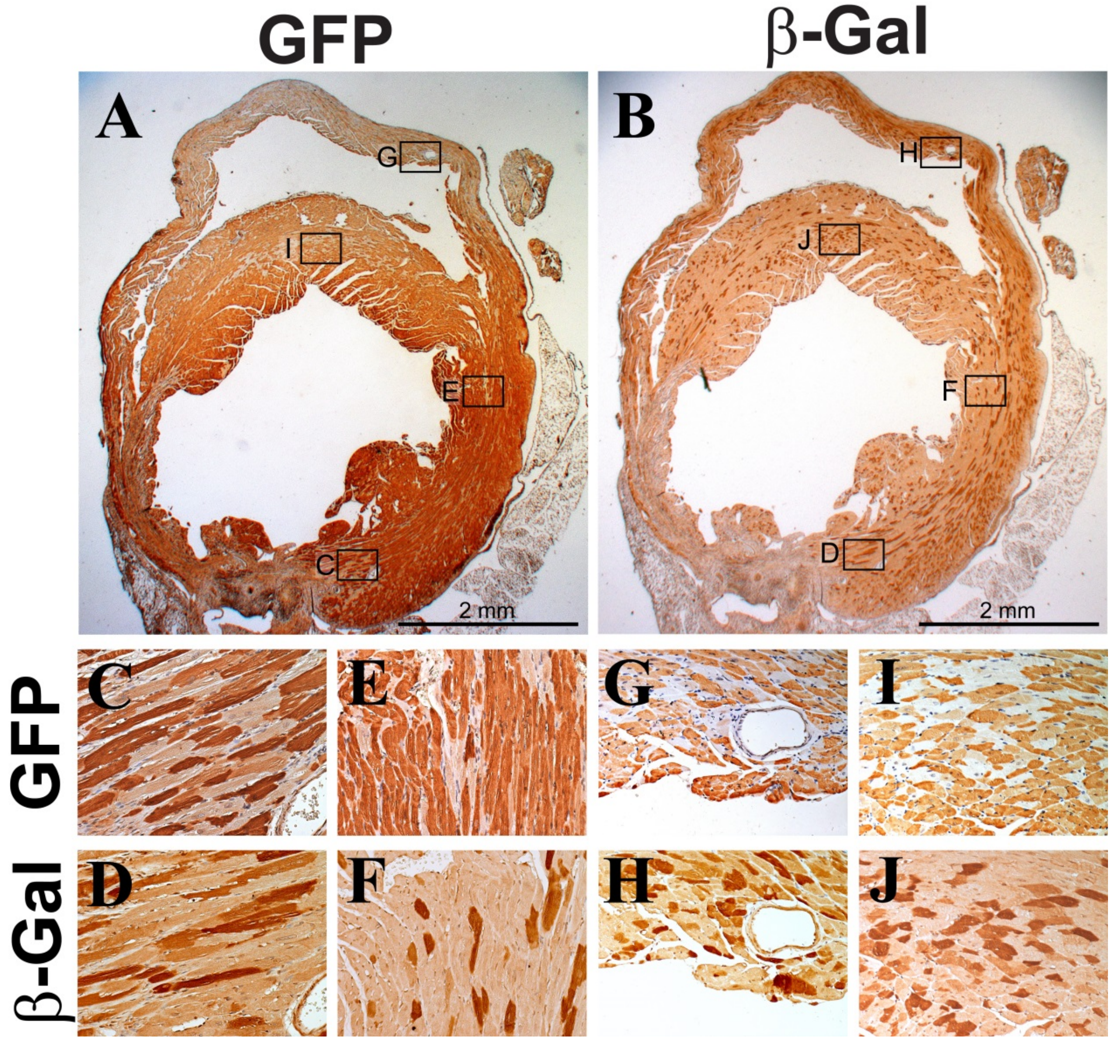

Supporting Information Fig 1. Distribution of GFP$^+$ or β-Gal$^+$ cardiomyocytes in the heart at day 14 after myocardial infarction.

A-B. The heart at day 14 post-infarction was subjected to GFP or β-Gal staining. The images of whole heart cross-sections were taken at low magnification.

C-J. The specified images were taken from the border zone, remote area of the scar site, the interventricular septum and the right ventricle under higher magnification.
