# Supplementary material for: Prostaglandin E2 promotes post-infarction cardiomyocyte replenishment by endogenous stem cells
Source: EMBO Mol Med. 2014 Jan 21;6(4):496–503. doi: 10.1002/emmm.201303687 (PMC3992076; doi:10.1002/emmm.201303687)
Supplement: Supplementary file 3 [file emmm0006-0496-sd3.pdf]

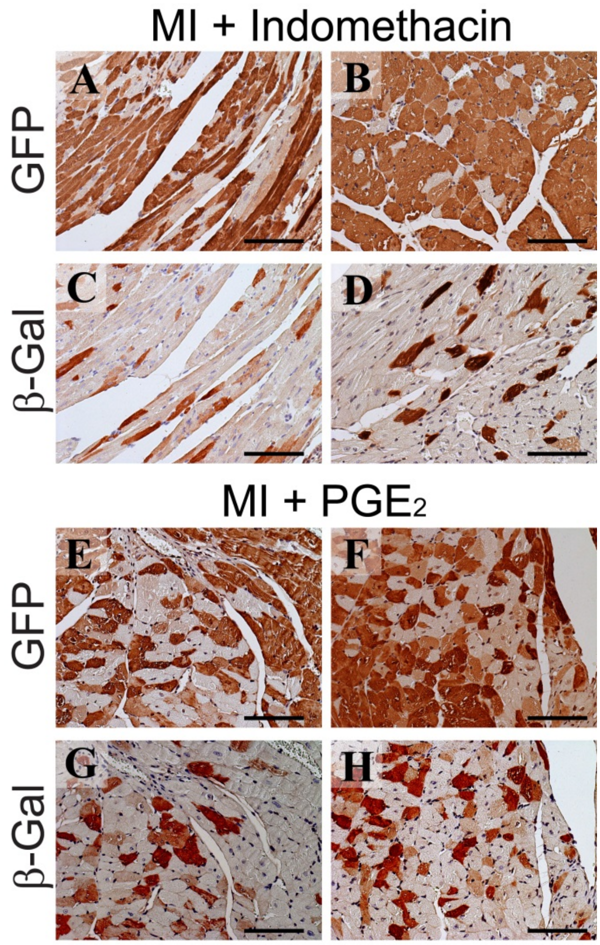

I

	DAB^+ cell count		total
GFP	A = 76	B = 113	189
β-Gal	C = 30	D = 28	58
GFP	E = 75	F = 92	167
β-Gal	G = 47	H = 60	107

Supporting Information Fig 2. The number of β-Gal^+ cardiomyocytes increases after PGE_2 treatment on day 14 post-infarction.

A-H. The heart sections of Indomethacin- or PGE_2-treated mice on day 14 post-infarction were stained for GFP or β-Gal and the photos were taken from three locations at the border zone.

I. DAB^+ cardiomyocytes in each photo were quantified and the sum represents the total number of cells from three photos. Scale bars, 100 μm.
