# Supplementary material for: Prostaglandin E2 promotes post-infarction cardiomyocyte replenishment by endogenous stem cells
Source: EMBO Mol Med. 2014 Jan 21;6(4):496–503. doi: 10.1002/emmm.201303687 (PMC3992076; doi:10.1002/emmm.201303687)
Supplement: Supplementary file 4 [file emmm0006-0496-sd4.pdf]

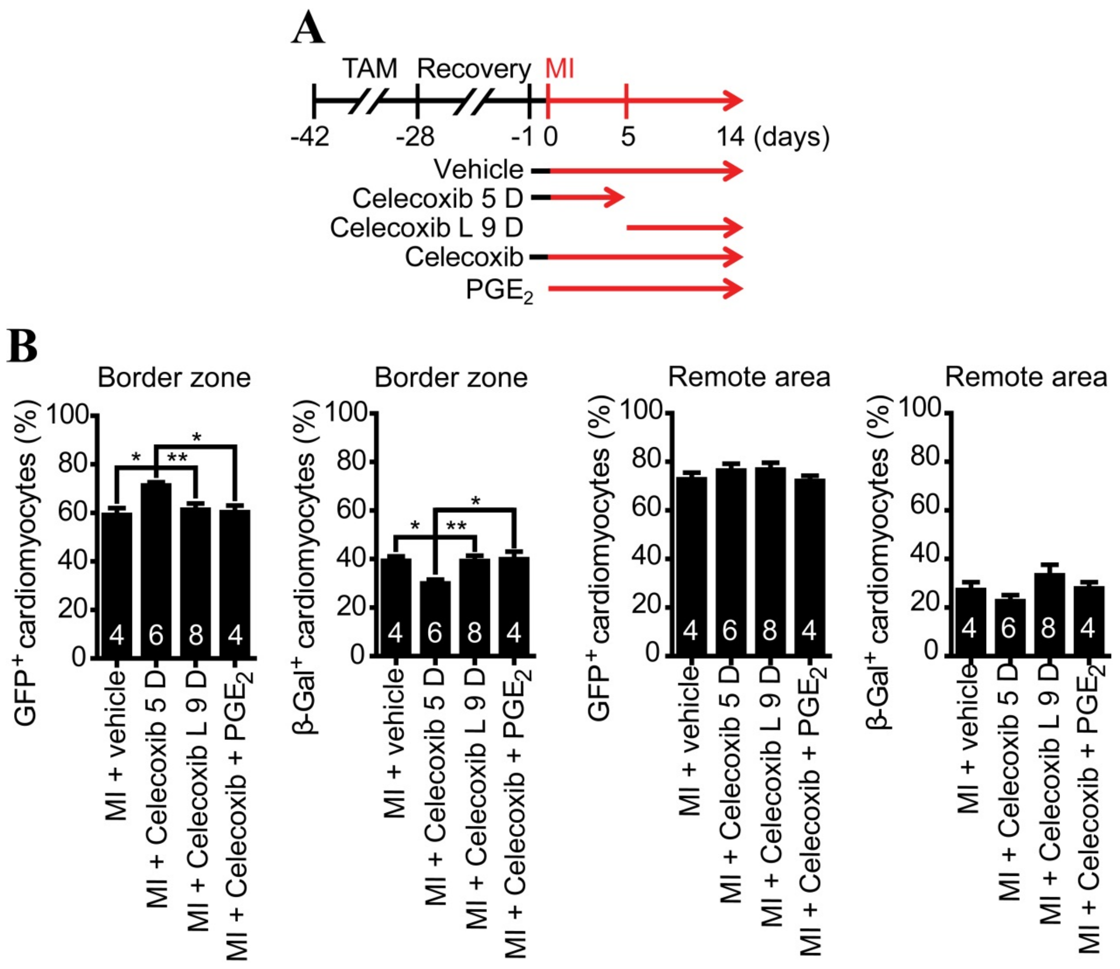

Supporting Information Fig 3. Early COX-2 signalling pathway is necessary for cardiomyocyte regeneration.

A. Experimental paradigm of drug treatment on the MerCreMer/ZEG mice. Celecoxib was continuously administered 1 day before surgery until day 5 (Celecoxib 5 D) after MI or from day 5 to day 14 post-MI (Celecoxib L 9 D). Another group of animals was administrated with Celecoxib and PGE_2 simultaneously for 14 days. The hearts from drug-treated mice isolated at day 14 post-infarction were stained for GFP or β-Gal. TAM, tamoxifen.

B. GFP^+ and β-Gal^+ cells at the border zone or remote area were quantified and statistically analyzed. *p<0.05, **p<0.01. Data are presented as the mean ± s.e.m. Sample size is indicated in the bar chart. MI, myocardial infarction.
