# Supplementary material for: Prostaglandin E2 promotes post-infarction cardiomyocyte replenishment by endogenous stem cells
Source: EMBO Mol Med. 2014 Jan 21;6(4):496–503. doi: 10.1002/emmm.201303687 (PMC3992076; doi:10.1002/emmm.201303687)
Supplement: Supplementary file 5 [file emmm0006-0496-sd5.pdf]

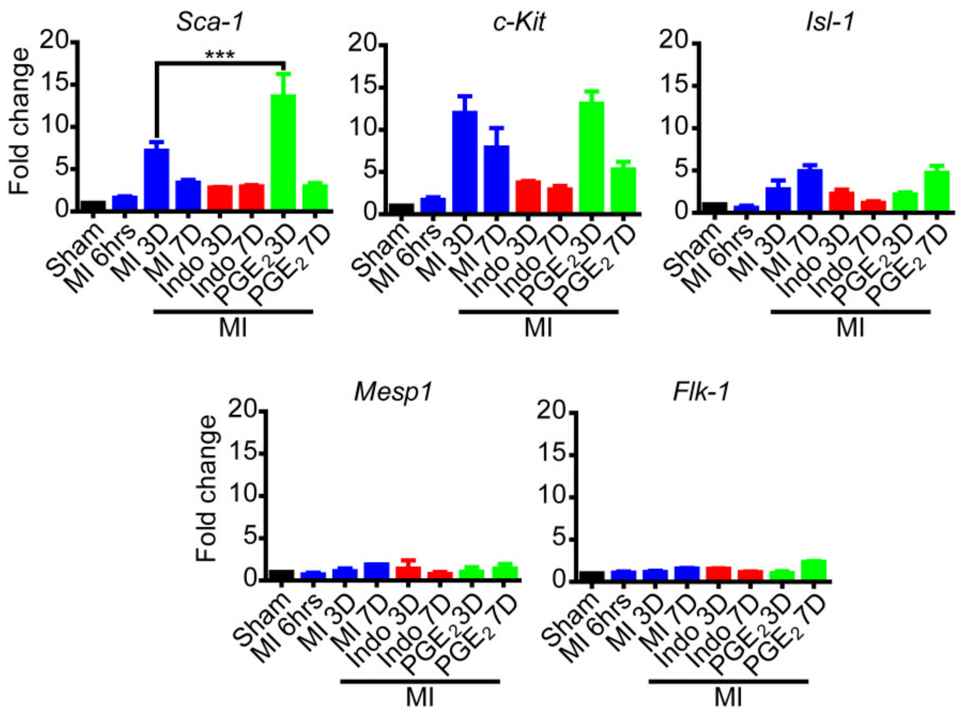

Supporting Information Fig 4. Gene expression of *Sca-1* is the most responsive to PGE_2 treatment.

Expression of the cardiac stem/progenitor marker genes in the infarcted region of injured hearts was analyzed by quantitative RT-PCR. The fold change is a relative quantification normalized to the sham control. ***$p < 0.001$. $n \geq 3$. Data are presented as the mean ± s.e.m. Indo, Indomethacin; MI, myocardial infarction.
