# Supplementary material for: Prostaglandin E2 promotes post-infarction cardiomyocyte replenishment by endogenous stem cells
Source: EMBO Mol Med. 2014 Jan 21;6(4):496–503. doi: 10.1002/emmm.201303687 (PMC3992076; doi:10.1002/emmm.201303687)
Supplement: Supplementary file 6 [file emmm0006-0496-sd6.pdf]

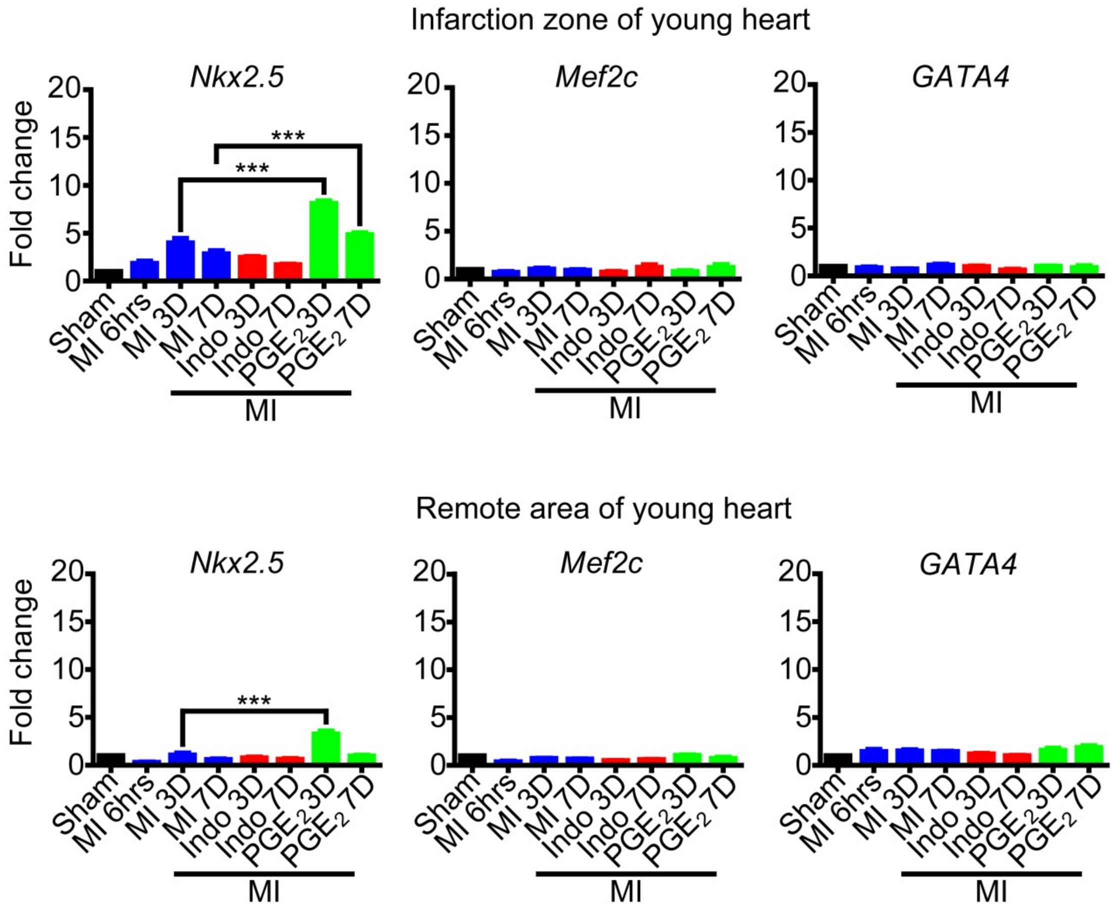

Supporting Information Fig 5. Gene expression analysis of cardiac transcription factor marker genes after myocardial infarction in response to different drug treatments.

Expression of the cardiac transcription factor genes in both infarcted and remote regions of injured hearts was analyzed by quantitative RT-PCR. The fold change is a relative quantification normalized to the sham control. ***$p< 0.001$. $n\geq3$. Data are presented as the mean ± s.e.m. Indo, Indomethacin; MI, myocardial infarction.
