# Supplementary material for: Prostaglandin E2 promotes post-infarction cardiomyocyte replenishment by endogenous stem cells
Source: EMBO Mol Med. 2014 Jan 21;6(4):496–503. doi: 10.1002/emmm.201303687 (PMC3992076; doi:10.1002/emmm.201303687)
Supplement: Supplementary file 7 [file emmm0006-0496-sd7.pdf]

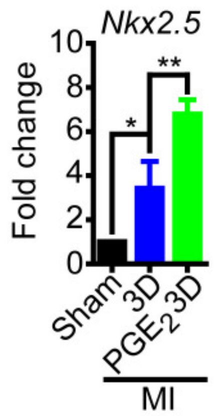

Supporting Information Fig 6. PGE_2 augments expression of *Nkx2.5* in the Sca-1$^+$ cells of injured heart.

At day 3 post-surgery, the Sca-1$^+$ cells from the heart treated with or without PGE_2 were isolated for quantitative RT-PCR analysis for *Nkx2.5* expression. The fold change is a relative quantification normalized to the sham control. *$p<0.05$, **$p<0.01$. $n \geq 3$. Data are presented as the mean ± s.e.m. MI, myocardial infarction.
