# Supplementary material for: Prostaglandin E2 promotes post-infarction cardiomyocyte replenishment by endogenous stem cells
Source: EMBO Mol Med. 2014 Jan 21;6(4):496–503. doi: 10.1002/emmm.201303687 (PMC3992076; doi:10.1002/emmm.201303687)
Supplement: Supplementary file 8 [file emmm0006-0496-sd8.pdf]

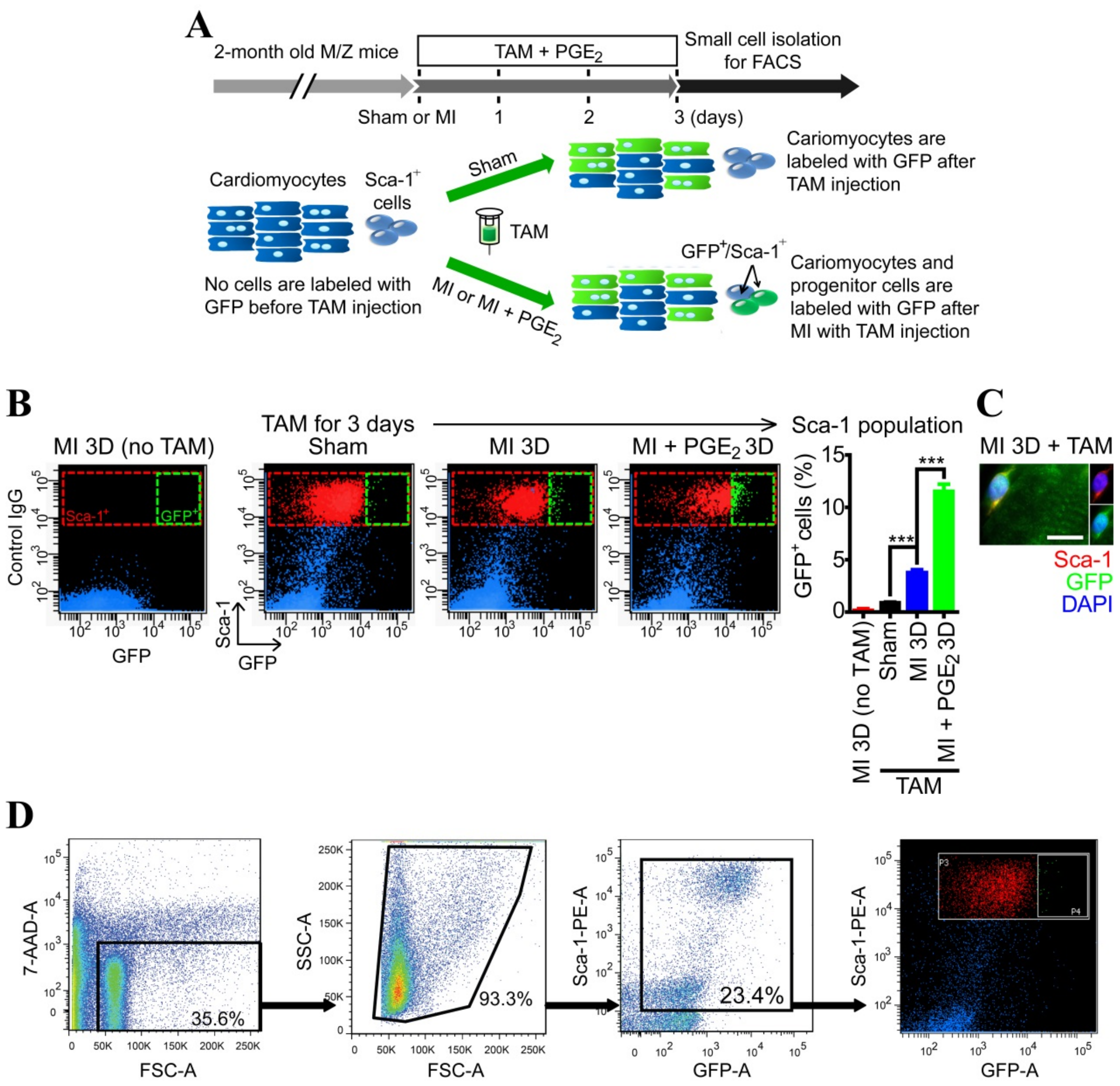


**Supporting Information Fig 7. Examination of cardiomyogenic differentiation ability of cardiac Sca-1$^{+}$ cells after myocardial infarction.**

A. Schematic diagram depicting the experimental procedure. Following myocardial infarction (MI) surgery, the M/Z mice were injected with 80 μg/g tamoxifen (TAM) per day for 3 days with or without an additional $PGE_2$ treatment. The sham control was also treated with the same dosage of tamoxifen and $PGE_2$ simultaneously for 3 days. The cardiac small cells were isolated and subjected to flow cytometric analysis of Sca-1$^{+}$/GFP$^{+}$ cells at day 3 post-surgery.

B. The percentages of Sca-1$^{+}$/GFP$^{+}$ cells at day 3 post-MI were quantified by flow cytometry. Mice that did not receive tamoxifen injections after the MI surgery served as negative control (no TAM). The number of Sca-1$^{+}$/GFP$^{+}$ cells was also quantified. The data are presented as percentage calculated by dividing the number of double-positive cells by the total number of Sca-1$^{+}$ cells. ***$p$<0.001. Data are presented as the mean ± s.e.m. n≥ 4.
