# Supplementary material for: Prostaglandin E2 promotes post-infarction cardiomyocyte replenishment by endogenous stem cells
Source: EMBO Mol Med. 2014 Jan 21;6(4):496–503. doi: 10.1002/emmm.201303687 (PMC3992076; doi:10.1002/emmm.201303687)
Supplement: Supplementary file 9 [file emmm0006-0496-sd9.pdf]

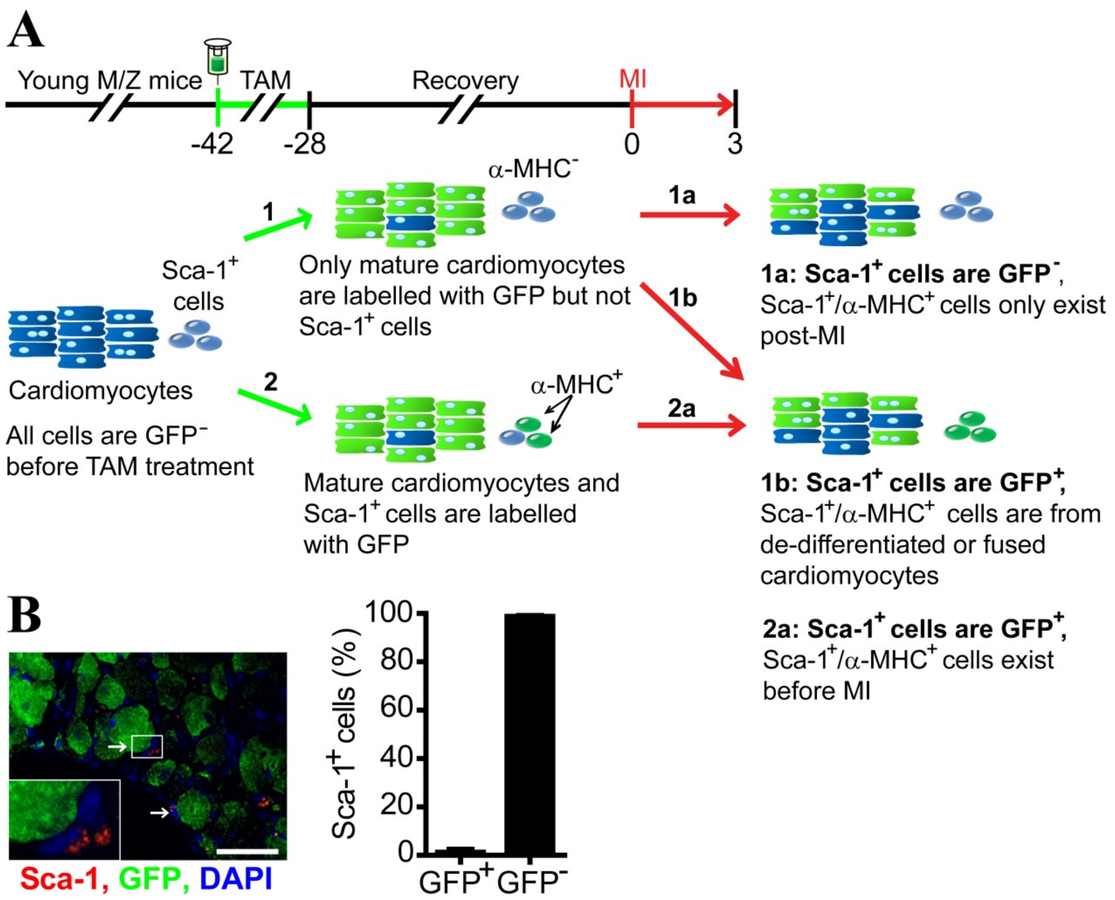

Supporting Information Fig 8. Sca-1^{+}/α-MHC^{+} cells would not be detected before tamoxifen labeling and they do not arise from cardiomyocyte de-differentiation or fusion. .

A. Schematic diagram depicting the experiment that aims to examine if the Sca-1^{+}/α-MHC^{+} cells exist before surgery or they arise from de-differentiated or fused cardiomyocytes after injury. Following 14 days of tamoxifen (TAM) labeling, the animals were allowed to recover for 1 month prior to myocardial infarction (MI) surgery. On day 3 post-MI, the organ was collected for immunostaining analysis.

B. Representative image of the Sca-1^{+} cells and the original magnifications are as indicated. The Sca-1^{+} cells with or without GFP^{+} signal were quantified. Scale bar, 50 μm. n=3. Data are presented as the mean ± s.e.m.
