# Supplementary material for: Prostaglandin E2 promotes post-infarction cardiomyocyte replenishment by endogenous stem cells
Source: EMBO Mol Med. 2014 Jan 21;6(4):496–503. doi: 10.1002/emmm.201303687 (PMC3992076; doi:10.1002/emmm.201303687)
Supplement: Supplementary file 10 [file emmm0006-0496-sd10.pdf]

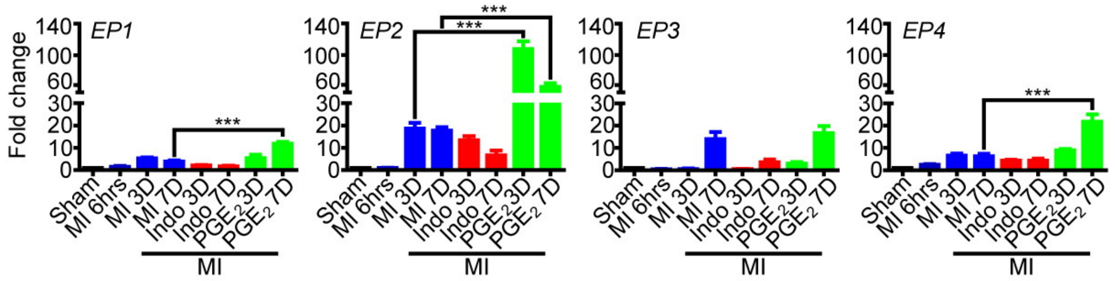

Supporting Information Fig 9. PGE_2 increases the expression of *EP2* receptor after myocardial infarction.

The expression of PGE_2 receptors, *EP1, 2, 3 and 4*, in response to different drug treatments at the infarct region of the injured heart was examined by quantitative RT-PCR. The fold change is a relative quantification normalized to the sham control. ***$p<0.001$. $n\geq3$. Data are presented as mean ± s.e.m. Indo, Indomethacin; MI, myocardial infarction.
