# Supplementary material for: Prostaglandin E2 promotes post-infarction cardiomyocyte replenishment by endogenous stem cells
Source: EMBO Mol Med. 2014 Jan 21;6(4):496–503. doi: 10.1002/emmm.201303687 (PMC3992076; doi:10.1002/emmm.201303687)
Supplement: Supplementary file 11 [file emmm0006-0496-sd11.pdf]

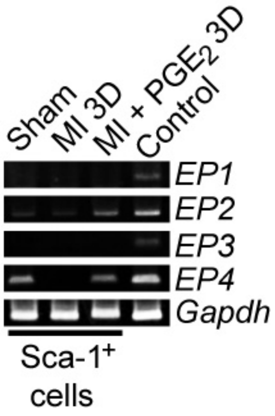

Supporting Information Fig 10. The expression of *EP2* receptor increases in Sca-1$^+$ cells isolated from the infarcted heart after PGE_2 treatment.

Semi-quantitative PCR was performed to examine the expression of PGE_2 receptors, *EP1, 2, 3* and *4*, in cardiac Sca-1$^+$ cells isolated after myocardial infarction (MI). Un-sorted cardiomyocyte-depleted small cells from the sham group served as positive control. n≥3.
