# Supplementary material for: Prostaglandin E2 promotes post-infarction cardiomyocyte replenishment by endogenous stem cells
Source: EMBO Mol Med. 2014 Jan 21;6(4):496–503. doi: 10.1002/emmm.201303687 (PMC3992076; doi:10.1002/emmm.201303687)
Supplement: Supplementary file 12 [file emmm0006-0496-sd12.pdf]

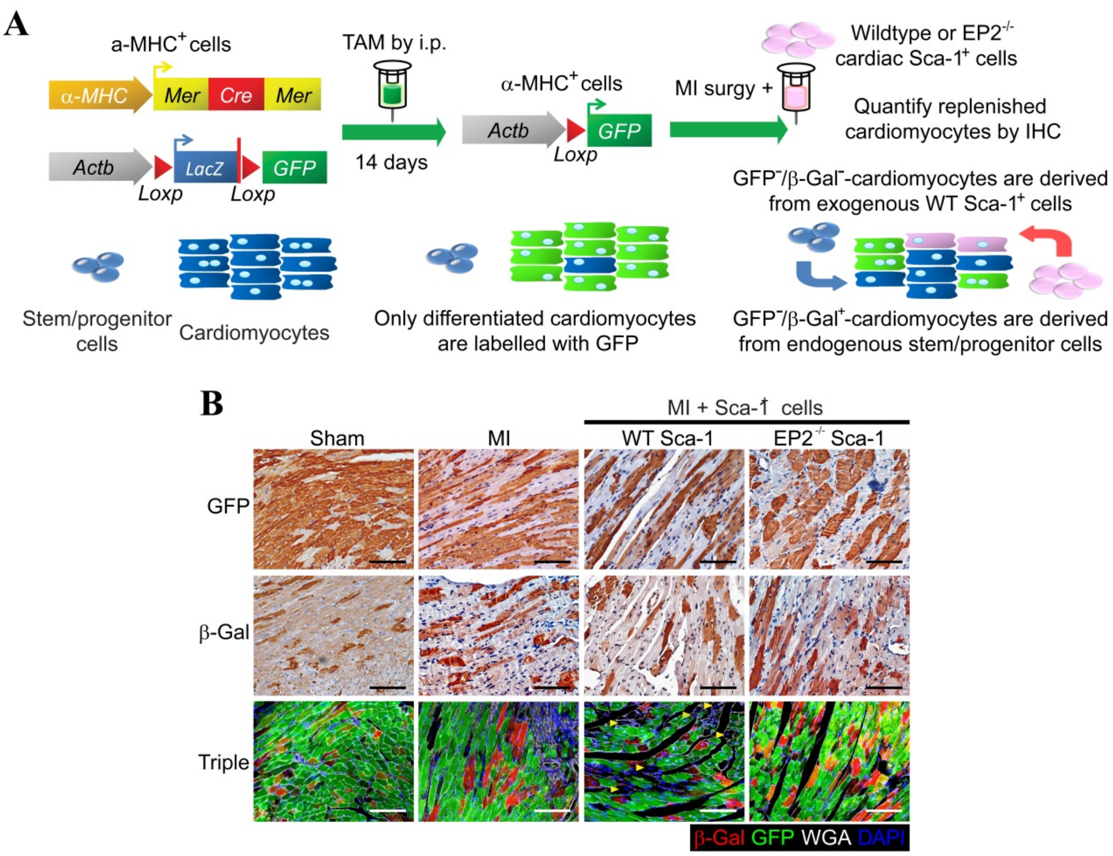


**Supporting Information Fig 11. Quantification of the degree of cardiomyocyte replenishment after Sca-1$^{+}$ cell injection in injured hearts.**

A. Schematic diagram depicting the experimental procedure. Following 14 days of tamoxifen (TAM) injection, the M/Z mice were injected intramyocardially with wild-type or EP2 knockout (EP2$^{-/-}$) cardiac Sca-1$^{+}$ cells after myocardial infarction (MI). The hearts were harvested at day 14 post-MI for examination.
B. At day 14 post-infarction, the hearts were harvested for DAB and immunofluorescence triple staining to examine the GFP$^{+}$ or β-Gal$^{+}$ cardiomyocytes. Shown are representative images from each group. Scale bars, 100 μm.
