# Supplementary material for: Prostaglandin E2 promotes post-infarction cardiomyocyte replenishment by endogenous stem cells
Source: EMBO Mol Med. 2014 Jan 21;6(4):496–503. doi: 10.1002/emmm.201303687 (PMC3992076; doi:10.1002/emmm.201303687)
Supplement: Supplementary file 13 [file emmm0006-0496-sd13.pdf]

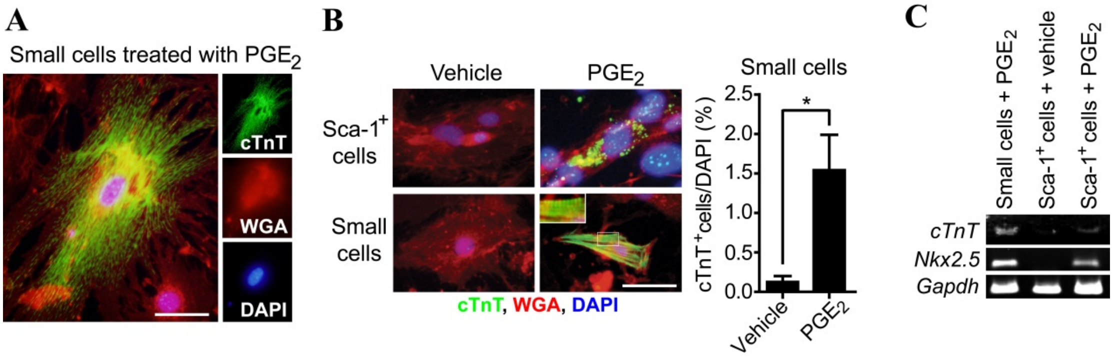

Supporting Information Fig 12. *In vitro* differentiation ability of isolated cardiac cells into cardiomyocytes.

A. The cardiomyocyte-depleted small cells were allowed to attach for 3 days followed by PGE_2 treatment (10 μM) for another 3 days. Immunocytochemistry was performed to examine cardiomyocyte differentiation, as determined by the expression of the cardiomyocyte marker cardiac troponin T (cTnT) at day 10. The membrane and nucleus were stained with the membrane dye, WGA, and DAPI, respectively. Scale bar, 50 μm.

B. Under the same culture condition, the cardiac Sca-1^+ cells and small cells subjected to vehicle or PGE_2 treatment were stained with cTnT for sarcomeric structure analysis. The percentage of small cells with mature sarcomeric structures following vehicle or PGE_2 treatment was quantified. Scale bar, 50 μm. $^*p< 0.05$. Data are presented as mean ± s.e.m.

C. Following the same culture procedure, the effect of PGE_2 on the expression of cardiac marker genes, *Nkx2.5* and *cTnT*, in Sca-1^+ cells was analyzed by semi-quantitative PCR. Small cells treated with PGE_2 serve as positive control.
