# Supplementary material for: Prostaglandin E2 promotes post-infarction cardiomyocyte replenishment by endogenous stem cells
Source: EMBO Mol Med. 2014 Jan 21;6(4):496–503. doi: 10.1002/emmm.201303687 (PMC3992076; doi:10.1002/emmm.201303687)
Supplement: Supplementary file 14 [file emmm0006-0496-sd14.pdf]

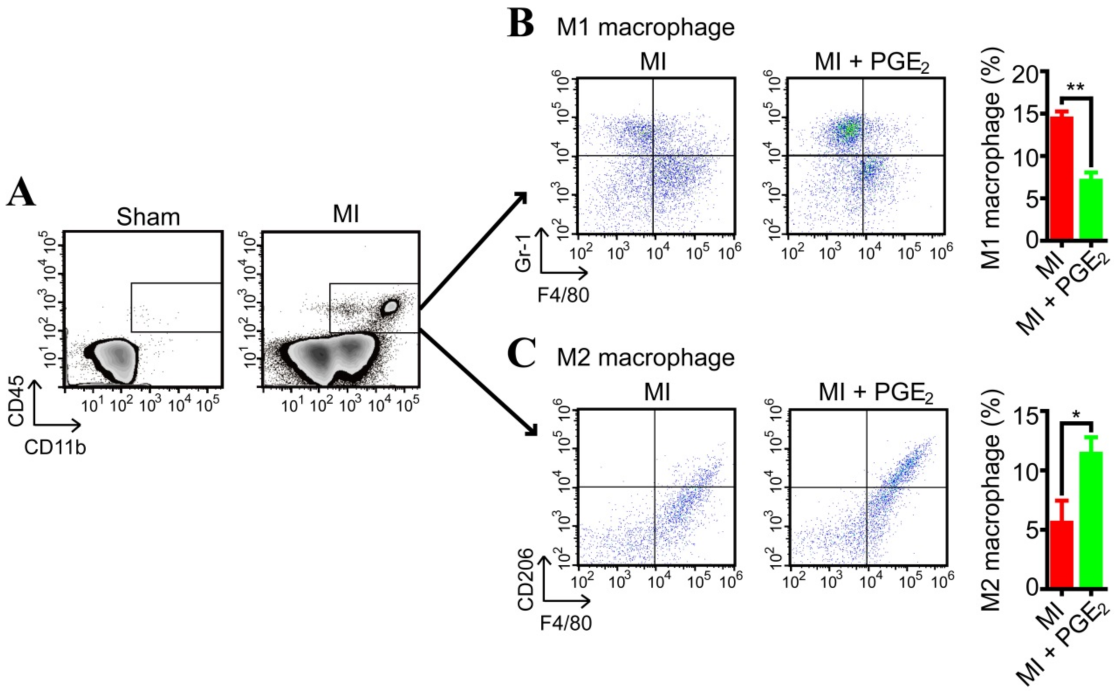

Supporting Information Fig 13. PGE_2 increases the number of M2 type macrophages in the myocardium after injury.

A-C. (A) At day 3 post-surgery, the infarcted heart was enzymatically digested for flow cytometric analysis. The isolated cells were initially gated for expression of both CD45 and CD11b. The double positive cells were further gated into (B) M1 ($F4/80^+Gr\text{-}1^+$) or (C) M2 ($F4/80^+CD206^+$) macrophage. Data are presented as mean ± s.e.m. *$p<0.05$; **$p<0.01$. MI, myocardial infarction.
