# Supplementary material for: Prostaglandin E2 promotes post-infarction cardiomyocyte replenishment by endogenous stem cells
Source: EMBO Mol Med. 2014 Jan 21;6(4):496–503. doi: 10.1002/emmm.201303687 (PMC3992076; doi:10.1002/emmm.201303687)
Supplement: Supplementary file 15 [file emmm0006-0496-sd15.pdf]

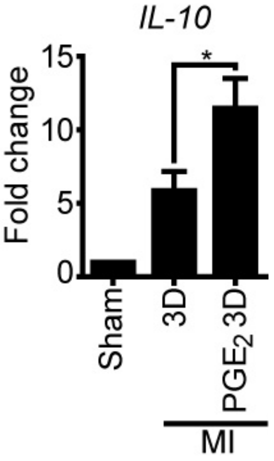

Supporting Information Fig 14. PGE_2 induces *IL-10* level after infarction.
Real-time RT-PCR analysis of expression of *IL-10* in response to PGE_2 treatment on day 3 after infarction at the injured region of young mice. Data are presented as mean ± s.e.m. *$p < 0.05$. n≥3.
