# Supplementary material for: Prostaglandin E2 promotes post-infarction cardiomyocyte replenishment by endogenous stem cells
Source: EMBO Mol Med. 2014 Jan 21;6(4):496–503. doi: 10.1002/emmm.201303687 (PMC3992076; doi:10.1002/emmm.201303687)
Supplement: Supplementary file 17 [file emmm0006-0496-sd17.pdf]

Supporting Information Table 1:
List of primers for quantitative or semi-quantitative PCR

Gene name	Forward 5'-3'	Reverse 5'-3'
c-Kit	CTCACATAGCAGGGAGCACA	ACAACTCACCCACACGCATA
Sca-1	ACCTCCACCCTTGTCCTTTT	CTTCACTGTGCTGGCTGTGT
Mesp1	CTGAAGAGTGGAGGGGACAA	CAAGGAGGGTTGGAATGGTA
Flk-1	TCTACTGGGTTAGCCTGTAAAC	TCTGTCTGGCTGTCATCTGG
Mef2c	ATGCCATCAGTGAATCAAAGGAT	GTGGTACGGTCTCCCAACT
Isl-1	CAGAGTCATCCGAGTGTGG	TCTACTGGGTTAGCCTGTAAAC
Nkx2.5	GAGCCTGGTAGGGAAAGAGC	TCTGAGGGACAGGGCATAGT
GATA4	TCAAACCAGAAAACGGAAGC	CTGCTGTGCCCATAGTGAGA
cTnT	CAGAGGAGGCCAACGTAGAAG	TCGATCAGAGTCTGTAGCTCATT
IL-10	AGCAAGGCAGTGGAGCAGGT	GGAACTGAGGTATCAGAGGTAA
TGF-β1	CGCAACAACGCCATCTAT	CCAAGGTAACGCCAGGAAT
EP1	CGCAGGGTTCACGCACACGA	CACTGTGCCGGGAACTACGC
EP2	AGGACTTCGATGGCAGAGGAGA	CAGCCCCTTACACTTCTCCAATG
EP3	CATGATGGTCACTGGCTTCGT	GTCACCACCAGAGCCAGCAAG
EP4	TGCTTCTGTGAACCCCATC	GAGGTGGTGTCTGCTTGGGTCAG
Gapdh	AACGACCCCTTCATTGAC	TCCACGACATACTCAGCAC
