# Supplementary material for: Prostaglandin E2 promotes post-infarction cardiomyocyte replenishment by endogenous stem cells
Source: EMBO Mol Med. 2014 Jan 21;6(4):496–503. doi: 10.1002/emmm.201303687 (PMC3992076; doi:10.1002/emmm.201303687)
Supplement: Supplementary file 18 [file emmm0006-0496-sd18.pdf]

Supporting Information

Prostaglandin E2 Promotes Post-Infarction Cardiomyocyte Replenishment by Endogenous Stem Cells

Ying-Chang Hsueh, Jasmine M.F. Wu, Chun-Keung Yu,

Kenneth K. Wu, and Patrick C. H. Hsieh

Table of Content

Supporting Information Figures 1-14

Supporting Information Table 1

Supporting Information Movie 1

Supporting Information Movie 1. Small cell-derived beating cells following PGE_2 treatment. Small cells isolated from the heart were treated with PGE_2 for 3 days. Beating cell can be observed on day 14 after isolation.
